# Tranexamic acid for anemia in women of reproductive age: A systematic review of randomized trials

**DOI:** 10.1371/journal.pone.0362488

**Published:** 2026-11-06

**Authors:** Sima Berendes, Katharine Ker, Hilary O. D. Critchley, Raoul Mansukhani, Ian Roberts

**Affiliations:** 1 Clinical Trials Unit, Faculty of Epidemiology and Population Health, London School of Hygiene & Tropical Medicine, Bloomsbury, London, United Kingdom; 2 Centre for Reproductive Health, Institute for Regeneration and Repair, University of Edinburgh, Edinburgh, United Kingdom; Teikyo University: Teikyo Daigaku, JAPAN

## Abstract

**Objective:**

The global target to halve the prevalence of anemia by 2025 has not been met. New strategies are needed. This systematic review examined the effect of tranexamic acid on anemia, menstrual health and wellbeing in women of reproductive age.

**Methods:**

We searched six databases from inception to 30 August 2025 and reference lists of seven related reviews. We included randomized trials comparing the effects of tranexamic acid with placebo or standard care on uterine bleeding. Eligible participants had normal menstrual bleeding or any type of non-pregnant abnormal uterine bleeding. Outcomes included hemoglobin concentration and anemia prevalence as the primary outcome, estimated blood loss, quality of life, thromboembolic events, and other adverse events. We conducted meta-analyses and assessed risk of bias (Cochrane risk-of-bias tool for randomized trials) and the certainty of evidence.

**Results:**

We included 17 trials in the review and 15 in the meta-analyses. None of the trials reported adequate data on hemoglobin concentration. For the anemia outcome, only one small trial contributed data (risk ratio 0.15, 95% confidence interval 0.01 to 2.74, very low certainty). Meta-analyses of secondary outcomes showed that tranexamic acid reduced objectively measured menstrual blood loss among women with heavy menstrual bleeding (6 trials, mean difference −47.0 milliliters, 95% confidence interval −56.6 to −37.4, I^2^ = 13%, moderate certainty) and in women with intrauterine devices (3 trials, mean difference −41.6 milliliters, 95% confidence interval −52.2 to −30.9, I^2^ = 64%, low certainty) and improved quality of life in women with heavy menstrual bleeding (2 trials, mean difference 0.5, 95% confidence interval 0.4 to 0.7, I^2^ = 0%, moderate certainty). The risk of nausea or vomiting was similar between groups (4 trials, risk ratio 1.0, 95% confidence interval 0.6 to 1.8, I^2^ = 0%, moderate certainty). Certainty of evidence for other outcomes ranged from very low to low, including for thromboembolic events, with only one event reported in the control arm of one trial.

**Conclusions:**

Tranexamic acid appears to reduce menstrual blood loss, but its impact on anemia is unclear. High-quality randomized trials of TXA for menstruating women with anemia are needed.

## Introduction

Globally, about half a billion women of reproductive age have anemia, with the highest prevalence observed in Western Africa (42%) and Southern Asia (49%) [1]. In 2000, the World Health Organization (WHO) set a target to halve anemia prevalence by 2025, yet progress has been minimal. The prevalence of anemia among pregnant women was 40% in 2000 and 36% in 2023. Among non-pregnant women, the prevalence was 31% in 2000 and 30% in 2023 [1]. Recent modelling suggests that the United Nations Sustainable Development Goal target to halve anemia by 2030 [2] is unachievable with current interventions alone [3]. New strategies are therefore needed.

Anemia in non-pregnant women of reproductive age (15–49 years) is defined as hemoglobin (Hb) <120 g/L [4], and iron deficiency is believed to be the most common cause. Symptoms such as fatigue, breathlessness, and cognitive impairment reduce productivity and increase healthcare costs. Anemia is particularly dangerous during pregnancy, as it increases maternal and neonatal mortality and can irreversibly affect the baby’s brain development as early as the first trimester. Addressing anemia before conception is therefore essential [5–8].

Global efforts to reduce the prevalence of anemia have focused on the provision of iron and vitamin supplements, but these may be insufficient. Side effects often reduce compliance [9,10], and menstrual bleeding as a cause of anemia has been overlooked [11–14]. Iron supplementation alone may not improve anemia if iron is continually lost through heavy menstrual bleeding (HMB) [11–13,15]. Moreover, bleeding not only causes anemia, but anemia can also worsen bleeding through various biological mechanisms [7,16], including increased blood flow and impaired clotting [17–19]. Additionally, iron deficiency may increase the risk of HMB [20]. Anemic women are therefore likely to experience heavier periods than if they were not anemic. This potential ‘vicious cycle’ between anemia and heavier menstrual bleeding could be interrupted by reducing menstrual blood loss (MBL).

The Lancet Haematology Commission recently recommended investigating therapies for HMB [21]. HMB prevalence ranges from 8 to 78%, depending on the populations and definitions [22–24]. Historically, HMB (or menorrhagia) was defined by MBL exceeding 80 mL per cycle [25]. This definition has recently been replaced by a more holistic, patient-centered one: “excessive menstrual blood loss which interferes with a woman’s physical, social, emotional and/or material quality of life [26]”. Even without anemia, HMB can affect mental health, quality of life, productivity, and healthcare costs [27–31].

Despite its impact, HMB is often underdiagnosed and undertreated or managed by hysterectomy rather than less invasive medical options. The most important medical option that preserves fertility is believed to be tranexamic acid (TXA) [13,30,32,33].

TXA is an inexpensive non-hormonal antifibrinolytic agent used for decades to treat HMB, but its potential for anemia control has not been explored. TXA blocks lysine-binding sites on plasminogen, preventing fibrin breakdown and stabilizing clots [34]. Local fibrinolytic activity is increased in HMB and can be reduced by oral TXA. Fibrinolysis may also play a role in other types of abnormal uterine bleeding (AUB) [35–37].

Literature reviews conducted over ten years ago [38,39], and a more recent systematic review (search conducted in 2017) [32] showed TXA may reduce MBL by up to 50%. However, none of these reviews examined its effect on Hb or anemia. They also excluded “pathological causes of HMB”, “iatrogenic causes of HMB” and irregular menstrual bleeding [32]. Abnormal uterine bleeding (AUB), which includes HMB, is classified by the International Federation of Gynecology and Obstetrics (FIGO) under nongestational (non-pregnant) AUB [40], and may result from polyps (AUB-P), adenomyosis (AUB-A), leiomyoma (AUB-L), endometrial malignancy (AUB-M), inherited coagulopathies (AUB-C), ovulatory disorders (AUB-O), endometrial dysfunction (AUB-E), or iatrogenic causes (AUB-I), such as anticoagulants or intrauterine devices (IUDs). Regardless of the cause, HMB is frequently associated with iron deficiency and anemia [41]. Irregular bleeding linked to hormonal contraception (AUB-I) may also increase the risk of anemia. This may occur directly through menstrual blood loss or indirectly when unscheduled bleeding prompts the discontinuation of progestins prescribed for contraception and/or the treatment of AUB. Examining TXA’s effect on Hb across all AUB subtypes is therefore important.

## Objective

To assess the effects of TXA on anemia, menstrual health and wellbeing in women of reproductive age.

## Methods

We conducted a systematic review and meta-analysis following Cochrane and PRISMA guidelines [42,43] (S1 File). We specified the methods in advance and registered the review on PROSPERO (CRD42021229181).

### Eligibility criteria

We included randomized parallel-group and crossover trials that assessed the effects of TXA compared with placebo or standard care for reducing uterine bleeding in women of reproductive age. Eligible participants included women with normal menstrual bleeding or any type of non-pregnant AUB [40]. We excluded trials involving women with uterine bleeding attributed to pregnancy, childbirth, or surgery.

Eligible trials reported on at least one of the outcomes specified in our PROSPERO registration. We defined the hemoglobin (Hb) concentration as the primary outcome. In this review, this included anemia, as defined by study authors using Hb thresholds. Secondary outcomes were estimated menstrual blood loss, quality of life (QoL), thromboembolic events (TEE), and other adverse events (nausea and vomiting).

### Search strategy

We developed a comprehensive search strategy around three key concepts: menstruation/HMB, TXA and RCTs. We searched six electronic databases and trial registries (MEDLINE, Embase, CENTRAL, Web of Science, ClinicalTrials.gov, WHO ICTRP) from inception to 30 August 2025, without language restrictions to identify relevant published, unpublished, completed, or ongoing trials. Full search terms are provided in S2 File. We also searched the reference lists of seven related reviews (S2 File).

### Study selection

After removing duplicates in Endnote X9, two reviewers independently screened all titles/abstracts and potentially relevant full-text articles for eligibility. Discrepancies were resolved by discussion.

### Data extraction and study appraisal

We developed a data extraction tool in Excel. One reviewer extracted data, and a second reviewer independently cross-checked the entries after reading the full articles. Where necessary, we used translation software and sought assistance from native speakers to assess eligibility and extract data from non-English language articles. We used WebPlotDigitizer version 5 to read data from charts where needed. Where primary outcome data were missing, we contacted study authors, making up to two email attempts.

Two reviewers independently assessed the risk of bias (ROB) for each study using an adapted version of the Cochrane ROB tool [44]. We used GRADEpro|GDT software to assess the certainty of evidence across studies [45,46]; one reviewer conducted the assessments, and a second reviewer cross-checked them. We resolved disagreements by discussion.

### Data synthesis and analysis

After summarizing studies according to outcomes and population type, we conducted meta-analyses where sufficient data were available from at least two comparable studies. We used Review Manager 5.3 [44] for computing effect sizes and conducting meta-analyses, following Cochrane guidance [42]. Forest plots were generated in R 4.5.

First, we calculated risk ratios (RR) with 95% confidence intervals (CI) for dichotomous data. For continuous data, we calculated mean differences and 95% CI using the means and standard deviations extracted from each trial. For trials reporting medians and interquartile ranges, we estimated the mean and standard deviation using the method described in Wan et al. (2014 [47]). For trials involving multiple groups with different dose regimens, we combined eligible groups to create a single pairwise comparison. Where necessary (for subjective MBL outcome), we converted summary measures into standardized mean differences to make them comparable across different studies.

We then conducted meta-analyses using the fixed-effect model, as pre-specified in our PROSPERO registration, and generated summary estimates. We pooled studies only where at least two studies were sufficiently comparable in terms of populations, interventions and outcomes. Where relevant, we pooled results separately for clinically distinct population groups and for objective versus subjective outcomes. We conducted random-effects sensitivity analyses to assess the robustness of the findings to the choice of meta-analysis model. Where pooling of data was not possible, we presented results in narrative form or in summary tables.

We analyzed trial data on an intention-to-treat basis where the necessary data were available. We compared outcome measures at follow-up between intervention and control groups. For studies reporting results for multiple time points, we selected the last time point available, unless it was a time point where TXA was no longer present at a therapeutic dose. For studies on unscheduled bleeding after contraceptive initiation, we selected the earliest available time point, as bleeding is often most intense during the initial months [48]. Where endpoint data were not available, we used change-from-baseline data. For safety outcomes, we used data up to the follow-up period specified by each individual study.

For crossover trials, we included data on the menstrual blood loss outcomes (amount and duration) and adverse events from both phases following the methods described by Elbourne et al. (2002 [49]). We considered carry-over effects unlikely for menstrual bleeding outcomes measured separately during each treatment period because TXA has a short-lived pharmacological effect relative to the interval between menstrual cycles. For objectively measured menstrual blood loss, we calculated paired effect estimates and standard errors from participant-level data, thereby accounting for within-participant correlation.

For Hb and anemia outcomes, where carry-over effects are likely, we had planned to analyze only first-phase data; however, these were unavailable. We therefore did not include Hb or anemia data from crossover trials.

For our primary outcome, we planned to use mean differences that are adjusted for baseline Hb if available or consider baseline Hb differences when interpreting the results. We also considered any pre-specified clinically important differences identified by the study authors, if reported.

We assessed statistical heterogeneity by visual inspection of the forest plot and by calculating the I^2^ statistic. Where substantial heterogeneity (I^2^ ≥ 50%) [42] was present, we conducted exploratory sensitivity analyses restricted to parallel trials (excluding crossover trials). We had planned to explore potential sources of heterogeneity through subgroup analyses comparing anemic and non-anemic participants; however, this was not possible due to insufficient data.

We intended to investigate reporting bias with funnel plots if at least 10 trials contributed data to an analysis; however, too few comparable trials were available.

## Results

### Study selection

From a total of 1257 identified records, we included 22 articles reporting on 17 trials (Fig 1 and S3 File). Among these, 15 trials with a total of 1080 participants were included in meta-analyses. We also identified 4 ongoing trials (S4 File).

**Fig 1:**
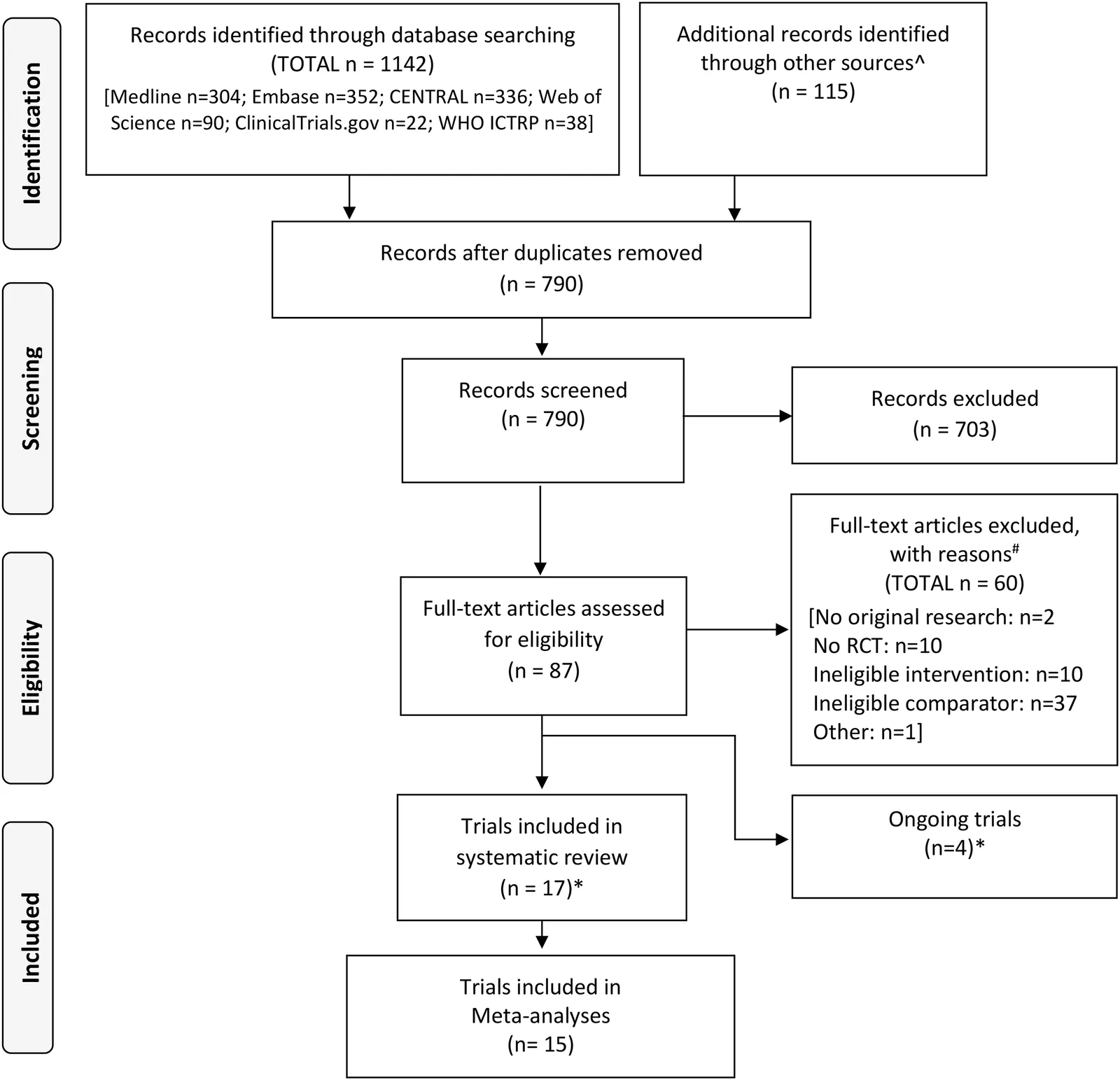
Flow chart of the study selection process. ^ Reference lists of seven systematic reviews searched; # Details and list of excluded articles with reasons in Supplementary file 3. * n = 17 included trials reported in n = 22 articles; n = 4 ongoing trials reported in n = 5 articles (Supplementary file 4). Flow chart adapted from: Moher D, Liberati A, Tetzlaff J, Altman DG, the PRISMA group (2009). Preferred reporting items for systematic reviews and meta-analyses: the PRISMA statement. PLoS Med 6 (7): e1000097. RCT, randomised controlled trial.

### Study characteristics

Table 1 summarises the main characteristics, interventions and comparators of the included trials. S5 File provides further details.

**Table 1:** Characteristics of included studies.

Study (country)	Design	Participants, Total number, N	TXA intervention*	Comparator*	Outcomes included in review
Adawiyah 2020 (Indonesia)	RCT, 1:1 parallel	Women with abnormal uterine bleeding secondary to contraceptive injection (DMPA); N = 44	TXA 250 mg 4x/day + OCP once daily for 28 days; single course	Matched placebo + OCP once daily for 28 days	Bleeding duration; side effects
Callender 1970 (UK)	Crossover trial	Patients with HMB or iron deficiency anaemia presumably due to HMB; N = 16	TXA 1 g 4x/day on days 1–4 of menstruation; 3 cycles	Matched placebo; 3 cycles	MBL, obj. (OTBC method); number of pads used; bleeding duration; side effects
Drosdal 1983 (Norway)	Crossover trial	Women with HMB and/or iron deficiency anaemia related to HMB; N = 44 or 37 (unclear)	TXA 1 g 3x/day on days 2–5 of menstruation; 3 cycles	Matched placebo; 3 cycles	Hb; MBL, subj. (visual analogue scale); number of tampons/pads used; side effects
Edlund 1995 (Sweden)	RCT, 2:2:1, parallel	Women with HMB (>80ml MBL/cycle), regular cycles; N = 68	TXA prodrug (Kabi 2161) on days 1–5 of menstr.; 3 cycles: − 1.2 g/day (2 tablets 2x/day) − 0.6 g/day (1 tablet 4x/day)	Placebo on days 1–5 of menstruation for 3 cycles: 2 tablets 4x/day	MBL, obj. (modified AH method); MBL, subj. (perceived change); Number of tampons/ pads used; bleeding duration; (Hb not reported; side effects not reported separately by group)
Freeman 2011 (USA)	RCT, ~2:2:1, parallel	Women with HMB (≥80 ml/cycle), regular cycles; N = 294	TXA 0.65 g or 1.3 g 3x/day for up to 5 days of menstruation; 3 cycles	Placebo for up to 5 days of menstruation; 3 cycles	MBL, obj. (AH method); Quality of Life (physical, social/leisure activities); side effects
Hamulyák 2024 (Netherlands)	RCT, 1:1:1, parallel	Women with FXa inhibitor–associated HMB (PBAC score ≥150); N = 16	Factor Xa inhibitor + TXA 1 g 3x/day on days 1–4 of menstruation; 3 cycles	Factor Xa inhibitor alone; 3 cycles	MBL, subj. (PBAC); side effects; (Hb and other secondary outcomes not reported)
Lin 2007 (China)	RCT, 1:1:1, parallel	Women with newly inserted Copper IUD; N = 175 (N = 64 for obj. outcome)	TXA 1 g or 500 mg 2x/day on days 1–5 of menstruation; 3 cycles after IUD insertion	Placebo on days 1–5 of menstruation; 3 cycles after IUD insertion	MBL, obj. (AH method); MBL, subj. (PBAC)
Lukes 2010 (USA)	RCT, ~2:1 parallel	Women with HMB (≥80 ml/cycle), regular cycles; N = 187;	TXA 1.3 g (2 tablets) 3x/day from menstruation onset for up to 5 days/cycle; 6 cycles	Matched placebo; 6 cycles	Hb; MBL, obj. (AH method); MBL, subj. (perceived MBL); Quality of Life (physical, social/leisure activities); side effects
Nilsson 1967 (Sweden)	Crossover trial	Women with HMB, ovulatory cycle; N = 36	TXA 0.25 g or 0.5 g 6x/day on days 1–4 of menstr.; 1 cycle/ dose. After first 8 participants, doses increased to 0.5 g and 1 g	Placebo on days 1–4 of menstruation	MBL, obj. (AH method); side effects;
Petersen 1983 (Denmark)	Crossover trial	Women with HMB (>80 ml), regular cycle; N = 17	TXA 1.5 g (3 tablets) 3x/day on days 1–4 of menstruation; 1 cycle	Placebo; 1 cycle	MBL, obj. (AH method); bleeding duration; absence from work; side effects
Phupong 2006 (Thailand)	RCT, 1:1 parallel	Women with irregular uterine bleeding secondary to contraceptive implant (Norplant) use; N = 68	TXA 500 mg (2 capsules) 4x/day for 5 days; single course	Matched placebo; single course	Cessation of bleeding after 5-day treatment initiation; side effects
Rivera 1984 (Mexico or USA)	RCT, 1:1 parallel	Women having contraceptive IUD inserted; N = 18 (outcomes at 1 month)	IUD releasing TXA locally	Otherwise identical modified IUD with empty reservoirs	MBL, obj. (AH method); bleeding duration; side effects
Senthong 2009 (Thailand)	RCT, 1:1 parallel	Women with irregular uterine bleeding secondary to contraceptive injection (DMPA); N = 99	TXA 250 mg 4x/day for 5 days; single course	Placebo; single course	Cessation of bleeding after 5-day treatment initiation; side effects
Sordal 2013 (Denmark, Ireland, & Norway)	RCT, 1:1:1 parallel	Healthy women (without HMB) having a hormonal contraceptive IUD (LNG-IUS) inserted; N = 184	TXA 500 mg 3x/day during each bleeding/spotting episode for 90 days after LNG-IUS insertion	Placebo during each bleeding/spotting episode for 90 days after LNG-IUS insertion	Number of bleeding/ spotting days during the 90-day period post-IUD insertion; side effects
Vermylen 1968 (Belgium)	Crossover trial	Women with HMB (‘essential menorrhagia’); N = 16	TXA 500 mg 6x/day from menstruation onset until bleeding stopped; 3 cycles	Placebo from menstruation onset until bleeding stopped; 3 cycles	Mean menstrual Hb loss, obj. (Perdometer method); bleeding duration; side effects
Weström 1970 (Sweden)	RCT, 1:1 parallel	Women having contraceptive IUD inserted, N = 65 (N = 55 for Hb outcome)	TXA 1.5 g 3x/day for 5 days; if needed, 1 g 2x/day for a further 5 days; 3 cycles	Placebo; 3 cycles	Hb; MBL, obj. (AH method); bleeding duration; side effects
Ylikorkala 1983 (Finland)	Crossover trial	Women with contraceptive IUD and HMB; N = 19	TXA 1.5 g 3x/day on days 1–5 of menstruation; 2 cycles	Placebo; 1 cycle	Hb; MBL, obj. (AH method); bleeding duration; side effects; ‘restricted activity’; subj. treatment effect

* Interventions and comparators relevant to this review only; ^ Total number N across all treatment arms included in analysis for main outcome (often differed from numbers randomised, e.g., in case of modified ITT-analysis); AH method, alkaline hematin method to determine menstrual blood loss ^25^; DMPA, Depo-medroxy progesterone acetate (a long-acting progestin); g, gram; Hb, haemoglobin; HMB, heavy menstrual bleeding; ITT, intention-to-treat; IUD, intrauterine device; LNG-IUS, levonorgestrel-releasing intrauterine system (long-acting progestin). MBL, menstrual blood loss; Norplant, six-capsule levonorgestrel subdermal implant (a long-acting progestine); obj., objective assessment method; OTBC method, Oxford total body counter method^57^; parallel, parallel-group trial; PBAC, pictorial blood loss assessment chart; Perdometer method, developed by Rustad 1963^58^; RCT, randomized controlled trial; subj., subjective assessment method; TXA, tranexamic acid; vs, versus

The included trials were published between 1967 and 2024. Eleven used a parallel design and six a crossover design. Most trials were conducted in high-income countries, and sample sizes ranged from 12 to 294. Nine trials included women with suspected or diagnosed HMB [50–58]. The remaining eight trials involved women with AUB related to IUDs (inert, copper and hormone-releasing) or hormonal contraception (progestin-only implants and injections) [59–66]. One trial compared a TXA-releasing IUD with an inert IUD [61]; all others compared oral TXA with placebo, except for one open-label trial that compared oral TXA with no TXA [54]. TXA regimens varied, with tablets taken two to six times daily. Total daily dosages ranged from 1.5 to 6.0 g, usually for the first four or five days of bleeding or the entire bleeding duration. In one trial treatment began at day 2 of menstruation [52].

Outcomes were assessed after 1–6 menstrual cycles. Six trials [51,52,54,55,64,65] measured venous Hb during follow-up but either did not report results or provided insufficient detail, as discussed below. Three trials [53,55,64] reported limited data on anemia. Other outcomes relevant to this review included objectively measured menstrual blood loss (MBL, n = 11 trials), subjectively measured MBL (n = 4), bleeding duration (n = 8), bleeding cessation (n = 2), number of pads/tampons used (n = 3), QoL (n = 3) and side effects (all but one trial [66]). S5 File provides details.

### Risk of bias and certainty of evidence

Most trials lacked detail on key ROB domains. Only two [60,63] of 17 trials described the method of random sequence allocation and only four [54,55,60,63] reported adequate allocation concealment. Almost all trials were described as double-blind, except for one open-label trial [54] and one trial [61] where blinding of outcome assessment was unclear. ROB regarding selective reporting was ‘unclear’ for 14 trials and ‘high risk’ for two trials [52,54]. Only four trials [53–55,63] had been prospectively registered. ROB regarding incomplete outcome data varied (details in S6 File).

Given the unclear ROB regarding randomization, we explored randomization success through meta-analyses of selected baseline variables [67,68]. Due to insufficient baseline Hb data, we selected age and baseline MBL as prognostic covariates. (The premise of this analysis was that if the trials were properly randomized, there would be no heterogeneity, i.e., I^2^ = 0% and any difference in baseline variables would be minimal and the result of random error.) There was no heterogeneity for either variable (I^2^ = 0%). However, women allocated to the TXA group had a slightly higher baseline MBL than those in the control group (p = 0.02, S7 File).

The certainty of evidence was very low or low for most outcomes, but moderate for objective MBL and QoL in women with HMB and potential nausea/vomiting side effects (details in S8 File).

### Synthesis of results

Below, we summarize results by outcome and population; individual trial findings and reasons for data not included in meta-analyses are presented in S9 File.

### Primary outcomes

Meta-analysis of primary outcomes was not possible, because trial reports lacked sufficient information. We sought additional data or clarification for seven trials. Authors of three trials responded; one provided additional hemoglobin data, although these remained insufficient for effect estimation, while authors of two trials could no longer access the required data or records. For the remaining four trials, we received no response or could not identify current contact details (see S9 File). Data on hemoglobin concentration were insufficient, and certainty of evidence for the anemia outcome was very low, with only one small trial among women with IUDs contributing data [64]. In that trial, 0 of 27 participants in the TXA group versus 3 of 28 in the placebo group had Hb < 10.5 g/dL after three cycles (RR 0.15, 95% CI 0.01 to 2.74).

### Secondary outcomes

Meta-analyses of key secondary outcomes are presented in Figs 2–4 with additional forest plots in S10 File. Nine trials (five parallel and four crossover trials) contributed data to the meta-analysis of objectively measured MBL (mL). Data from another trial [58] could not be included, as it reported menstrual hemoglobin loss in grams (S9 File). TXA reduced mean blood loss by 47 mL among women with HMB (moderate certainty) and 42 mL among women with IUDs (low certainty; see Fig 2, S8 and S9 Files). Heterogeneity was low for the HMB group (I^2^ = 13%) but substantial for the IUD group (I^2^ = 64%). In a random‑effects sensitivity analysis, pooled effects were similar to fixed‑effect estimates (HMB: −48 mL; IUD: −45 mL), and heterogeneity in the IUD group remained substantial. Excluding crossover trials reduced heterogeneity in the IUD group to I^2^ = 0% and slightly attenuated the effect estimate to a 36 mL reduction at about one month post-enrolment in this population (S10 File).

**Fig 2:**
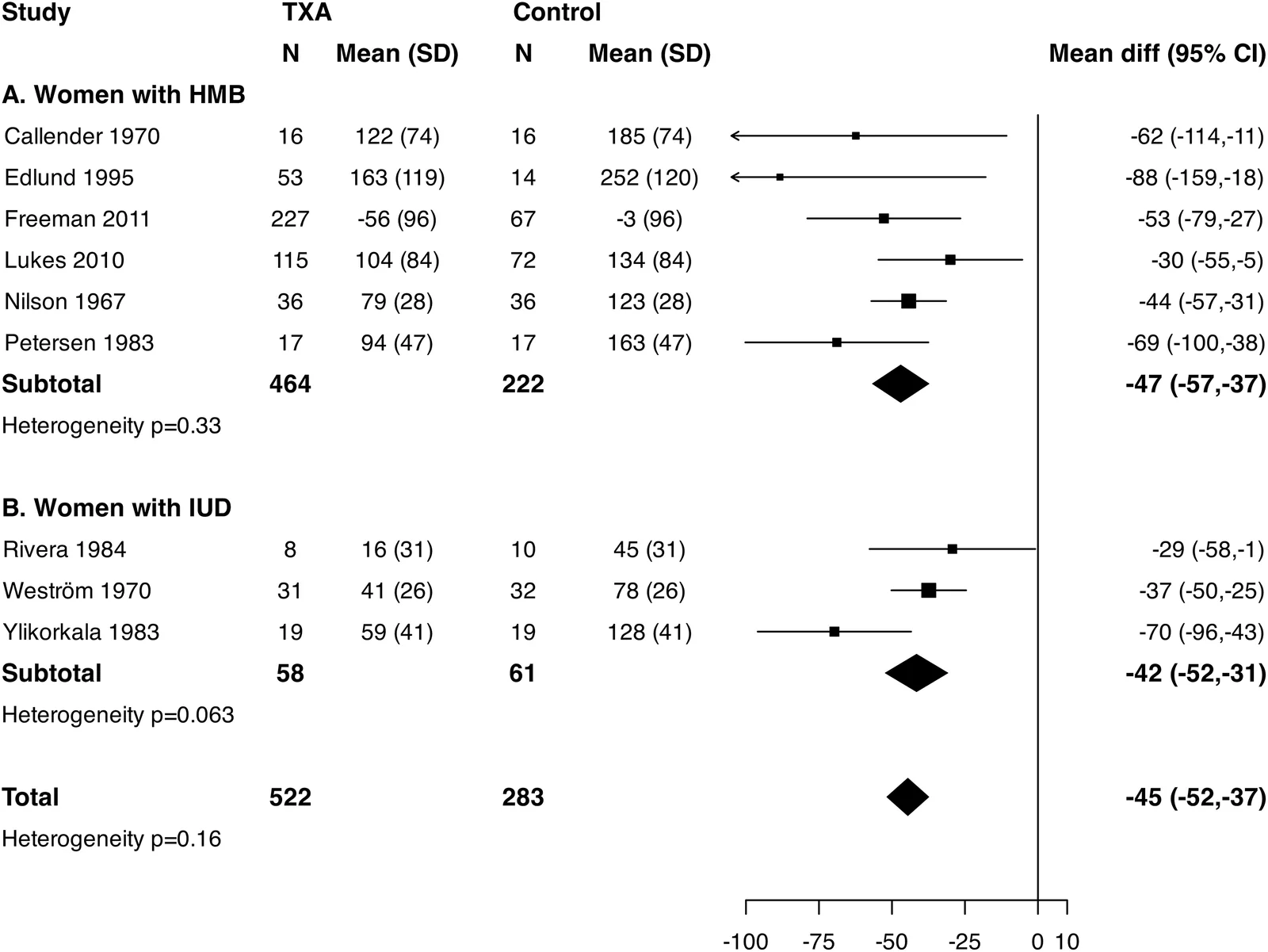
Effect of tranexamic acid on objective menstrual blood loss (mL). Forest plot showing mean differences in menstrual blood loss between tranexamic acid and control groups. Negative values (to the left) indicate reduced menstrual blood loss with tranexamic acid. Group A includes women with heavy menstrual bleeding. Group B includes women with inert intrauterine devices (IUDs; Rivera 1984, Westroem 1970) and copper IUDs (Ylikorkala 1983).

**Fig 3:**
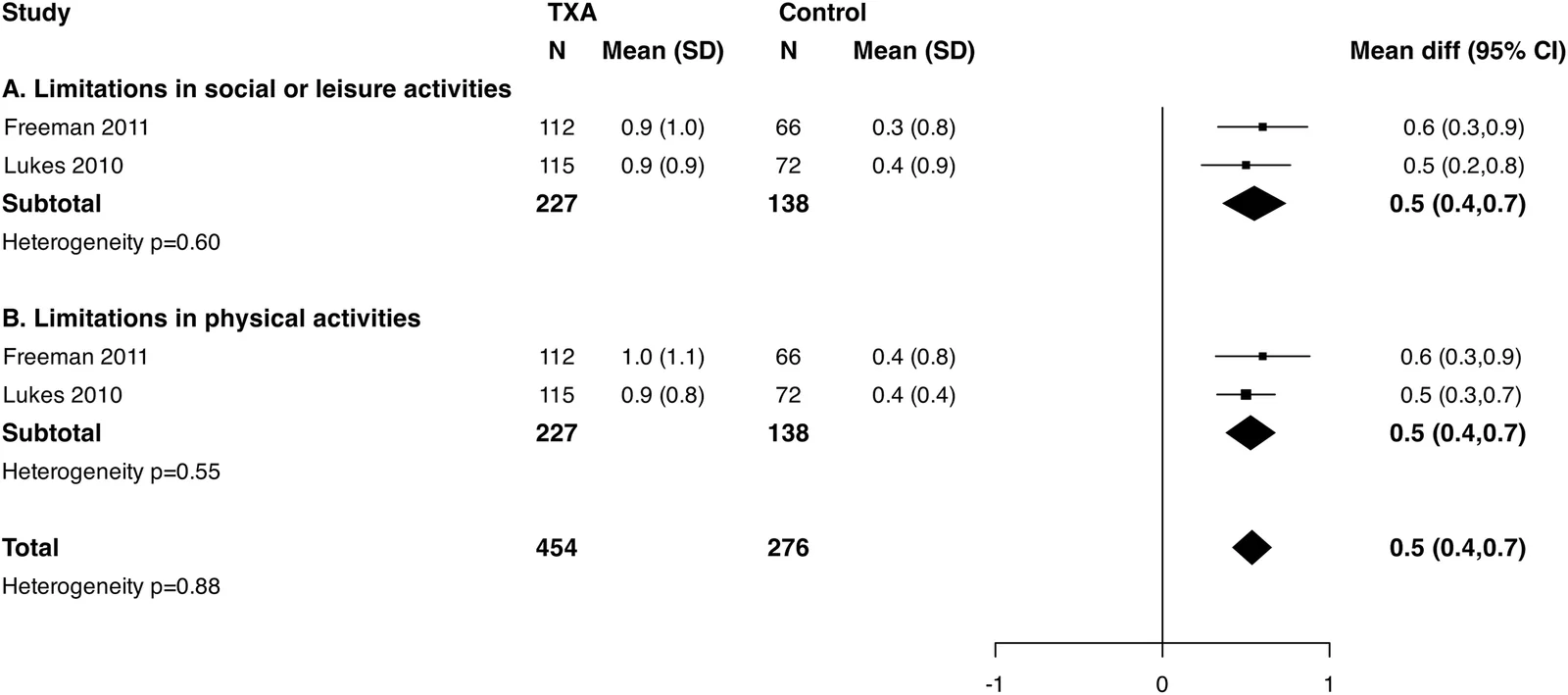
Effect of tranexamic acid on quality of life. Forest plot showing mean differences in quality-of-life scores between tranexamic acid and control groups for limitation of social or leisure activities (A), and physical activities (B) associated with heavy menstrual bleeding (HMB). Positive values (to the right) indicate improvements with tranexamic acid.

**Fig 4:**
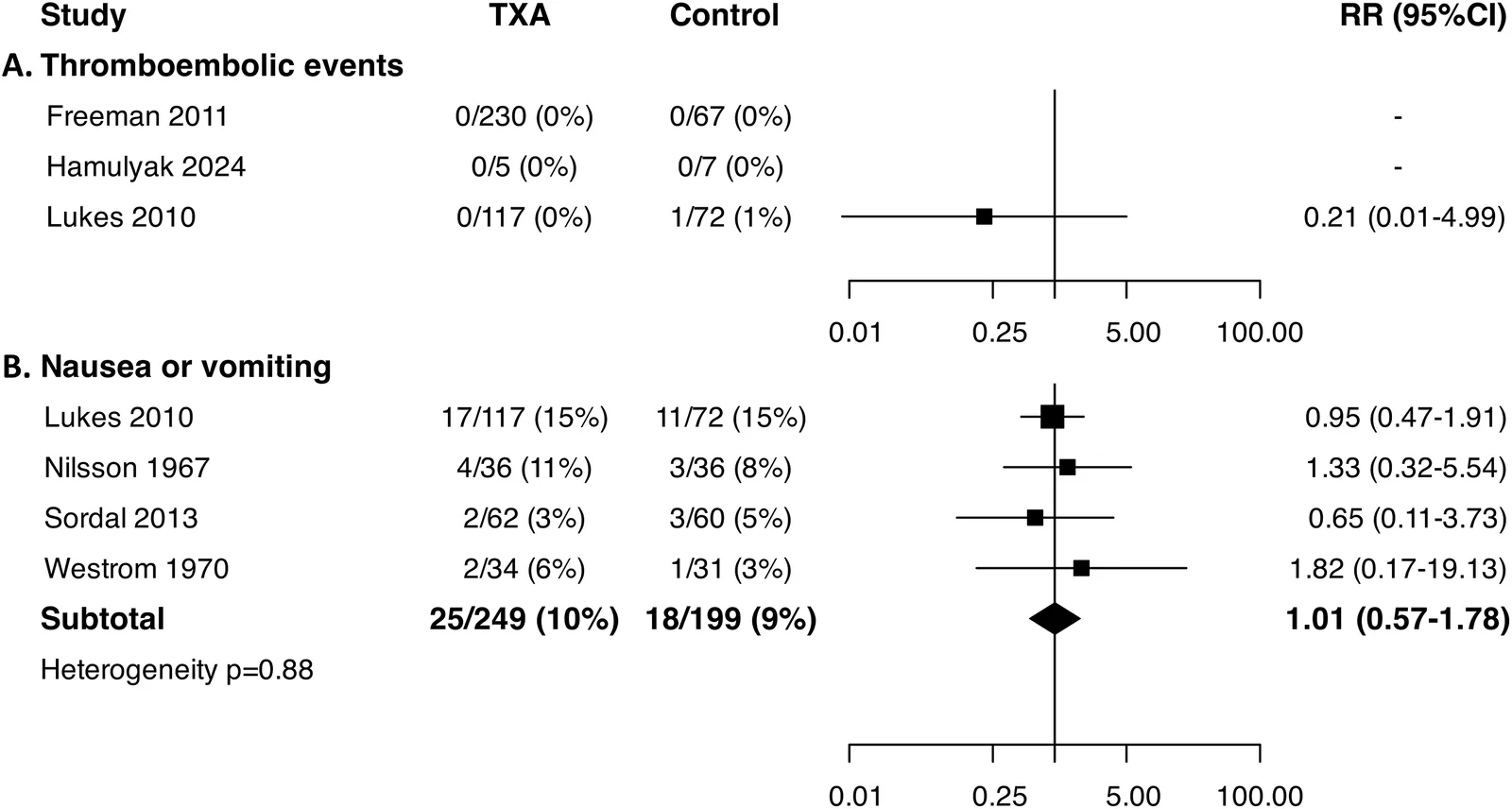
Forest plots of trials reporting pre-specified adverse events. Forest plots showing risk ratios for potential adverse events comparing tranexamic acid and control groups, including cumulative thromboembolic events (A) and cases of nausea and/or vomiting (B) at study endpoint.

Meta-analyses also showed benefits of TXA treatment for subjectively measured MBL (two trials, SMD −0.95, 95% CI −1.25 to −0.65, I^2^ = 0%, low certainty) and for the number of pads/tampons used (two trials, MD −5.4, 95% CI −7.9 to −2.8, I^2^ = 41%, low certainty). Certainty for a beneficial effect on bleeding cessation within seven days among women with AUB secondary to hormonal contraception was very low. For details and other bleeding-related outcomes see S8-S10 Files.

Two trials [53,55] among women with HMB reported the impact on QoL using the Menorrhagia Impact Questionnaire [69]. Fig 3 shows that tranexamic acid improved scores for both “social or leisure activities” and “physical activities” (moderate certainty).

Four trials (three parallel and one crossover trial) reported on potential side effects of nausea/vomiting, with a similar risk between groups (moderate certainty; Fig 4). Certainty of evidence for TEE from three trials was very low with only one event reported in a control arm. None of the other trials mentioned any TEE. Additional side effects not pre-specified in our PROSPERO registration were reported across trials and appeared broadly similar between treatment and control groups (see S11 File).

## Discussion

### Principal findings

Our systematic review revealed a major evidence gap. None of the 17 identified trials focused on TXA for anemia, and none provided suitable data on hemoglobin concentration. Certainty of evidence for the anemia outcome was very low, with only one small trial contributing data. TXA reduces MBL with moderate certainty, and there is some evidence that it improves QoL, although this outcome was rarely measured. There was no evidence for an increased risk of side effects with TXA use.

### Comparison with existing literature

This is the first systematic review on TXA for anemia in women of reproductive age; therefore, our primary findings cannot be compared with previous reviews.

Our findings regarding secondary outcomes corroborate those from previous related reviews, which focused on TXA for HMB rather than anemia. One review, based on four trials, reported a mean reduction of MBL by 53 mL per cycle (95% CI −63 to −44; moderate certainty) [32]. Our estimate of a 47 mL reduction based on six trials is consistent with these findings.

Although reducing menstrual blood loss is a plausible pathway through which TXA could reduce anemia risk, the magnitude of blood loss reduction required to improve hemoglobin concentration remains uncertain. The time needed for any such improvement would likely depend on multiple factors, including baseline Hb concentration, iron status, ongoing blood loss, and the underlying causes of anemia. Future trials should therefore measure hemoglobin and iron status alongside menstrual blood loss and include sufficient follow-up to assess whether reductions in bleeding translate into clinically meaningful improvements in anemia.

Whether an objective MBL reduction of about 40–50 mL can substantially decrease anemia within an acceptable time frame cannot be determined from the available data. However, this reduction appears to be meaningful for patients based on a prior study, which found that reductions exceeding 36 mL per cycle were considered meaningful for women with HMB [70]. Further research is needed to confirm whether this threshold applies across different populations and AUB types [40]. Patient perceptions may also depend on bleeding patterns. For example, ‘flooding’ (sudden blood loss that exceeds the saturation of menstrual products), which often occurs on the second or third day of menstruation among women with HMB, has a particular negative impact on daily life [71,72]. A reduction of flooding may therefore matter more to women than a reduction of the bleeding duration or average MBL. A post-hoc analysis of two trials [53,55] showed that the highest daily MBL occurred on days 2–3 and that reductions in daily MBL were associated with improved QoL [73].

Our review findings of improved QoL were based on these two trials [53,55] and are consistent with earlier research that pooled data from the same trials [32,73]. Both were conducted in the USA by collaborating research teams supported by the same pharmaceutical funder [53,55]. Evidence from other settings remains limited, as most RCTs have focused on MBL rather than broader quality-of-life measures. A relatively large German study that concluded that TXA improves QoL in women with HMB was an ‘open, uncontrolled usage study’ with high risk of bias [74].

Finally, TXA tolerability and safety are crucial factors in determining whether TXA is suitable for preventing and treating anemia and/or various forms of AUB, including HMB. Our safety findings align with a previous systematic review on TXA for HMB, which found no clear evidence of differences in rates of specific adverse events, although confidence intervals were wide [32]. In contrast, our confidence interval for nausea/vomiting was relatively narrow, and we conclude with moderate certainty that oral TXA does not increase this risk. We found no evidence of increased TEE risk, but only two trials assessed TEE and neither was powered for rare events. There are no systematic reviews on the risk of TEE in menstruating women receiving oral TXA treatment. However, our findings align with case-control studies on oral TXA treatment [75,76] and large meta-analyses of intravenous TXA [77,78]. Similarly, a meta-analysis including oral and intravenous TXA found no evidence of increased TEE risk in non-surgical patients [79].

### Strengths and limitations

To our knowledge this is the first systematic review on tranexamic acid for anemia among menstruating women. We followed rigorous procedures and standards for evidence grading and reporting [42,43,45]. Our eligibility criteria ensured that trials on different AUB types were included.

The main limitations are the lack of data and the high or unclear risk of bias in the included trials. Six trials measured Hb during follow-up, but the reported data were insufficient for meta-analysis because of non-reporting of Hb results, inadequate summary statistics, lack of treatment-group or first-period data, concerns about carry-over effects, very small treatment groups, or uncertainty regarding concomitant iron treatment. Despite attempts to contact trial authors, we did not obtain sufficient data for meta-analysis of our primary outcomes. Most trials were conducted more than ten years ago, were not prospectively registered, and did not follow standardized reporting guidelines. It was therefore often impossible to assess bias related to incomplete outcome reporting. The number of trials was also too low to create funnel plots. We therefore cannot discount reporting bias, including publication bias, despite a comprehensive search. Incomplete reporting also meant that bias relating to the randomization process was unclear for most studies. These methodological limitations reduce confidence in the effect estimates, particularly for subjective outcomes, where inadequate reporting of randomization methods and allocation concealment may have increased the risk of biased treatment effects.

Our additional analysis to explore the randomization process showed low heterogeneity of baseline variables. However, we found that baseline MBL favored the control arm. Although the difference was small, we did not expect baseline MBL to consistently favor one randomized group over the other. This finding might have been due to chance rather than bias, as most trials were small and did not report power calculations. It is possible, however, that this baseline imbalance attenuated the observed favorable effect of TXA; the actual mean difference in MBL may be slightly higher than 47 mL, as most trials did not adjust for baseline imbalances.

The number of studies was also too low to conduct pre-specified subgroup analyses for participants with and without anemia at baseline.

In addition, two older trials evaluated women using inert IUDs and one trial included women using the Norplant contraceptive implant. As these technologies are no longer commonly used, findings from these subgroups may have limited applicability to contemporary populations. More broadly, most trials were conducted more than ten years ago and in high-income countries, none specifically recruited women with anemia, and none was conducted in sub-Saharan Africa. Differences in populations, clinical contexts and contraceptive technologies may therefore limit the generalizability of the findings.

A further limitation regarding our safety findings is that none of the included trials was powered to detect rare adverse events. Additionally, most trials had follow-up periods of three months or less, which did not allow assessment of long-term safety.

## Conclusions and implications

Tranexamic acid appears to reduce menstrual blood loss, but its effect on anemia remains unclear. The available evidence therefore supports the use of TXA for reducing menstrual blood loss, but provides little direct evidence regarding improvement in Hb concentration or anemia. Future trials should determine the magnitude of blood loss reduction and duration of treatment required to achieve clinically meaningful improvements in Hb concentration and anemia outcomes across different populations. None of the included trials focused on anemia as a primary outcome, and most had high or unclear risk of bias. High-quality randomized trials of TXA for menstruating women with anemia are needed. Research should extend beyond high-income countries to regions with the highest anemia prevalence, including sub-Saharan Africa and South Asia, to determine the effectiveness, safety, and acceptability in these settings. Our review identified four ongoing trials, only one of which – the WOMAN-3 trial in Nigeria, Tanzania, and Pakistan [80] – specifically examines TXA’s impact on anemia. Trials in high-income countries and related cost-effectiveness studies are equally important.

TXA is an established, low-cost, and well-tolerated treatment for HMB. Future research should determine whether it can become a valuable component of global anemia control. Addressing anemia before pregnancy could reduce adverse maternal and neonatal outcomes and improve wellbeing across the reproductive life course [8].

## Supporting information

S1 File PRISMA checklist. (PDF)

S2 File Full search strategy and list of relevant systematic reviews. (PDF)

S3 File List of excluded articles with reasons. (PDF)

S4 File List of ongoing trials with main characteristics. (PDF)

S5 File Detailed characteristics of included trials. (PDF)

S6 File Risk of bias assessments of included trials. (PDF)

S7 File Differences in baseline variables. (PDF)

S8 File GRADE certainty of evidence assessments. (PDF)

S9 File Results of individual trials. (PDF)

S10 File Additional forest plots. (PDF)

S11 File List of other side effects. (PDF)
