# Supplementary material for: Tranexamic acid for anemia in women of reproductive age: A systematic review of randomized trials
Source: PLoS One. 2026 Nov 6;21(11):e0362488. doi: 10.1371/journal.pone.0362488 (PMC13743017; doi:10.1371/journal.pone.0362488)
Supplement: Supplementary file 2 [file pone.0362488.s002.pdf]

Supplementary File 2 – Full search strategy and list of relevant reviews

Berendes et al. (2026), PLOS ONE. DOI: 10.1371/journal.pone.0362488

A Database and trial registry search

All database and trial registry searches were run without any language or publication date restrictions on 30th August 2025.

Ovid MEDLINE

1 exp Menstruation/
2 exp Uterine Hemorrhage/
3 (menstru* or heavy period* or menses or menorrhagia).ab,ti
4 1 or 2 or 3
5 exp Tranexamic Acid/
6 (tranexamic or Cyclohexanecarboxylic Acid* or Methylamine* or amcha or amca or kabi 2161 or transamin or exacyl or amchafibrin or anvitoff or spotof or cyklokapron or ugurol or aminomethylcyclohexanecarbonic acid or aminomethylcyclohexanecarboxylic acid or AMCHA or amchafibrin or amikapron or aminomethyl cyclohexane carboxylic acid or aminomethyl cyclohexanecarboxylic acid or aminomethylcyclohexane carbonic acid or aminomethylcyclohexane carboxylic acid or aminomethylcyclohexanecarbonic acid or aminomethylcyclohexanecarboxylic acid or aminomethylcyclohexanocarboxylic acid or aminomethylcyclohexanoic acid or amstat or anvitoff or cl?65336 or cl65336 or cyclocapron or cyclokapron or cyklocapron or exacyl or frenolyse or hexacapron or hexakapron or tranex or TXA or lysteda).ab,ti.
7 5 or 6
8 randomized controlled trial.pt.
9 controlled clinical trial.pt.
10 randomi?ed.ti,ab.
11 placebo.ti,ab.
12 drug therapy.sh.
13 randomly.ab,ti.
14 trial.ti,ab.
15 groups.ti,ab.
16 8 or 9 or 10 or 11 or 12 or 13 or 14 or 15
17 Animals/ NOT Humans/
18 16 not 17
19 4 and 7 and 18

OVID EMBASE

1. exp menstruation/
2. exp uterus bleeding/
3. exp "menorrhagia and metrorrhagia"/
4., (menstru* or heavy period* or menses or menorrhagia).ab,ti.
5. 1 or 2 or 3 or 4
6. exp tranexamic acid/
7. (tranexamic or Cyclohexanecarboxylic Acid* or Methylamine* or amcha or amca or kabi 2161 or transamin or exacyl or amchafibrin or anvitoff or spotof or cyklokapron or ugurol or aminomethylcyclohexanecarbonic acid or aminomethylcyclohexanecarboxylic acid or AMCHA or

amchafibrin or amikapron or aminomethyl cyclohexane carboxylic acid or aminomethyl cyclohexanecarboxylic acid or aminomethylcyclohexane carbonic acid or aminomethylcyclohexane carboxylic acid or aminomethylcyclohexanecarbonic acid or aminomethylcyclohexanecarboxylic acid or aminomethylcyclohexanocarboxylic acid or aminomethylcyclohexanoic acid or amstat or anvitoff or cl?65336 or cl65336 or cyclocapron or cyclokapron or cyklocapron or exacyl or frenolyse or hexacapron or hexakapron or tranex or TXA or lysteda).ab,ti.
8. 6 or 7
9., random*.tw.
10. placebo*.mp.
11. double-blind*.tw.
12. or/9-11
13. 5 and 8 and 12

CENTRAL

#1 MeSH descriptor: [Menstruation] explode all trees

#2 MeSH descriptor: [Uterine Hemorrhage] explode all trees

#3 (menstru* or heavy period* or menses or menorrhagia):ab,ti

#4 #1 or #2 or #3

#5 MeSH descriptor: [Tranexamic Acid] explode all trees

#6 (tranexamic or Cyclohexanecarboxylic Acid* or Methylamine* or amcha or amca or kabi 2161 or transamin or exacyl or amchafibrin or anvitoff or spotof or cyklokapron or ugurol or aminomethylcyclohexanecarbonic acid or aminomethylcyclohexanecarboxylic acid or AMCHA or amchafibrin or amikapron or aminomethyl cyclohexane carboxylic acid or aminomethyl cyclohexanecarboxylic acid or aminomethylcyclohexane carbonic acid or aminomethylcyclohexane carboxylic acid or aminomethylcyclohexanecarbonic acid or aminomethylcyclohexanecarboxylic acid or aminomethylcyclohexanocarboxylic acid or aminomethylcyclohexanoic acid or amstat or anvitoff or cl?65336 or cl65336 or cyclocapron or cyclokapron or cyklocapron or exacyl or frenolyse or hexacapron or hexakapron or tranex or TXA or lysteda):ab,ti

#7 #5 or #6

#8 #4 and #7

Web Of Science

TI=((menstru* or menorrhagia or (heavy NEAR/3 period*) or menses)) OR AB=((menstru* or menorrhagia or (heavy NEAR/3 period*) or menses))

TI=(tranexamic or Cyclohexanecarboxylic Acid* or Methylamine* or amcha or amca or kabi 2161 or transamin or exacyl or amchafibrin or anvitoff or spotof or cyklokapron or ugurol or

aminomethylcyclohexanecarbonic acid or aminomethylcyclohexanecarboxylic acid or AMCHA or amchafibrin or amikapron or aminomethyl cyclohexane carboxylic acid or aminomethyl cyclohexanecarboxylic acid or aminomethylcyclohexane carbonic acid or aminomethylcyclohexane carboxylic acid or aminomethylcyclohexanecarbonic acid or aminomethylcyclohexanecarboxylic acid or aminomethylcyclohexanocarboxylic acid or aminomethylcyclohexanoic acid or amstat or anvitoff or cl?65336 or cl65336 or cyclocapron or cyclokapron or cyklocapron or exacyl or frenolyse or hexacapron or hexakapron or tranex or TXA) OR AB=(tranexamic or Cyclohexanecarboxylic Acid* or Methylamine* or amcha or amca or kabi 2161 or transamin or exacyl or amchafibrin or anvitoff or spotof or cyklokapron or ugurol or aminomethylcyclohexanecarbonic acid or aminomethylcyclohexanecarboxylic acid or AMCHA or amchafibrin or amikapron or aminomethyl cyclohexane carboxylic acid or aminomethyl cyclohexanecarboxylic acid or aminomethylcyclohexane carbonic acid or aminomethylcyclohexane carboxylic acid or aminomethylcyclohexanecarbonic acid or aminomethylcyclohexanecarboxylic acid or aminomethylcyclohexanocarboxylic acid or aminomethylcyclohexanoic acid or amstat or anvitoff or cl?65336 or cl65336 or cyclocapron or cyclokapron or cyklocapron or exacyl or frenolyse or hexacapron or hexakapron or tranex or TXA)

TI= (randomised OR randomized OR randomisation OR randomisation OR placebo* OR (random* AND (allocat* OR assign*)) OR (blind* AND (single OR double OR treble OR triple))) OR AB= (randomised OR randomized OR randomisation OR randomisation OR placebo* OR (random* AND (allocat* OR assign*)) OR (blind* AND (single OR double OR treble OR triple)))

[AND the above]

WHO ICTRP - 30/8/25

Menstru* OR menorrhagia in CONDITION
Antifibrinolytic* OR Anti-fibrinolytic* OR tranexamic OR TXA OR trans-4-aminomethylcyclohexanocarboxylic acid OR KABI in INTERVENTION

38 records for 37 trials found.

ClinicalTrials.gov

Menstru* OR menorrhagia (in condition)

AND

Antifibrinolytic* OR Anti-fibrinolytic* OR tranexamic OR TXA OR trans-4-aminomethylcyclohexanocarboxylic acid OR KABI (in intervention)

B List of systematic reviews searched

BITZER, J., HEIKINHEIMO, O., NELSON, A. L., CALAF-ALSINA, J. & FRASER, I. S. 2015. Medical Management of Heavy Menstrual Bleeding: A Comprehensive Review of the Literature. *Obstetrical & Gynecological Survey,* 70**,** 115-130.

BRYANT-SMITH, A. C., LETHABY, A., FARQUHAR, C. & HICKEY, M. 2018. Antifibrinolytics for heavy menstrual bleeding. *Cochrane Database of Systematic Reviews***,** 95.

EDER, S., BAKER, J., GERSTEN, J., MABEY, R. G. & ADOMAKO, T. L. 2013. Efficacy and safety of oral tranexamic acid in women with heavy menstrual bleeding and fibroids. *Women's health,* 9**,** 397-403.

LEMINEN, H. & HURSKAINEN, R. 2012. Tranexamic acid for the treatment of heavy menstrual bleeding: Efficacy and safety. *International Journal of Women's Health,* 4**,** 413-421.

MATTESON, K. A., RAHN, D. D., WHEELER, T. L., 2ND, CASIANO, E., SIDDIQUI, N. Y., HARVIE, H. S., MAMIK, M. M., BALK, E. M., SUNG, V. W. & SOCIETY OF GYNECOLOGIC SURGEONS SYSTEMATIC REVIEW, G. 2013. Nonsurgical management of heavy menstrual bleeding: a systematic review. *Obstetrics & Gynecology,* 121**,** 632-43.

NAOULOU, B. & TSAI, M. C. 2012. Efficacy of tranexamic acid in the treatment of idiopathic and non-functional heavy menstrual bleeding: A systematic review. *Acta Obstetricia Et Gynecologica Scandinavica,* 91**,** 529-537.

RAY, S. & RAY, A. 2016. Non-surgical interventions for treating heavy menstrual bleeding (menorrhagia) in women with bleeding disorders. *Cochrane Database of Systematic Reviews***,** 36.
