# Supplementary material for: Tranexamic acid for anemia in women of reproductive age: A systematic review of randomized trials
Source: PLoS One. 2026 Nov 6;21(11):e0362488. doi: 10.1371/journal.pone.0362488 (PMC13743017; doi:10.1371/journal.pone.0362488)
Supplement: Supplementary file 3 [file pone.0362488.s003.pdf]

Supplementary File 3 – List of excluded articles with reasons for exclusion (n=60)

Berendes et al. (2026), PLOS ONE. DOI: 10.1371/journal.pone.0362488

Summary of reasons for exclusion of full-text articles (N=60)

n=2	Excluded on publication type (no original research)
n=10	Exclude on design (no RCT)
n=10	Excluded on intervention (e.g. treatment arm could include either TXA or any other type of standard medical treatment)
n=37	Excluded on comparator (active comparator, rather than placebo or no treatment)
n=1	Other (as specified below)

Reference list of excluded studies with reason for exclusion

Note: Some articles might have been excluded for more than one reason, but only one reason is indicated below (generally, the one listed highest on the screening form)

Alanwar A, Abbas AM, Hussain SH, Elhawwary G, Mansour DY, Faisal MM, et al. Oral micronised flavonoids versus tranexamic acid for treatment of heavy menstrual bleeding secondary to copper IUD use: a randomised double-blind clinical trial. European Journal of Contraception & Reproductive Health Care. 2018;23(5):365-70.	Exclude on comparator
Andersch B, Milsom I, Rybo G. An objective evaluation of flurbiprofen and tranexamic acid in the treatment of idiopathic menorrhagia. Acta Obstetricia et Gynecologica Scandinavica. 1988;67(7):645-8.	Exclude on comparator
Anonymous (2005) Multicenter Study to Evaluate the Safety of XP12B in Women With Heavy Menstrual Bleeding Associated With Menorrhagia.	Exclude on design (no RCT)
Anonymous (2005) Treatment and Management of Women With Bleeding Disorders.	Exclude on comparator
Anonymous (2009). "The Assessment of the Minimal Effective and Tolerated Dose of Tranexamic Acid in Women With Menorrhagia Who Have Bleeding Disorders." clinicaltrials.gov. 15.	Exclude on design (no RCT)
Anonymous (2011). "Prospective Crossover Trial of Oral Tranexamic Acid and Combined Oral Contraceptive in Adolescents With Menorrhagia - A Pilot Study." clinicaltrials.gov. 01.	Exclude on comparator
Anomymous (2011). A Multi-center, Open Label Extension Study to Evaluate the Safety of an Oral Dose of Tranexamic Acid (XP12B) Administered Three Times Daily During Menstruation for the Treatment of Menorrhagia. In: Xanodyne P, ed.	Exclude on design (no RCT)
Anonymous (2013) Tranexamic Acid in Adolescents With Heavy Menstrual Bleeding.	Exclude on design (no RCT)
Anonymous (2015). "Oral Tranexamic Acid Versus Diosmin for Treatment of Menorrhagia in Women Using Copper IUD: Randomized Controlled Trial." clinicaltrials.gov. 22	Exclude on comparator

Anonymous (2017). "Tranexamic Acid Versus Calcium Dobesilate for the Treatment of Copper Intra Uterine Contraceptive Device Associated Heavy Menstrual Blood Loss : A Randomized , Open-labelled , Clinical Trial." clinicaltrials.gov. 30	Exclude on comparator
Anonymous (2021). "Tranexamic Acid Versus Depot-Medroxyprogesterone Acetate in the Treatment of Perimenopausal Irregular Uterine Bleeding: Randomized Clinical Trial." clinicaltrials.gov. 12.	Exclude on comparator
Anonymous (2023). "Comparative Study of Tranexamic Acid, Estrogen for Treatment Abnormal Uterine Bleeding in Depot-medroxyprogesterone Acetate Users. A Randomized Controlled Trial." clinicaltrials.gov. 13	Exclude on comparator
Anonymous (2024). "Randomized Trial of Mefenamic Acid Versus Tranexamic Acid in Management of Abnormal Uterine Bleeding." clinicaltrials.gov. 09	Exclude on comparator
Arora D, Chawla J, Kochar SPS, Sharma JC. A randomized control trial to assess efficacy of Mifepristone in medical management of uterine fibroid. Medical Journal Armed Forces India. 2017;73(3):267-73.	Exclude on comparator
Bonnar J, Sheppard BL. Treatment of menorrhagia during menstruation: Randomised controlled trial of ethamsylate, mefenamic acid, and tranexamic acid. British Medical Journal. 1996;313(7057):579-82.	Exclude on comparator
Berntorp E, Follrud C, Lethagen S. No increased risk of venous thrombosis in women taking tranexamic acid. Thrombosis and Haemostasis 2001;86(2):714-5.	Exclude on design (no RCT)
Byams, V. R., N. F. Dowling, J. A. Heit, P. A. Kouides, C. S. Philipp, S. F. Stein, A. S. Luke, C. H. Miller and R. Kulkami (2007). "Quality of life (QOL) evaluation in women with menorrhagia and abnormal hemostasis testing participating in a cross-over study of intranasal desmopressin and oral tranexamic acid." http://www isth2007 com/.	Exclude on comparator
Cooper KG, Parkin DE, Garratt AM, Grant AM. A randomised comparison of medical and hysteroscopic management in women consulting a gynaecologist for treatment of heavy menstrual loss. British Journal of Obstetrics & Gynaecology. 1997;104(12):1360-6.	Exclude on intervention
Ctri (2025). "Comparing Herbal Remedies and Tranexamic Acid for Managing Heavy Menstrual Bleeding: a Clinical Study." https://trialsearch.who.int/Trial2.aspx?TrialID=CTRI/2025/03/082736.	Exclude on comparator
Eder S, Baker J, Gersten J, Mabey RG, Adomako TL. Efficacy and safety of oral tranexamic acid in women with heavy menstrual bleeding and fibroids. Women's health. 2013;9(4):397-403.	Exclude on publication type (no original research)
Edlund M, Blomback M, Fried G. Desmopressin in the treatment of menorrhagia in women with no common coagulation factor deficiency but with prolonged bleeding time. Blood Coagulation & Fibrinolysis. 2002;13(3):225-31.	Exclude on intervention
Euctr GB. Randomised controlled trial of LNG-IUS (coil) against standard treatment (medical management) for patients suffering with Heavy Menstrual Bleeding. http://wwwwhoint/trialsearch/Trial2aspx?TrialID=EUCTR2004-001552-37-GB. 2005.	Exclude on intervention
Fathima A, Sultana A. Clinical efficacy of a Unani formulation 'Safoof Habis' in menorrhagia: A randomized controlled trial. European Journal of Integrative Medicine. 2012;4(3):E315-E22.	Exclude on publication type (no original research)

Fazmiya, M., A. Sultana, M. Heyat, S. Parveen, K. Rahman, F. Akhtar, A. Khan, A. Alanazi, Z. Ahmed, I. Díez, J. Ballester and T. Saripalli (2024). "Efficacy of a vaginal suppository formulation prepared with Acacia arabica (Lam.) Willd. gum and Cinnamomum camphora (L.) J. Presl. in heavy menstrual bleeding analyzed using a machine learning technique." Frontiers in Pharmacology 15: 23.	Exclude on comparator
Gennard L. Rates & predictors of progression to surgery for HMB: Results from the eclipse trial. Gynecological Surgery. 2012;9(1):S57-S8.	Exclude on intervention
Goshtasebi A, Mazari Z, Behboudi Gandevani S, Naseri M. Anti-hemorrhagic activity of Punica granatum L. flower (Persian Golnar) against heavy menstrual bleeding of endometrial origin: a double-blind, radomized controlled trial. Medical journal of the islamic republic of iran. 2015;29:199.	Exclude on comparator
Goshtasebi A, Moukhah S, Gandevani SB. Treatment of heavy menstrual bleeding of endometrial origin: randomized controlled trial of medroxyprogesterone acetate and tranexamic acid. Archives of Gynecology and Obstetrics. 2013;288(5):1055-60.	Exclude on comparator
Gupta J, Kai J, Middleton L, Pattison H, Gray R, Daniels J, et al. Levonorgestrel Intrauterine System versus Medical Therapy for Menorrhagia. New England Journal of Medicine. 2013;368(2):128-37.	Exclude on intervention
Gupta JK, Daniels JP, Middleton LJ, Pattison HM, Prileszky G, Roberts TE, et al. A randomised controlled trial of the clinical effectiveness and cost-effectiveness of the levonorgestrel-releasing intrauterine system in primary care against standard treatment for menorrhagia: the ECLIPSE trial. Health Technology Assessment. 2015;19(88):1-+.	Exclude on intervention
Gupta JK, Kai J, Middleton LJ, Pattison HM, Gray R, Gennard L, et al. Randomised trial of levonorgestrel intrauterine system compared to usual medical treatment for heavy menstrual bleeding: The ECLIPSE Trial. BJOG: An International Journal of Obstetrics and Gynaecology. 2013;120:379.	Exclude on intervention
Kai J, Middleton L, Daniels J, Pattison H, Tryposkiadis K, Gupta J, et al. Usual medical treatments or levonorgestrel-IUS for women with heavy menstrual bleeding: long-term randomised pragmatic trial in primary care. British Journal of General Practice. 2016;66(653):E861-E70.	Exclude on intervention
Kasonde JM, Bonnar J. Effect of ethamsylate and aminocaproic acid on menstrual blood loss in women using intrauterine devices. British Medical Journal 1975;4(5987):21-2.	Exclude on comparator
Kiseli M, Kayikcioglu F, Evliyaoglu O, Haberal A. Comparison of Therapeutic Efficacies of Norethisterone, Tranexamic Acid and Levonorgestrel-Releasing Intrauterine System for the Treatment of Heavy Menstrual Bleeding: A Randomized Controlled Study. Gynecologic and Obstetric Investigation. 2016;81(5):447-53.	Exclude on comparator
Kouides PA, Byams VR, Philipp CS, Stein SF, Heit JA, Lukes AS, et al. Multisite management study of menorrhagia with abnormal laboratory haemostasis: a prospective crossover study of intranasal desmopressin and oral tranexamic acid. British Journal of Haematology. 2009;145(2):212-20.	Exclude on comparator

Kouides PA, Heit JA, Philipp CS, Stein SF, Lukes AS, Skerrette NI. The effect of intranasal desmopressin vs. oral tranexamic acid on menstrual blood loss among women with menorrhagia and abnormal laboratory hemostasis: a multicenter, randomized cross-over study. Http://www isth2007 com/. 2007.	Exclude on comparator
Kouides, P. A., J. A. Heit, C. S. Philipp, S. F. Stein, A. S. Lukes, V. R. Byams, N. F. Dowling, B. L. Evatt, C. R. Miller, S. Owens and et al. (2007). "A Multi-Site, Prospective Cross-Over Study of Intranasal Desmopressin and Oral Tranexamic Acid in Women with Menorrhagia and Abnormal Laboratory Hemostasis." Blood 110(11).	Exclude on comparator
Kriplani A, Kulshrestha V, Agarwal N, Diwakar S. Role of tranexamic acid in management of dysfunctional uterine bleeding in comparison with medroxyprogesterone acetate. Journal of Obstetrics & Gynaecology. 2006;26(7):673-8.	Exclude on comparator
Lee, J.Y., Hahn, P.M., Van Dijk, J.P., and Reid, R.L. Treatment of Menorrhagia with Tranexamic Acid. Journal SOGC, Volume 22, Issue 10, 794 - 798	Exclude on design (no RCT)
Lee, J. Y., Reid, R. L. and Van Dijk, J. P. (1997). "Treatment of menorrhagia with tranexamic acid." Fertility and sterility: S96.	Exclude – other (conference abstract that mentions two trials: no comparison group for 1st trial and insufficient information on ongoing 2nd trial.)
Milsom, I. (1991). "A Comparison of Flurbiprofen, Tranexamic Acid and a Levonorgestrel-releasing Intrauterine Contraceptive Device in the Treatment of Idiopathic Menorrhagia." American journal of obstetrics and gynecology 164(3): 879-883.	Exclude on comparator
Muse K, Gersten J, Waldbaum A, Mabey RG, Lukes A, Moore K. Long-term quality of life and safety of a new tranexamic acid menorrhagia treatment. Fertility and Sterility. 2010;94(4):S13.	Exclude on design (no RCT)
Muse K, Gersten J, Waldbaum A, Mabey RG, Lukes A, Shangold G. Novel, oral tranexamic acid improves the general quality of life (SF-36v2TM health survey measures) in women with menorrhagia. Fertility and Sterility. 2010;94(4):S208-S9.	Exclude on design (no RCT)
Najam R, Agarwal D, Tyagi R, Singh S. Comparison of traneximic acid with a combination of traneximic acid and mefenamic acid in reducing menstrual blood loss in ovulatory dysfunctional uterine bleeding (DUB). Journal of Clinical and Diagnostic Research. 2010;4(5):3020-5.	Exclude on comparator
Nct (2024). "Management of Abnormal Uterine Bleeding." https://clinicaltrials.gov/ct2/show/NCT06369012.	Exclude on comparator
NCT01190150. Lysteda Pediatric Research Equity Act (PREA) pharmacokinetic study in adolescent females with heavy menstrual bleeding. clinicaltrials.gov/ct2/show/NCT01190150 (first received 27 August 2010).	Exclude on design (no RCT)
Nilsson L, Rybo G. Treatment of menorrhagia with epsilon aminocaproic acid. A double blind investigation. Acta Obstetricia et Gynecologica Scandinavica 1965;44(3):467-73.	Exclude on intervention
Preston JT, Cameron IT, Adams EJ, Smith SK. Comparative study of tranexamic acid and norethisterone in the treatment of ovulatory menorrhagia. British Journal of Obstetrics & Gynaecology. 1995;102(5):401-6.	Exclude on comparator

Preston, J., I. Cameron, E. Adams and S. Smith (1993). "Comparison of Tranexamic Acid and Norethisterone in the Treatment of Ovulatory Menorrhagia." 2nd international scientific meeting of the royal college of obstetricians: 85.	Exclude on comparator
Ragab MI, Thomas MN. The use of tranexamic acid (AMCA) in IUDs as an antibleeding agent. INTJGYNAECOBSTET. 1976;14(2):137-41.	Exclude on design (no RCT)
Ragni MV et al. Von willebrand disease minimize menorrhagia (VWDMin) trial. Blood. 2019;134.	Exclude on comparator
Ragni MV, Seaman CD, Gilligan D, Philipp CS, Neff AT, Sidonio RF, et al. Von willebrand disease minimize menorrhagia (VWDMin) trial. Blood Conference: 61st Annual Meeting of the American Society of Hematology, ASH. 2019;134(Supplement 1).	Exclude on comparator
Saharkhiz N, Ehdaeevand F, Tavana A, Majdfar Z, Fallahian M. A Comparative Trial of the Efficacy of Mefenamic Acid and Tranexamic Acid for Treatment of Menorrhagia Induced by Copper T-380A IUD. International Journal of Womens Health and Reproduction Sciences. 2017;5(3):175-80.	Exclude on comparator
Sanghera S, Roberts TE, Barton P, Frew E, Daniels J, Middleton L, et al. Levonorgestrel-releasing intrauterine system vs. usual medical treatment for menorrhagia: An economic evaluation alongside a randomised controlled trial. PLoS ONE. 2014;9(3).	Exclude on intervention
Shafiee M, Heidari A, Amouzegar H, Khani S, Nojavan F. Evaluation of the effect of roasted lentil flour (lentil savigh) as a functional food in menstrual bleeding reduction. Complementary Therapies in Medicine. 2019;44:27-31.	Exclude on comparator
Slctr. Tranexamic acid with norethisterone versus tranexamic acid alone in treatment of vaginal bleeding during dengue infection. http://wwwwhoint/trialsearch/Trial2aspx?TrialID=SLCTR/2017/037. 2017.	Exclude on comparator
Srivaths LV, Dietrich JE, Yee DL, Sangi-Haghpeykar H, Mahoney D. Oral Tranexamic Acid versus Combined Oral Contraceptives for Adolescent Heavy Menstrual Bleeding: A Pilot Study. Journal of Pediatric and Adolescent Gynecology. 2015;28(4):254-7.	Exclude on comparator
Tabatabaei A. A clinical randomized single blind trial of medical therapies for menorrhagia using ibuprofen and tranexamic acid. International Journal of Fertility and Sterility. 2013;7:120.	Exclude on comparator
J. Y. Lee, P. M. Hahn, J. P. Van Dijk and R. L. Reid. Treatment of Menorrhagia with Tranexamic Acid Journal SOGC, Volume 22, Issue 10, 794 - 798	Exclude on design (no RCT)
Umarami RBR, Roqaiya M, Quadri MA. Efficacy of Habbul aas (fruits of Myrtus communis) in menorrhagia: A single blinded randomized standard control study. Journal of Complementary and Integrative Medicine. 2020.	Exclude on comparator
Zhang YW, He FF, Sun ZY, Li SW, Bi SL, Huang XL, et al. [A multicenter prospective randomized open comparative study on the treatment of ovulatory menorrhagia with tranexamic acid and norethisterone in China]. Chung-Hua Fu Chan Ko Tsa Chih [Chinese Journal of Obstetrics & Gynecology]. 2008;43(4):247-50.	Exclude on comparator
Zhang, Y., F. He, S. Li, Z. Cao, S. Lv and J. Lu (2007). "A multicenter, prospective, randomized, open comparator study on the treatment of ovulatory menorrhagia with tranexamic acid and norethisterrone in mainland China." Fertility and sterility 88 Suppl 1: 17-18, Abstract no: 47.	Exclude on comparator
