# Supplementary material for: Tranexamic acid for anemia in women of reproductive age: A systematic review of randomized trials
Source: PLoS One. 2026 Nov 6;21(11):e0362488. doi: 10.1371/journal.pone.0362488 (PMC13743017; doi:10.1371/journal.pone.0362488)
Supplement: Supplementary file 4 [file pone.0362488.s004.pdf]

Supplementary File 4 – List of ongoing trials with main characteristics

(Berendes et al. (2026), PLOS ONE, DOI: 10.1371/journal.pone.0362488)

Table S4 – Characteristics of included ongoing trials

Study (country)	Registration no. (Name)	Design	Aim/objective	Participants, Planned (or actual) total number, N	Treatment dose, timing, duration & control	Outcomes relevant to review
Anonymous 2023[1] (USA)	NCT06000424	RCT, 1:1, parallel	To determine whether TXA is an efficacious treatment for contraceptive induced menstrual changes, incl. irregular, bothersome bleeding caused by contraceptive implants	Female participants aged 15-45 yrs with irregular menstrual bleeding caused by the etonogestrel subdermal contraceptive implant, N=65	1.3 g TXA orally 3x/day for 5 days treatment versus placebo started after three consecutive days of contraceptive induced bleeding	*Primary:* Difference in mean days without bleeding across 30-day reference period
Anonymous 2024[2] (Canada)	NCT06452342 (T-REX HMB trial)	Pilot RCT, 1:1, parallel	To assess the feasibility of a full trial comparing TXA to placebo in decreasing HMB in premenopausal individuals anticoagulated for VTE.	Adult premenopausal individuals anticoagulated for VTE with HMB, N=20	1.5 g TXA orally 3x/day during menstruation versus no TXA,<br>3 months	*Primary:* Participant recruitment<br>*Secondary:* Participant adherence/ compliance and retention;<br>MBL, subj.; major and/or clinically relevant bleeding events; Ferritin; Hb; Recurrent VTE; symptoms of post-thrombotic syndrome;<br>QoL after pulmonary embolism;<br>General health-related QoL
ChiCtr 2022[3]	CN-02536522		To verify the efficacy and safety of Yuzhi Zhixue Granules for AUB. 2. To explore the comparison of hemostatic effect and safety of Yuzhi Zhixue Granules and TXA Tablets.	Participants with abnormal uterine bleeding, (N not reported)	TXA plus Yuzhi Zhixue Granules vs<br>TXA mimic (placebo) plus Yuzhi Zhixue Granule	*Primary:* MBL, subj.; bleeding duration; Hb; prothrombin time; changes in endometrial thickness;
NCT 2024[4]	NCT06519422<br>CN-02738290<br>(WOMAN-3/ TXA for anaemia trial)	RCT, 1:1, parallel	To determine the effect of giving TXA during menstruation for anaemia treatment.	Adult women with anaemia, regular cycles; N=4000	1 g TXA (2 tbl) orally 3x/day versus matched placebo from first to last day of menstruation for up to 5 days	*Primary:* Anaemia (Hb<120g/L)<br>*Secondary:* Hb; anaemia severity; Ferritin; Iron deficiency; C-reactive protein; Iron deficiency anaemia; MBL, subj.; Perceived change in MBL; MBL-related QoL; flooding

AUB, abnormal uterine bleedingg, gram; Hb, haemoglobin; HMB, heavy menstrual bleeding; MBL, menstrual blood loss; no., number; parallel, parallel-group trial; QoL, quality of life; RCT, randomized controlled trial; subj., subjective assessment method; tbl., tablet; TXA, tranexamic acid; VTE, venous thromboembolism.

References

1. Anonymous. Tranexamic Acid to Reduce Contraceptive-related Bleeding Side Effects. *clinicaltrialsgov* 2023;14
2. Anonymous. TRanEXamic Acid to Decrease Heavy Menstrual Bleeding in Individuals Anticoagulated for Venous Thromboembolism Pilot Study. *clinicaltrialsgov* 2024;05
3. ChiCtr. A multi-center, randomized, double-blind controlled clinical study of Yuzhi Zhixue Granules and tranexamic acid in the treatment of abnormal uterine bleeding. https://trialsearchwhoint/Trial2aspx?TrialID=ChiCTR2200059418 2022
4. Nct. Tranexamic Acid for Anaemia Trial. https://clinicaltrials.gov/study/NCT06519422 2024
