# Supplementary material for: Tranexamic acid for anemia in women of reproductive age: A systematic review of randomized trials
Source: PLoS One. 2026 Nov 6;21(11):e0362488. doi: 10.1371/journal.pone.0362488 (PMC13743017; doi:10.1371/journal.pone.0362488)
Supplement: Supplementary file 5 [file pone.0362488.s005.pdf]

Supplementary file 5 – Detailed characteristics of included trials (n=17)

Berendes et al. (2026), PLOS ONE. DOI: 10.1371/journal.pone.0362488

Adawiyah (2020)[1]

Methods	*Design (trial name):*	RCT, 1:1 parallel
	Trial dates/ duration:	Oct 2016 to Aug 2017 (28 treatment days per participant)
	Trial registration:	Not reported
Setting	*Country:*	Indonesia
	Recruitment setting/ location:	Kariadi General Hospital in Semarang
Participants	*Participants:*	Women using contraceptive DMPA injection with AUB, defined as bleeding or spotting for ≥ 8 days/month;<br>Inclusion criteria were being a new DMPA users, age 20-45 years old, and a normal pelvic examination.
	Total number:*	N=44
	Age:	Mean age - TXA group: 31 yrs, Placebo group: 26 yrs
Intervention	*Treatment dose, timing, duration:*	250 mg TXA orally 4x/day for 5 days (single treatment course) plus<br>OCP (30 mcg ethinylestradiol & 150mg levonorgestrel) 1x/day for 28 days
	Comparator:	Placebo (same dose, timing, duration as TXA) plus OCP (same as TXA group)
	Notes:	n/a
Outcomes^	*Objective:*	n/a
	Subjective/ self-reported:	Mean bleeding length at endpoint (28 days post-randomization),<br>Side effects (reported throughout study period)
	Note:	
Other	*Funding:*	Not reported
	Ethics approval:	Approved by the Ethics Committee of Faculty of Medicine Diponegoro University and dr. Kariadi Hospital number 936/EC/FK-RSDK/IX/2016
	Conflict of interest:	Not reported
	Notes:	

Callender (1970)[2]

Methods	*Design (trial name):*	Crossover trial
	Trial dates/ duration:	Not reported (total of 8 menstrual cycles per participant)
	Trial registration:	Not reported
Setting	*Country:*	United Kingdom
	Recruitment setting/ location:	Not specified
Participants	*Participants:*	Patients with iron-deficiency anaemia presumed to be due to MBL or referred to gynaecologist with HMB complaint;<br>w/o clinical or histological gynaecological abnormality
	Total number:*	N=20 (N=16 were analysed)
	Age:	33-49 yrs
Intervention	*Treatment dose, timing, duration:*	Initial three control cycles: no treatment<br>Three treatment cycles (random assignment to 1st or 2nd phase):<br>TXA (Cyclokapron) 1g (2 tbl.) 4x/day for first 4 days of menstruation

	Comparator:	Placebo of similar size and appearance as TXA (same dose, timing and duration)
	Notes:	Some participants were treated with iron before and/or during the trial
Outcomes^	*Objective:*	MBL (mean loss/period, ml) assessed via Oxford total body counter method: 2-4 uCi ^{59}Fe given i.v. at baseline; blood loss estimated from loss of radioactivity x total blood volume after treatment period, Warner and Oliver, 1966[3]; 2-weekly measurements)
	Subjective/ self-reported:	Number of pads used per cycle; data from 14 participants analysed who used same type of menstrual products); Duration of menstrual loss (days); Side effects reported throughout study period (3 TXA and 3 Placebo cycles)
	Note:	
Other	*Funding:*	Kabi Pharmaceuticals Limited provided supplies of Cyclokapron and contributed towards the expenses of the investigation.
	Ethics approval:	Not reported
	Conflict of interest:	Not reported
	Notes:	Some participants also received iron supplementation during trial

Drosdal (1983)[4] [Norwegian]

Methods	*Design (trial name):*	Crossover trial
	Trial dates/ duration:	Not reported
	Trial registration:	Not reported
Setting	*Country:*	Norway
	Recruitment setting/ location:	Nine general practitioners in one municipality
Participants	*Participants:*	Women (37±7 yrs) with HMB and/or HMB related iron deficiency anaemia (Inclusion criteria: Women who believed that the amount of menstrual bleeding was an unacceptable, limiting factor for carrying out activities of daily living and profession, and who had a daily consumption of >6 pads/tampons; Iron deficiency anaemia related to HMB; A combination of the first two criteria where a total assessment of the patient made that fibrinolysis inhibition was the first treatment alternative. Women who had previously been treated with TXA should have at least one treatment-free cycle before inclusion.)
	Total number:*	N=44 or 37 (unclear which of these used in analysis)
	Age:	37±7 yrs
Intervention	*Treatment dose, timing, duration:*	TXA (Cyklokapron) 1g (2 tbl.) 3x/day from **2nd** to 5th day of menstruation during 3 cycles
	Comparator:	Placebo (same dose, timing, duration as TXA)
	Notes:	n/a
Outcomes^	*Objective:*	Hb (but not pooled, as presented data insufficient)
	Subjective/ self-reported:	MBL assessed via visual analogue scale (VAS, but not pooled, as presented data insufficient); Number of tampons/pads used (but not pooled, as presented data insufficient); Side effects
	Note:	Quality of Life outcomes mentioned in methods, but results not reported
Other	*Funding:*	Not reported
	Ethics approval:	Not reported
	Conflict of interest:	Not reported

Edlund (1995)[5]

Methods	*Design (trial name):*	RCT, 2:2:1, parallel
	Trial dates/ duration:	Not reported
	Trial registration:	Not reported
Setting	*Country:*	Sweden
	Recruitment setting/ location:	Multi-centre trial at three medical centres (department of obstetrics & gynaecology at three university hospitals)
Participants	*Participants:*	Women with HMB (>80ml MBL/cycle) & regular cycles
	Total number:*	N=91 (N=68 'fulfilled' the study)
	Age:	>18 yrs
Intervention	*Treatment dose, timing, duration:*	Prodrug of TXA (Kabi 2161)<br>Group A: 1.2g/day (2 tbl.) 2x/day & Group B: 0.6g/day (1 tbl.) 4x/d; treatment taken for first 5 days of menstruation for 3 cycles
	Comparator:	Placebo (group C), 2 tbl. 4x/day for first 5 days of menstruation for 3 cycles
	Notes:	n/a
Outcomes^	*Objective:*	MBL assessed using alkaline haematin method as described by Hallberg & Nilsson (1964)[6] and modified by Newton et al. (1977)[7]
	Subjective/ self-reported:	MBL (patient estimate, perceived change during treatment)<br>No. of tampons/pads used during 3 cycles (average per cycle)<br>Duration of bleeding (days)
	Note:	Various laboratory tests completed, incl. for Hb, but results not reported; Side effects reported, but not presented by treatment group
Other	*Funding:*	Not reported
	Ethics approval:	Study design was approved by the local ethics committee of each centre.
	Conflict of interest:	Not reported
	Notes:	

Freeman (2011)[8]

Methods	*Design (trial name):*	RCT, approx. 2:2:1, parallel
	Trial dates/ duration:	4th December 2006 to 29th May 2008
	Trial registration:	NCT00401193
Setting	*Country:*	USA
	Recruitment setting/ location:	Outpatient, multicentre, 63 sites
Participants	*Participants:*	Women with a history of cyclic HMB and objectively confirmed MBL ≥ 80ml per cycle during 2 months screening phase<br>*Inclusion criteria:* normal findings on pelvic examination, at cervical cytology screening, and on transvaginal ultrasonograms.<br>*Exclusion criteria:* history or presence of clinically significant disease, anovulatory dysfunctional uterine bleeding, metrorrhagia, menometro-rrhagia, polymenorrhea, endometrial polyps, endometrial hyperplasia, endometrial carcinoma, cervical carcinoma, myocardial infarction, ischemic disease, cerebrovascular accident, stroke, transient ischemic attack, thrombosis thromboembolic disease, or coagulopathy and other relevant clinically significant abnormalities; severe anaemia (Hb <8 g/dL).
	Total number:*	N=304 people randomised, N=297 included in safety analysis and N=294 in modified ITT efficacy analysis
	Age:	18-49 years
Intervention	*Treatment dose, timing, duration:*	0.65g TXA (1st group) or 1.3g TXA (2nd group) 3x/d vs placebo (3rd group) for up to 5 consecutive days during menstruation, 3 cycles

	Comparator:	Placebo (same dose, timing, duration as TXA)
	Notes:	Prohibition of certain medications during entire study period (e.g., hormonal contraceptives) or during menstruation (e.g. nonsteroidal anti-inflammatory drugs)
Outcomes^	*Objective:*	MBL (AH method; final blood loss means at 3 months post-randomisation)
	Subjective/ self-reported:	HR-QoL, subj. (MIQ, q.3&4 on physical & social or leisure activities)<br>Adverse events (reported throughout 6 months study period)
	Note:	
Other	*Funding:*	Ferring Pharmaceuticals Inc.
	Ethics approval:	Approved by the institutional review board at each study sites.
	Conflict of interest:	All but one of the co-authors reported that they either work for or have received funding from one or more pharmaceutical companies.
	Notes:	

Hamulyák (2024)[9 10]

Methods	*Design (trial name):*	Pragmatic open-label RCT, approximate 1:1:1 parallel (MEDEA trial)
	Trial dates/ duration:	February 2020 to November 2022 (recruitment period)
	Trial registration:	Netherlands Trial Register: NL7760
Setting	*Country:*	Netherlands
	Recruitment setting/ location:	The trial was embedded in routine clinical care in 3 academic and 12 non-academic hospitals.
Participants	*Participants:*	Adult premenopausal women treated with a factor Xa inhibitor (either apixaban, edoxaban, or rivaroxaban) in any dose for any indication in whom HMB has occurred and who have one prospective PBAC score above 150 (semi-quantitative method to assess HMB)<br>Participants needed to have an indication for anticoagulant treatment for >3 months after inclusion; Exclusion criteria included concomitant use of hormonal therapy as a new intervention for HMB, active malignancy, current treatment with chemotherapy, immunotherapy, and/or radiotherapy, and cervical preneoplastic lesions.
	Total number:*	N=16 (n=5 in FXa inhibitor plus TXA group, n=7 in FXa inhibitor only group)
	Age:	≥ 18 years; Mean age (SD):<br>- Continued FXa inhibitor plus TXA group: 42.0 (6.4) years<br>- Continued FXa inhibitor without TXA group: 40.9 (8.4) years<br>[- Dabigatran group: 38.3 (11.2) years]
Intervention	*Treatment dose, timing, duration:*	Continued FXa inhibitor with addition of 1000 mg TXA 3x a day on the first day of menstrual bleeding for 4 days
	Comparator:	Continued FXa inhibitor without TXA
	Notes:	Third treatment group was switch to Dabigatran; Treatment of any other potential underlying disorder or concomitant disease, for instance, iron deficiency anaemia, was at the discretion of the treating physician and not part of study procedures.
Outcomes^	*Objective:*	(Hb assessed, but not reported)
	Subjective/ self-reported:	Primary outcome: MBL (PBAC score),<br>[Secondary outcomes included quality of life, duration of menstrual bleeding, intermenstrual bleeding, but were not reported]<br>Adverse events
	Note:	
Other	*Funding:*	An unrestricted investigator-initiated research grant by Boehringer Ingelheim (BI); BI had no role in the design, analysis, or interpretation of the results in this study; BI was given the opportunity to review the

		manuscript for medical and scientific accuracy as it relates to BI substances, as well as intellectual property considerations.
	Ethics approval:	Approval from the Medical Ethical Committee of the Amsterdam University Medical Centers, Amsterdam, The Netherlands.
	Conflict of interest:	Authors indicated that there were no competing interests to disclose.
	Notes:	The study was stopped prematurely due to the low inclusion rate. (Original estimated sample size was N=120)

Lin (2007)[11]

Methods	*Design (trial name):*	RCT, 1:1:1, parallel (with additional randomisation for obj. outcomes)
	Trial dates/ duration:	Not reported
	Trial registration:	Not reported
Setting	*Country:*	China
	Recruitment setting/ location:	Four Maternal and Child Health Centres in Shanghai
Participants	*Participants:*	Women aged 20-40 years with regular menstrual cycles attending for a VCu-intrauterine device (IUD);<br>*Exclusion criteria:* medical histories of organic diseases, concomitant disease influencing menstruation
	Total number:*	N=175 for subjective MBL outcome (N=195 originally randomised),<br>N=64 for objective MBL outcome
	Age:	20-40 years
Intervention	*Treatment dose, timing, duration:*	Group 1: 1g TXA orally twice daily; Group 2: 500 mg TXA orally twice daily during first 5 days of menstruation for 3 cycles after IDU insertion (after treatment-free run-in phase)
	Comparator:	Placebo (same dose, timing, duration as TXA)
	Notes:	Prohibition of contraceptive pills
Outcomes^	*Objective:*	MBL (AH method); occurrence of HMB (>80 ml MBL)
	Subjective/ self-reported:	MBL (PBAC); occurrence of HMB (138 PBAC score)
	Note:	
Other	*Funding:*	Financial support: Daiichi Pharmaceutical Company Ltd., International Division (14-10, Nihonbashi 3-Chome Chuo-ku, Tokyo 103-8234 Japan)<br>Technical support: National Population and Family Planning Commission of China
	Ethics approval:	Not reported
	Conflict of interest:	Not reported
	Notes:	

Lukes (2010)[12]

Methods	*Design (trial name):*	RCT, 2:1 parallel
	Trial dates/ duration:	The first participant was screened on October 16, 2006, and the last study visit was May 8, 2008.
	Trial registration:	NCT00386308
Setting	*Country:*	USA
	Recruitment setting/ location:	Multicentre, 40 clinical sites
Participants	*Participants:*	Women with HMB (≥80 ml/cycle)<br>*Inclusion criteria:* history of ≥ 3 consecutive days of HMB over at least four of their last six menstrual periods; during a two-cycle pretreatment baseline phase, menstrual blood loss of ≥ 60 mL during the first menstrual period and average of ≥ 80 mL over both pretreatment cycles; normal findings on pelvic examination; regular menstrual periods (≤10 days in duration and 21-35 days cycle length); if in childbearing potential, required to use an acceptable nonhormonal method of birth control.<br>*Exclusion criteria:* clinically important cervical cytology abnormalities or uterine pathologic findings by transvaginal ultrasonography (if abnormal findings, then normal endometrial biopsy results required); history or presence of significant medical problems (e.g., thromboembolic disease, coagulopathy, or ocular disease); history or presence of endometrial abnormalities or cervical carcinoma; had anovulatory dysfunctional uterine bleeding, metrorrhagia, menometrorrhagia, or polymenorrhea; abnormal colour vision, glaucoma, ocular hypertension, macular degeneration, or retinopathies; severe anaemia (Hb< 8 g/dL)
	Total number:*	N=196 randomised (N=187 intent-to-treat population)
	Age:	18-49 yrs.
Intervention	*Treatment dose, timing, duration:*	TXA (Lysteda) 1.3g (2 tbl.) 3x/d at menstruation onset for up to 5d/cycle, 6 cycles [potentially plus oral iron therapy]
	Comparator:	Matched placebo (same dose, timing, duration as TXA) [potentially plus oral iron therapy]
	Notes:	Oral iron therapy was prescribed at the investigator's discretion for women with Hb levels between 11 g/dL and 12 g/dL at baseline. Oral iron therapy was required for women with baseline Hb < 11 g/dL and for those individuals whose Hb levels declined to < 11 g/dL during the study; Prohibition of certain medications during entire study period (e.g., hormonal contraceptives) or during menstruation (e.g. nonsteroidal anti-inflammatory drugs)
Outcomes^	*Objective:*	MBL (AH method; final blood loss means at 6 months post-randomisation); Hb (not pre-specified by investigators)
	Subjective/ self-reported:	MBL (number of large blood stains; perceived MBL score, MIQ q.1)<br>HR-QoL, subj. (MIQ, q.3&4 on physical & social or leisure activities)<br>Adverse events (reported throughout 6 months study period)
	Note:	Imbalances between groups at baseline, e.g. more women had anaemia in TXA arm (34%) than placebo arm (18%) and mean MBL was higher in TXA arm (172.3+/- 95.6) than placebo arm (153+/-66.6);
Other	*Funding:*	Xanodyne Pharmaceuticals, Inc., and Ferring Pharmaceuticals, Inc.
	Ethics approval:	Protocol received approval from each site's Institutional Review Board.
	Conflict of interest:	All but one of the co-authors reported that they either work for or have received funding from one or more pharmaceutical companies.
	Notes:	

Nilsson (1967)[13]

Methods	*Design (trial name):*	Crossover trial
	Trial dates/ duration:	Not reported
	Trial registration:	Not reported
Setting	*Country:*	Sweden
	Recruitment setting/ location:	Hospital Department of Obstetrics and Gynaecology
Participants	*Participants:*	Women aged 15-49 referred to the Department of Obstetrics and Gynaecology for suspected menorrhagia.
	Total number:*	N=36
	Age:	Mean age 38 years
Intervention	*Treatment dose, timing, duration:*	After 2 cycles without treatment, each participant had each of the following for 1 menstrual cycle in random order (original design):<br>0.25g AMCA or 0.5g AMCA 6x/d or placebo for first 4 days of menstruation, 1 cycle each; However, during the trial (after N=8 participants), the trial treatment dosage was increased to:<br>0.5 g (2x0.25g tbl.) AMCA or 1 g (2x 0.5g) AMCA 6x/d or placebo for first 4 days of menstruation, 1 cycle each
	Comparator:	Placebo (same dose, timing, duration as TXA)
	Notes:	n/a
Outcomes^	*Objective:*	MBL (AH method, assessed after 1 cycle of respective treatment)
	Subjective/ self-reported:	Side effects
	Note:	
Other	*Funding:*	Not reported
	Ethics approval:	Not reported
	Conflict of interest:	Not reported
	Notes:	

Petersen (1983)[14]

Methods	*Design (trial name):*	Crossover trial
	Trial dates/ duration:	November 1979 and August 1981 (recruitment period)
	Trial registration:	Not reported
Setting	*Country:*	Denmark
	Recruitment setting/ location:	Not specified
Participants	*Participants:*	Women with HMB (>80 ml), regular cycle and partially incapacitated during their menstrual periods
	Total number:*	N=17
	Age:	18-45 years
Intervention	*Treatment dose, timing, duration:*	Initial two control cycles: no treatment<br>Then two treatment cycles, i.e. randomised for alternating treatment with TXA or placebo for 1 cycle each.<br>TXA: 3 x 0.5g (3 tbl.) every 8 hours (=3x/day) from day 1-4 of menstruation
	Comparator:	Placebo
	Notes:	'No other medication was given during study period.'
Outcomes^	*Objective:*	MBL (AH method)

	Subjective/ self-reported:	Bleeding duration (days); absence from work; side effects; proportion of participants who wished to continue taking TXA after end of study
	Note:	
Other	*Funding:*	Not reported
	Ethics approval:	Not reported
	Conflict of interest:	Not reported
	Notes:	

Phupong (2006)[15]

Methods	*Design (trial name):*	RCT, 1:1 parallel
	Trial dates/ duration:	Not reported
	Trial registration:	Not reported
Setting	*Country:*	Thailand
	Recruitment setting/ location:	Family Planning Clinic, Department of Obstetrics and Gynecology, Faculty of Medicine, Chulalongkorn University, Bangkok
Participants	*Participants:*	Women with irregular uterine bleeding secondary to Norplant use: Norplant users (six-capsule levonorgestrel subdermal implants) who came to the clinic with current bleeding or spotting and a complaint of a bleeding disturbance. Bleeding disturbances were defined as bleeding or spotting for ≥8 continuous days or a current bleeding episode initiated after a bleeding-free interval of ≤14 days<br>*Inclusion criteria:* Norplant inserted 3–36 months before enrolment and body mass index (BMI) of 19 -30 kg/m2<br>*Exclusion criteria:* gynaecological or medical diseases, contraindication to tranexamic acid and previous treatment for Norplant-related bleeding within 3 months before the recruitment.
	Total number:*	N=68
	Age:	Mean age - TXA group: 27.0 +/- 5.7 years; Placebo group: 29.1 +/- 6.1 years
Intervention	*Treatment dose, timing, duration:*	TXA (Transamin[R], Ouiheng, Bangkok, Thailand), two capsules (250 mg/ capsule) 4 x/ day for 5 days (one-off treatment, follow-up at 1 week and 4 weeks post-treatment initiation)
	Comparator:	Placebo capsules which appeared to be similar to TXA (same dose, timing, duration as TXA)
	Notes:	n/a
Outcomes^	*Objective:*	n/a
	Subjective/ self-reported:	Primary outcomes: Cessation of bleeding during first week after the initiation of 5-day treatment; maintenance of a bleeding-free interval of >20 days within a 4-week period of follow-up;<br>adverse effects
	Note:	
Other	*Funding:*	Financial support from the Ratchadapiseksompotch Fund, Faculty of Medicine, Chulalongkorn University, Bangkok, Thailand
	Ethics approval:	Approved by the Ethics Committee of the Faculty of Medicine, Chulalongkorn University
	Conflict of interest:	Not reported
	Notes:	

Rivera (1984)[16]

Methods	*Design (trial name):*	RCT, 1:1 parallel
	Trial dates/ duration:	Not reported
	Trial registration:	Not reported
Setting	*Country:*	Mexico and/or USA (unclear)
	Recruitment setting/ location:	Not specified
Participants	*Participants:*	Women having Lippes Loop D (LLD) Intrauterine Device (IUD) inserted
	Total number:*	N=21 randomised (N=18 at 1 month FU, N=17 at 3 months FU – unclear if ITT analysis)
	Age:	Mean age, TXA group: 27.5 years, Control group: 28.2 years
Intervention	*Treatment dose, timing, duration:*	LLD-IUD modified to provide a sustained release of AMCA (TXA); each loop contained 92 mg of AMCA dispersed 40% by weight in a Dupont 3165 ethylene vinyl acetate matrix (*in vitro* data showed an AMCA release rate between 300 and 400 ug at 1 month, between 100 and 150 ug at 3 month and slightly below 100ug/d at 6-month post-insertion)
	Comparator:	LLD-IUD that had the same physical alterations as the AMCA-loaded devices except that the reservoirs were empty
	Notes:	n/a
Outcomes^	*Objective:*	Pre- vs post-insertion MBL increase (AH method) at 1, 2, 3 & 6 months post-IUD insertion
	Subjective/ self-reported:	Women's perception of changes in cycle length and flow duration at 1, 3 and 6 months post-insertion; dysmenorrhoea; intermenstrual bleeding
	Note:	At 6 months post-insertion, the TXA release had dropped from >400 ug/day to <100 ug/day according to *in vitro* data; at 6 months control group data available for only 4 of 10 women
Other	*Funding:*	Partial support provided by Family Health International with funds from the United States Agency for International Development
	Ethics approval:	Not reported
	Conflict of interest:	Not reported
	Notes:	

Senthong (2009)[17]

Methods	*Design (trial name):*	RCT, 1:1 parallel
	Trial dates/ duration:	Not reported
	Trial registration:	Not reported
Setting	*Country:*	Thailand
	Recruitment setting/ location:	Family Planning Clinic, Department of Obstetrics and Gynecology, Chulalongkorn Hospital, Bangkok
Participants	*Participants:*	Women with irregular uterine bleeding secondary to contraceptive injection (Depot-medroxyprogesterone acetate, DMPA, a long acting progestine)<br>*Inclusion criteria*: complaint of bleeding disturbances, defined as bleeding or spotting for ≥ 8 continuous days or a current bleeding episode initiated after a bleeding-free interval of ≤14 days – participants were eligible if indicating ≥ 8 days or more of a bleeding or spotting prior to obvious bleeding on the day of admission; being DMPA users for 1-18 months before enrolling in the present study; a body mass index between 18-30 kg/m2; age 20-45 years; having a normal pelvic examination.

		Exclusion criteria: any gynecological disease that could cause abnormal uterine bleeding; any medical disease that would cause bleeding tendency; contraindication to tranexamic acid; a history of hepatic impairment or renal insufficiency; previous treatment for DMPA-related bleeding for a period of 3 months prior to recruitment.
	Total number:*	N=100 (N=99 included in analysis)
	Age:	Mean age - TXA group: 29.64 +/- 7.10, placebo group: 28.69 +/- 7.67
Intervention	*Treatment dose, timing, duration:*	250 mg TXA 4x/day for 5 days (single treatment course at 1 timepoint)
	Comparator:	Placebo (same dose, timing, duration as TXA)
	Notes:	n/a
Outcomes^	*Objective:*	n/a
	Subjective/ self-reported:	Cessation of bleeding during first week after the initiation of 5-day treatment; maintenance of a bleeding-free interval of >20 days within a 4-week period of follow-up; adverse effects
	Note:	
Other	*Funding:*	Not reported
	Ethics approval:	Approved by the Ethics Committee of the Faculty of Medicine, Chulalongkorn University.
	Conflict of interest:	Not reported
	Notes:	

Sordal (2013)[18]

Methods	*Design (trial name):*	RCT, 1:1:1 parallel
	Trial dates/ duration:	March – December 2011
	Trial registration:	NCT01295294
Setting	*Countries:*	Denmark, Ireland, and Norway
	Recruitment setting/ location:	6 recruiting study centres in Denmark, 3 in Ireland, and 3 in Norway
Participants	*Participants:*	Healthy women aged 18–45 years requesting contraception and who had a history of regular, cyclic menstrual cycles (cycle length 21–35 days) without use of hormonal contraceptives, normal or clinically insignificant cervical smear not requiring further follow-up, having a successful levonorgestrel-releasing intrauterine system (LNG-IUS) placement (long acting progestin-releasing IUD)<br>*Exclusion criteria: H*ad not had at least two complete menstrual cycles after removal of a previous LNG-IUS; had a vaginal or caesarean delivery or abortion within 6 weeks of initial screening, had an infected abortion or postpartum endometritis within 90 days of screening, or had a history of abnormal bleeding, had any pelvic, genital, or gynecologic abnormalities, had any malignancy or severe hypertension, or had used long-acting contraceptive methods within 90 days of screening or had been indicated for treatment of idiopathic HMB.
	Total number:*	N=187 randomised; N=184 analysed, n=62 in TXA group, (n=62 in Mefenamic acid group,) n=60 in placebo group
	Age:	Mean age – TXA group: 31.5 +/- 7.2, placebo group: 33.1 +/- 7.0
Intervention	*Treatment dose, timing, duration:*	500 mg encapsulated TXA 3x/day from first day of a bleeding or spotting episode (however, if the bleeding or spotting episode started in the afternoon or evening, the women were instructed to start study drug

		treatment the morning of the **next day**) until bleeding or spotting stopped without restriction to treatment length; treatment repeated for each bleeding/spotting episode during 90 days post-IUD insertion
	Comparator:	Placebo capsules identical in appearance (same dose, timing, duration as TXA (and Mefenamic acid))
	Notes:	The first treatment course was to be started on the day after LNG-IUS placement and was to be restarted on occurrence of all bleeding or spotting episodes during the 90 days of study assessment followed by a 30-day follow-up period without oral study treatment.
Outcomes^	*Objective:*	n/a
	Subjective/ self-reported:	*Primary efficacy outcome:* Median number of bleeding / spotting days during the 90-day period post-LNG-IUS insertion;<br>*Secondary efficacy outcomes:* number of bleeding-only days, number of spotting-only days, number of bleeding / spotting episodes, and number of bleeding days with heavy intensity during 90- and 60-day period post-insertion; IUD satisfaction and continuation rate; TXA/Placebo treatment satisfaction; occurrence of dysmenorrhea; compliance;<br>Adverse events (summarised using MedDRA)
	Note:	
Other	*Funding:*	Funded by Bayer Healthcare, Berlin, Germany
	Ethics approval:	The protocol and all protocol amendments were reviewed and approved by each study site's institutional review board.
	Conflict of interest:	All co-authors were employed and/or received financial support from Bayer Healthcare to conduct this trial.
	Notes:	

Vermylen (1968)[19]

Methods	*Design (trial name):*	Crossover
	Trial dates/ duration:	Not reported
	Trial registration:	Not reported
Setting	*Country:*	Belgium
	Recruitment setting/ location:	Not specified
Participants	*Participants:*	Women with 'profuse' menorrhagia in whom no gynaecological or coagulation anomaly could be detected. (In 14 of 16 women, the obj. MBL test confirmed the diagnosis of HMB, as MBL was >80ml.)
	Total number:*	N=22 randomised; N=16 analysed
	Age:	Not reported
Intervention	*Treatment dose, timing, duration:*	6x0.5g TXA tablets per day from first day of menstruation until bleeding had completely stopped; 6 consecutive periods - 3 'active', 3 'placebo'
	Comparator:	Placebo (same dose, timing, duration as TXA)
	Notes:	n/a
Outcomes^	*Objective:*	Mean menstrual haemoglobin loss, using Perdometer method (measuring Hb content in collected menstrual pads, similar to AH method)
	Subjective/ self-reported:	Bleeding length (total duration across three menstrual periods in days); side effects, reported as total number of 'days' (not patients) with various types of side effects per group
	Note:	
Other	*Funding:*	TXA and placebo were supplied by AB Kabi, Stockholm.

	Ethics approval:	Not reported
	Conflict of interest:	Not reported
	Notes:	

Weström 1970[20]

Methods	*Design (trial name):*	RCT, 1:1 parallel
	Trial dates/ duration:	Not reported
	Trial registration:	Not reported
Setting	*Country:*	Sweden
	Recruitment setting/ location:	Not reported
Participants	*Participants:*	Women having contraceptive IUD inserted, including Lippes' loop B (one woman), Lippes' loop C (58 women), and Saf-T-Coil 33 S (six women). None of the participants had previously any gynaecologic disorders, including menstrual ones, and oral contraceptives had not been used for several menstrual cycles.
	Total number:*	N=65 for MBL outcome; N=55 for Hb outcome
	Age:	Age range 15-44 years (details provided in table I of the publication)
Intervention	*Treatment dose, timing, duration:*	1.5 g (3x 500 mg tablets) TXA 4x/day for 5 days (i.e. 6g total/day).<br>If necessary, followed by 1 g (2x 500mg tablets) TXA 3x a day for an additional 5 days (i.e. 3 g total/day), 3 cycles
	Comparator:	Placebo
	Notes:	Initial pre-insertion treatment free control cycle
Outcomes^	*Objective:*	Hb, MBL (AH method)
	Subjective/ self-reported:	Bleeding duration
	Note:	Women were instructed to take tablets and collect tampons and menstrual pads only during menstrual bleeding and not for more than ten days in each menstrual period; unexpectedly heavy flow led to difficulties for some women in 'catching' all the blood in the sanitary napkins and tampons, especially in the group of women receiving the placebos (24 menstruations in placebo group and 1 menstruation in TXA group).
Other	*Funding:*	The study was supported by the Ford Foundation. TXA and placebo tablets were supplied by AB Kabi, Stockholm, Sweden.
	Ethics approval:	Not reported
	Conflict of interest:	Not reported
	Notes:	

Ylikorkala (1983)[21]

Methods	*Design (trial name):*	Crossover
	Trial dates/ duration:	Not reported
	Trial registration:	Not reported
Setting	*Country:*	Finland
	Recruitment setting/ location:	Not reported
Participants	*Participants:*	Women with contraceptive IUD (copper IUD: n=18, inert IUD: n=1) inserted ≥ 1 year ago, who sought medical help because of HMB; among these n=15 had an objectively confirmed MBL >80 ml, and n=4 had MBL of 70-80 ml.
	Total number:*	N=21 initially included in trial, but only N=19 assessed/analysed
	Age:	Mean age of 35 years +/- 1.7 (SE); range 24-44 years
Intervention	*Treatment dose, timing, duration:*	1.5 g oral TXA 3x/day for 5 days from 1st day of menstruation, 2 cycles; [In another 2 cycles 50 mg Diclofenac Sodium (DS) 3x/day on the first day followed by 25 mg DS 3x/day for 4 days]
	Comparator:	Placebo (same dose and timing as TXA and DS), 1 cycle
	Notes:	Iron supplements were stopped at start of trial
Outcomes^	*Objective:*	Primary: Hb (but not reported by treatment group); MBL (AH method)
	Subjective/ self-reported:	Primary: Bleeding length; occurrence of intermenstrual bleeding; side effects; Secondary: 'restricted activity'; pelvic pain; subjective assessment of the general treatment effect (score of 1-4, 'no' to 'complete relief')
	Note:	
Other	*Funding:*	The authors thanked Margareta Schannong and J. Henriksson, KabiVitrum. and E. Woulijoki, Ciba-Geigy, for their help. Mölnlycke Co. donated the pads used in this study. The research was also supported by the Medical Research Council, Academy of Finland.
	Ethics approval:	Not reported
	Conflict of interest:	Not reported
	Note:	

* Total number of participants randomized (and number included in main analysis) relevant to this review; ^ Outcome categories (and assessment time points) included in review; ~ Other groups not included in this review

AMCA, trans-4-(aminomethyl)cyclohexanecarboxylic acid (standardised chemical name for tranexamic acid); AH method, alkaline hematin method to determine menstrual blood loss[6]; assignm., assignment; AUB, abnormal uterine bleeding; DMPA, Depo-medroxy progesterone acetate (a long acting progestin); g, gram; gr., group; gyn., gynaecological; Hb, haemoglobin; HMB, heavy menstrual bleeding; HR-QoL, health-related quality of life; ITT, intention-to-treat; Intrauterine Device, IUD; LLD-IUD, Lippes Loop D Intrauterine Device; MBL, menstrual blood loss; MIQ, Menorrhagia Impact Questionnaire; no., number; Norplant, six-capsule levonorgestrel subdermal implant (a long-acting progestine); OCP, oral contraceptive pill; OTBC method, Oxford total body counter method, 2-4 uCi ^{59}Fe given i.v. at baseline; blood loss estimated from loss of radioactivity x total blood volume after treatment period[3]; parallel, parallel-group trial; PBAC, pictorial blood loss assessment chart; Perdometer method, developed by Rustad 1963[22] to estimate blood loss by measuring HB content in menstrual pads, similar to AH method above. QoL, Quality of Life; q., question; RCT, randomized controlled trial; subj., subjective assessment method; tbl., tablet; TXA, tranexamic acid; VAS, visual analogue scale; vs, versus; w, with; w/o, without; wk(s), week(s); yr(s), year(s); DVT, Deep Vein Thrombosis; PE, Pulmonary Embolism

References:

1. Adawiyah R, Mulyantoro I, Dewantiningrum J, et al. Randomized Controlled Trial of Tranexamic Acid's Effect on Bleeding Length: A Study on DMPA Users with Abnormal Uterine Bleeding Who Receive Low-Dose Oral Contraceptive Pill. *2020* 2020:5. doi: 10.14710/jbtr.v6i1.4450 [published Online First: 2020-04-30]
2. Callender ST, Warner GT, Cope E. Treatment of menorrhagia with tranexamic acid. A double-blind trial. *British Medical Journal* 1970;4(5729):214-6.
3. Warner GT, Oliver R. A whole-body counter for clinical measurements utilizing the 'shadow shield' technique. *Phys Med Biol* 1966;11(1):83-94. doi: 10.1088/0031-9155/11/1/307 [published Online First: 1966/01/01]
4. Drosdal H. Treatment of menorrhagia with tranexamic acid. [Norwegian]. *Tidsskrift for den Norske Laegeforening* 1993;113(23):2907-10.
5. Edlund M, Andersson K, Rybo G, et al. Reduction of menstrual blood loss in women suffering from idiopathic menorrhagia with a novel antifibrinolytic drug (Kabi 2161). *British Journal of Obstetrics & Gynaecology* 1995;102(11):913-7.
6. Hallberg L, Nilsson L. Determination of menstrual blood loss *Scand J Clin Lab Invest* 1964;16:244-8. [published Online First: 1964/01/01]
7. Newton J, Barnard G, Collins W. A rapid method for measuring menstrual blood loss using automatic extraction. *Contraception* 1977;16(3):269-82. doi: https://doi.org/10.1016/0010-7824(77)90026-9
8. Freeman EW, Lukes A, VanDrie D, et al. A dose-response study of a novel, oral tranexamic formulation for heavy menstrual bleeding. *American Journal of Obstetrics and Gynecology* 2011;205(4):7. doi: 10.1016/j.ajog.2011.05.015
9. Hamulyák EN, Wiegers HMG, Hutten BA, et al. Heavy menstrual bleeding on direct factor Xa inhibitors: the MEDEA randomized clinical trial. *Res Pract Thromb Haemost* 2024;8(4):102448. doi: 10.1016/j.rpth.2024.102448 [published Online First: 2024/07/02]
10. Hamulyák EN, Wiegers HMG, Scheres LJJ, et al. Heavy menstrual bleeding on direct factor Xa inhibitors: Rationale and design of the MEDEA study. *Res Pract Thromb Haemost* 2021;5(1):223-30. doi: 10.1002/rth2.12471 [published Online First: 2021/02/05]
11. Lin X, Gao ES, Li D, et al. Preventive treatment of intrauterine device-induced menstrual blood loss with tranexamic acid in Chinese women. *Acta Obstetricia Et Gynecologica Scandinavica* 2007;86(9):1126-29. doi: 10.1080/00016340701516777
12. Lukes AS, Moore KA, Muse KN, et al. Tranexamic acid treatment for heavy menstrual bleeding: a randomized controlled trial. *Obstetrics and gynecology* 2010;116(4):865-75. doi: 10.1097/AOG.0b013e3181f20177
13. Nilsson L, Rybo G. Treatment of menorrhagia with an antifibrinolytic agent, Tranexamic acid (AMCA) - A double blind investigation. *Acta Obstetricia et Gynecologica Scandinavica* 1967;46:572-80.
14. Peterson K, Jelert H, Diernaes E, et al. [Treatment of hypermenorrhea with tranexamic acid] *Ugeskr Laeger* 1983;145(36):2759-60.
15. Phupong V, Sophonsritsuk A, Taneepanichskul S. The effect of tranexamic acid for treatment of irregular uterine bleeding secondary to Norplant use. *Contraception* 2006;73(3):253-6.
16. Rivera R, Wheeler RG, Balogh SA, et al. Sustained intrauterine release of AMCA to reduce menstrual blood loss. *Contraceptive Delivery Systems* 1984;5(2):109-15.
17. Senthong AJ, Taneepanichskul S. The effect of tranexamic acid for treatment irregular uterine bleeding secondary to DMPA use. *Journal of the Medical Association of Thailand* 2009;92(4):461-5.
18. Sordal T, Inki P, Draeby J, et al. Management of initial bleeding or spotting after levonorgestrel-releasing intrauterine system placement: a randomized controlled trial.

Obstetrics & Gynecology 2013;121(5):934-41. doi: https://dx.doi.org/10.1097/AOG.0b013e31828c65d8
19. Vermylen J, Verhaegen-Declercq ML, Verstraete M, et al. A double blind study of the effect of tranexamic acid in essential menorrhagia. *Thromb Diath Haemorrh* 1968;20(3):583-7.
20. Weström L, Lp B. Effect of tranexamic acid (AMCA) in menorrhagia with intrauterine contraceptive devices. *The Journal of reproductive medicine* 1970;5 4:154-61.
21. Ylikorkala O, Viinikka L. Comparison between antifibrinolytic and antiprostaglandin treatment in the reduction of increased menstrual blood loss in women with intrauterine contraceptive devices. *British Journal of Obstetrics & Gynaecology* 1983;90(1):78-83.
22. Rustad H. Measurement of blood-loss during surgery. *Lancet* 1963;1(7294):1304. doi: 10.1016/s0140-6736(63)91994-9 [published Online First: 1963/06/15]
