# Supplementary material for: Tranexamic acid for anemia in women of reproductive age: A systematic review of randomized trials
Source: PLoS One. 2026 Nov 6;21(11):e0362488. doi: 10.1371/journal.pone.0362488 (PMC13743017; doi:10.1371/journal.pone.0362488)
Supplement: Supplementary file 6 [file pone.0362488.s006.pdf]

Supplementary File 6 – Risk of bias assessments of included trials

(Berendes et al. (2026), PLOS ONE. DOI: 10.1371/journal.pone.0362488)

A – Risk of bias (ROB) summary by trial (Traffic light plot)

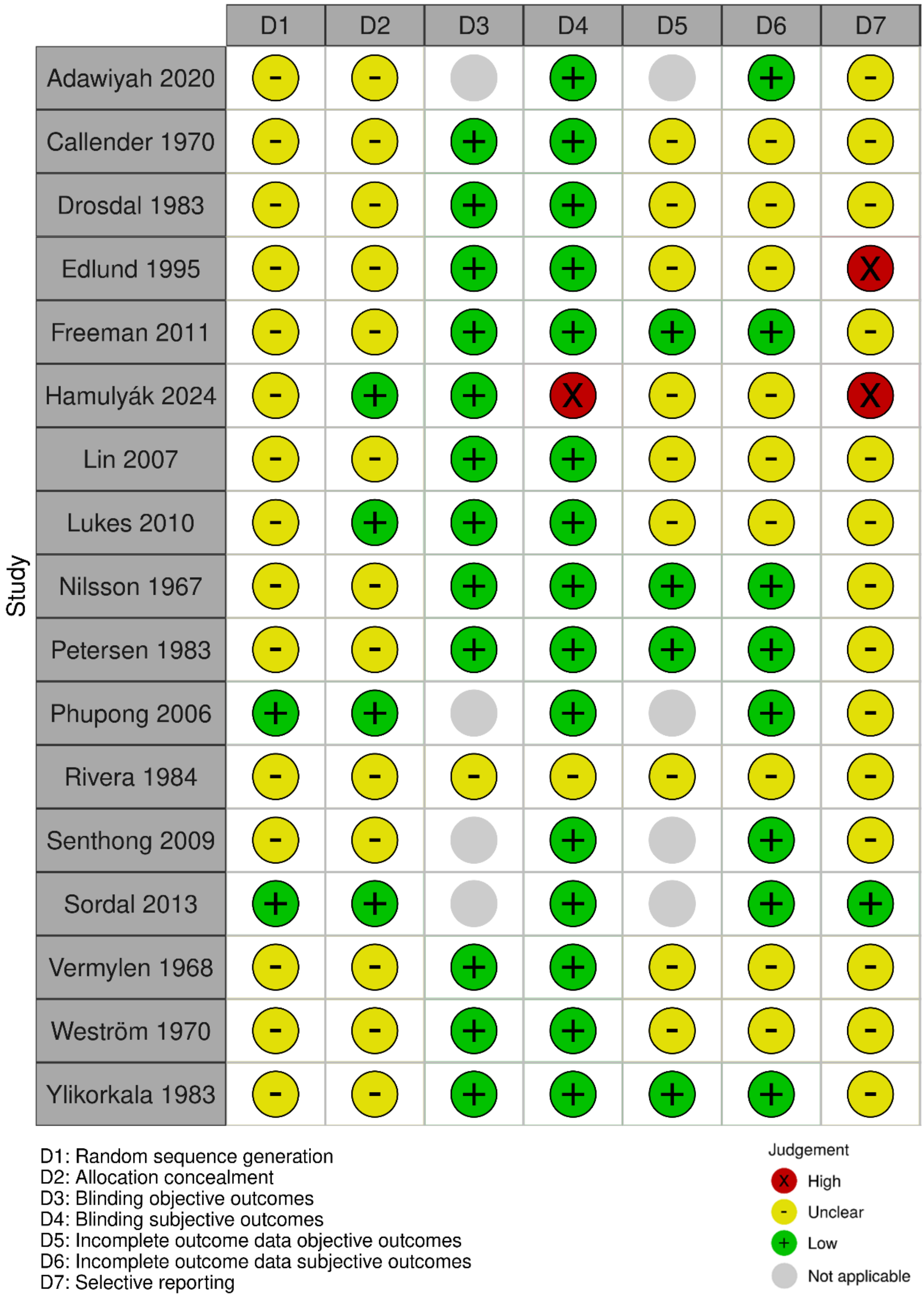

Source of assessment and visualization tools: Higgins et al. 2011[1]; McGuinness & Higgins 2020[2]; Web-tool URL (accessed 03/10/25):https://mcguinlu.shinyapps.io/robvis/_w_066364ea/

B - Detailed ROB assessment by study and by objective versus subjective outcome

Adawiyah (2020)[3]

Random sequence generation (selection bias)	*Judgement:* Unclear<br><br>*Support:* No information on sequence generation provided, mentions only "forty-four subjects in this study were divided with simple randomization into two groups".
Allocation concealment (selection bias)	*Judgement:* Unclear<br><br>*Support:* Insufficient information on allocation concealment provided, mentions only "The drugs for both groups were packaged in same colored capsules, we also asked people outside of author and co-author to choose a closed envelope containing which group the subjects would be assigned to."
Blinding, *subjective* outcomes (performance & detection bias)	Judgement: Low<br><br>*Support:* Described as "double-blind" and mentions that "drugs for both groups were packaged in same colored capsules" and that "Both authors and subjects were not aware of the administered drugs".
Incomplete outcome data, *subjective* outcomes (attrition bias)	*Judgement:* Low<br><br>*Support:* No post-randomisation exclusions or losses to follow-up reported - quote: "Until the end of the study no subject resigned, all of the 44 women fill the menstrual card as shown in the Consort Diagram of Figure 1."
Selective outcome reporting (reporting bias)	*Judgement:* Unclear<br><br>*Support:* No protocol available; no trial registration reported

Callender (1970)[4]

Random sequence generation (selection bias)	*Judgement:* Unclear<br><br>*Support:* No information on sequence generation provided, mentions only that the "order of treatment was randomized".
Allocation concealment (selection bias)	*Judgement:* Unclear<br><br>*Support:* Allocation concealment not described in sufficient detail, i.e. unclear if tablet bottles were sequentially numbered and/or remotely allocated – quote: "over the

	second three menstrual period each patient was given [...] a bottle containing 32 tablets labelled A [...] For the third three months she was similarly instructed but tablets A were replaced by tablets B." The tablets were either TXA or a placebo of "similar size and appearance. The order of treatment was randomized, and neither the patients nor those conducting the study knew the identity of tablets A and B."
Blinding, *objective* outcomes (performance & detection bias)	Judgement: Low<br><br>*Support:* Described as "double-blind" and specified that the "tablets were either tranexamic acid 0.5 g. per tablet or a placebo of similar size and appearance" and that "neither the patients nor those conducting the study knew the identity of tablets A and B."
Blinding, *subjective* outcomes (performance & detection bias)	Judgement: Low<br><br>*Support:* Same as above.
Incomplete outcome data, *objective* outcomes (attrition bias)	*Judgement:* Unclear<br><br>*Support:* Four of 20 participants excluded – quote: "Originally 20 patients agreed to take part in the investigation, but four did not complete the trial and are not included in the analysis-two elected to have a hysterectomy rather than continue with the trial, one was found to be losing blood from an undefined source between the menstruations, and one failed to return for follow-up."
Incomplete outcome data, *subjective* outcomes (attrition bias)	*Judgement:* Unclear<br><br>*Support:* Same as above.
Selective outcome reporting (reporting bias)	*Judgement:* Unclear<br><br>*Support:* No protocol available; no trial registration reported

Drosdal (1983)[5] [Norwegian]

Random sequence generation (selection bias)	*Judgement:* Unclear<br><br>*Support:* No information on sequence generation provided, mentions only "Randomisation was separate for each clinic".

Allocation concealment (selection bias)	*Judgement:* Unclear<br><br>*Support:* No information on allocation concealment provided.
Blinding, *objective* outcomes (performance & detection bias)	Judgement: Low<br><br>*Support:* Described as “double-blind”.
Blinding, *subjective* outcomes (performance & detection bias)	Judgement: Low<br><br>*Support:* Described as “double-blind”
Incomplete outcome data, *objective* outcomes (attrition bias)	*Judgement:* Unclear<br><br>*Support:* Reported only that 37 of 44 participants completed the study.
Incomplete outcome data, *subjective* outcomes (attrition bias)	*Judgement:* Unclear<br><br>*Support:* Reported only that 37 of 44 participants completed the study.
Selective outcome reporting (reporting bias)	*Judgement:* Unclear (for objective outcome), High (for subjective outcomes)<br><br>*Support:* Some of the subjective outcome data mentioned in the methods section were not reported in the results section; protocol not available; no trial registration reported

Edlund (1995)[6]

Random sequence generation (selection bias)	*Judgement:* Unclear<br><br>*Support:* No information on sequence generation provided, mentions only that "91 patients were randomised".
Allocation concealment (selection bias)	*Judgement:* Unclear<br><br>*Support:* No information on allocation concealment provided.
Blinding, *objective* outcomes (performance & detection bias)	Judgement: Low<br><br>*Support:* Described as “double-blind”, using “double dummy technique”.
Blinding, *subjective* outcomes (performance & detection bias)	Judgement: Low<br><br>*Support:* Described as “double-blind”, using “double dummy technique”.

Incomplete outcome data, *objective* outcomes (attrition bias)	*Judgement:* Unclear<br><br>*Support:* Outcome data from 68/ 91 (75%) or less participants were analysed. Attrition figures and reasons have not been given by treatment arm.
Incomplete outcome data, *subjective* outcomes (attrition bias)	*Judgement:* Unclear<br><br>*Support:* See comment above.
Selective outcome reporting (reporting bias)	*Judgement:* High<br><br>*Support:* Some outcomes not reported (Hb) or not reported in detail; Not prospectively registered and no protocol available.

Freeman (2011)[7]

Random sequence generation (selection bias)	*Judgement:* Unclear<br><br>*Support:* No information on sequence generation provided, mentions only that this was a "randomized" study.
Allocation concealment (selection bias)	*Judgement:* Unclear<br><br>*Support:* No information on allocation concealment provided.
Blinding, *objective* outcomes (performance & detection bias)	Judgement: Low<br><br>*Support:* Described as "double-blind" in publication and as "Quadruple (Participant, Care Provider, Investigator, Outcomes Assessor)" in trial registration.
Blinding, *subjective* outcomes (performance & detection bias)	Judgement: Low<br><br>*Support:* Described as "double-blind"
Incomplete outcome data, *objective* outcomes (attrition bias)	*Judgement:* Low<br><br>*Support:* 304 people were randomised, with relatively low lost to follow-up rates described in PRISMA chart (Figure 1). 294/304 (96.7%) participants were included in modified ITT efficacy analysis of MBL.

Incomplete outcome data, *subjective* outcomes (attrition bias)	*Judgement:* Low<br><br>*Support:* 297/304 (97.7%) participants were included in safety analysis.
Selective outcome reporting (reporting bias)	*Judgement:* Unclear<br><br>*Support:* Protocol not available. Trial registration (NCT00401193) reported; Outcomes were pre-specified before trial completion but described vaguely (primary: "Efficacy of XP12B to reduce menstrual blood loss"; secondary: "Safety and patient reported outcome improvements"). Detailed outcome definitions were only provided at study end, when reporting results.

Hamulyák (2024)[8 9]

Random sequence generation (selection bias)	*Judgement:* Unclear<br><br>*Support:* No information on sequence generation is provided in the publication of results. A 2020 publication describing the trial rationale does not specifically address sequence generation, and only states: "The randomization process is centralized through a web-based Good Clinical Practice approved tool, using random permuted blocks of varying size. "
Allocation concealment (selection bias)	*Judgement:* Low<br><br>*Support:* No information on allocation concealment in the results publication. However, the 2020 rationale paper states that randomization was centralized via a web-based GCP-approved tool, which is likely to have ensured adequate concealment.
Blinding, *objective* outcomes (performance & detection bias)	Judgement: Low<br><br>*Support:* The study is described as an "open-label, pragmatic clinical trial". However, the 2020 rationale paper states: "New symptomatic thromboembolic events (either venous or arterial) objectively confirmed by imaging will be recorded. All suspected clinical outcomes during the study period will be adjudicated by a committee blinded for treatment allocation". Therefore, for objective safety outcomes such as thromboembolic events confirmed by imaging and for the study's secondary outcome Hb (which was assessed but not reported), the risk of performance and detection bias is considered low.

Blinding, *subjective* outcomes (performance & detection bias)	Judgement: High<br><br>*Support:* The study is described as an "open-label, pragmatic clinical trial," with no indication of blinding for participant-reported subjective outcomes.
Incomplete outcome data, *objective* outcomes (attrition bias)	*Judgement:* Unclear<br><br>*Support:* The report stated that 16 participants were randomised across three groups but does not provide group-specific numbers. It also notes: "Two women did not have any PBAC measurements after randomization; 1 woman in the dabigatran group stopped study participation and 1 woman in the continue FXa inhibitor without intervention group did not have any menstrual period after randomization."
Incomplete outcome data, *subjective* outcomes (attrition bias)	*Judgement:* Unclear<br><br>*Support:* See comment above.
Selective outcome reporting (reporting bias)	*Judgement:* High<br><br>*Support:* The 2020 rationale paper and trial registration list several outcomes, only some of which are reported in the results publication. Although the results were published in a 'Research Letter' format due to premature trial termination from low recruitment, this does not fully account for the selective reporting of outcomes.

Lin (2007)[10]

Random sequence generation (selection bias)	*Judgement:* Unclear<br><br>*Support:* No information on sequence generation is provided. The report states only that participants were "randomly allocated" to treatment groups, and that "some of the subjects were further randomly sampled" for objective outcome assessments, without specifying the randomisation method.
Allocation concealment (selection bias)	*Judgement:* Unclear<br><br>*Support:* No information on allocation concealment provided.
Blinding, *objective* outcomes (performance & detection bias)	Judgement: Low

	Support: Described as “double-blind”
Blinding, *subjective* outcomes (performance & detection bias)	Judgement: Low<br><br>*Support:* Described as “double-blind”
Incomplete outcome data, *objective* outcomes (attrition bias)	*Judgement:* Unclear<br><br>*Support:* The report states that “some of the subjects were further randomly sampled” for the assessment of objective outcomes but does not specify how many. It mentions that objective outcome data were available for 54 participants among 64 included in a modified ITT analysis, but the total number randomized for objective outcome assessment is unclear. No information is provided on reasons for missing objective data or whether attrition differed by group.
Incomplete outcome data, *subjective* outcomes (attrition bias)	*Judgement:* Unclear<br><br>*Support:* Based on the results section, 195 participants were randomised, 175 were included in a modified ITT analysis and subjective outcome data appear to be available for a total of 156/195 (80%) participants. The report states: “A total of 252 eligible women were invited into the study, but 57 refused to participate. Two subjects discontinued the use of IUD and 18 dropped out during the first treatment cycle. Finally, 175 subjects were included in the ITT population and 156 in the PP population”. No further information is provided on reasons for missing data or whether attrition differed by group.
Selective outcome reporting (reporting bias)	*Judgement:* Unclear<br><br>*Support:* Protocol not available; no trial registration reported

Lukes (2010)[11]

Random sequence generation (selection bias)	*Judgement:* Unclear<br><br>*Support:* No information on how the random sequence was generated. Report mentions only: "A randomisation schedule was generated for packaging, labeling, and treatment group assignment using a permuted block randomization scheme. The randomization block size was eight, with five participants assigned to tranexamic acid and three participants assigned to placebo per block."
Allocation concealment	*Judgement:* Low

(selection bias)	Support: Central allocation - Study drug was individually packaged for each visit and labeled with the study code. Quote: “Fisher Clinical Services was responsible for generating the randomization and allocation sequence and enrolling and assigning blinded study medication to individuals. Participants, investigators, sponsor, statisticians, clinical data management staff, and clinical monitors were blinded to study group allocation. Study drug was individually packaged for each visit and labeled with<br>the study code. The tranexamic acid and placebo tablets were identical in appearance.”
Blinding, *objective* outcomes (performance & detection bias)	Judgement: Low<br><br>*Support:* States that “participants, investigators, sponsor, statisticians, clinical data management staff, and clinical monitors were blinded to study group allocation” and that the “tranexamic acid and placebo tablets were identical in appearance”.
Blinding, *subjective* outcomes (performance & detection bias)	Judgement: Low<br><br>*Support:* See comment above
Incomplete outcome data, *objective* outcomes (attrition bias)	*Judgement:* Unclear<br><br>*Support:* A total of 196 people were randomised, among whom 148 (75.5%) participants completed the study, including 94/123 (76%) participants in the TXA arm and 54/73 (74%) in the Placebo arm. The modified ITT analysis included 115/123 participants in the TXA arm and 72/73 in the placebo arm. The report provides only limited information on the reasons for withdrawals/ exclusions/ lost-to-follow up per treatment arm – quote: “Six participants in the tranexamic acid group and 1 in the placebo group were excluded from the intent-to-treat analyses because they did not ingest at least one dose of study drug. The modified intent-to-treat population consisted of 187 women, 115 in the tranexamic acid group and 72 in the placebo group. Two individuals in the tranexamic acid group were excluded from the modified intent-to-treat analyses because posttreatment data were unavailable. In all, 148 women completed the study, 94 in the tranexamic acid group and 54 in the placebo group. Forty-eight participants withdrew from the study; the majority were lost to follow-up (16 individuals). Two participants in the placebo group and no individuals in the tranexamic acid group discontinued because of unsatisfactory efficacy response.”

Incomplete outcome data, *subjective* outcomes (attrition bias)	*Judgement:* Unclear<br><br>*Support:* Protocol not available. Trial registration (NCT00386308) reported; Outcomes were pre-specified before trial completion but described vaguely -primary: "Efficacy of XP12B to reduce menstrual blood loss in women with objective evidence of heavy menstrual bleeding."); The following details were only provided at study end, when reporting results "Mean Reduction From Baseline in Menstrual Blood Loss (MBL) [Time Frame: Baseline MBL over 6 menstrual cycles] - reduction of menstrual blood loss in mL".<br>The publication refers to results from the 1st, 2nd, 3rd and 6th cycle: "The change in menstrual blood loss was calculated by subtracting the mean blood loss during the two pretreatment cycles from the mean blood loss during four of the on-treatment cycles (first, second, third, and sixth cycles)."<br>The publication also reports Hb and anaemia outcomes that had not been pre-specified.
Selective outcome reporting (reporting bias)	*Judgement:* Unclear<br><br>*Support:* Protocol not available. Trial registration (NCT00386308) reported; Subjective outcomes were pre-specified before trial completion but described vaguely (secondary: "Safety and patient reported outcome improvements"). Detailed outcome definitions were only provided at study end, when reporting results.
Other / comment	Concerns regarding imbalances between groups at baseline, including more women had anaemia in TXA arm (34%) than placebo arm (18%) and mean MBL was higher in TXA arm (172.3+/- 95.6) than placebo arm (153+/-66.6) at baseline.

Nilsson (1967)[12]

Random sequence generation (selection bias)	*Judgement:* Unclear<br><br>*Support:* No information on sequence generation provided, mentions only that "each patient received tablets containing placebo, 0.25 g, and 0.5 g of AMCA allocated to random distribution as in a double blind trial."
Allocation concealment (selection bias)	*Judgement:* Unclear

	Support: Insufficient information on allocation concealment provided, mentions only "each patient received a preparation marked respectively I, II and III for the first, second, and third of these cycles. One of the preparations contained a placebo, and the other 2 contained AMCA [...] random distribution as in a double blind trial."
Blinding, *objective* outcomes (performance & detection bias)	Judgement: Low<br><br>*Support:* Described as "double-blind"
Blinding, *subjective* outcomes (performance & detection bias)	Judgement: Low<br><br>*Support:* Described as "double-blind"
Incomplete outcome data, *objective* outcomes (attrition bias)	*Judgement:* Low<br><br>*Support:* The study does not report any exclusions or losses to follow-up after randomisation. It is therefore assumed that the reported objective outcome data (menstrual blood loss) were obtained from all 36 women enrolled in the trial.
Incomplete outcome data, *subjective* outcomes (attrition bias)	*Judgement:* Low<br><br>*Support:* The study does not report any exclusions or losses to follow-up after randomisation. It is therefore assumed that the reported subjective outcome data (side effects) were obtained from all 36 women enrolled in the trial.
Selective outcome reporting (reporting bias)	*Judgement:* Unclear<br><br>*Support:* Protocol not available; no trial registration reported
Other / comment	Change of intervention (TXA dosage) during the trial - quote: "When the first 8 patients participating in the trial had been treated, the results for patients who did not belong to the present series indicated that, at any rate, the dosage of 6 grams was too low. Consequently, the dose was increased to 2 tablets 6 times daily for 4 days, starting from patient no. 9. This amounted to a total dose of either 12 or 24 grams of AMCA. Because of this change in the dosage statistical analysis was made both for the entire series including the first 8 patients, and also for that part of the series in which the lower and the higher dosages of AMCA amounted to 12 and 24 grams respectively."

Petersen (1983)[13]

Random sequence generation (selection bias)	*Judgement:* Unclear<br><br>*Support:* No information on sequence generation provided, mentions only "randomised".
Allocation concealment (selection bias)	*Judgement:* Unclear<br><br>*Support:* No information on allocation concealment provided.
Blinding, *objective* outcomes (performance & detection bias)	Judgement: Low<br><br>*Support:* Described as "double-blind"
Blinding, *subjective* outcomes (performance & detection bias)	Judgement: Low<br><br>*Support:* Described as "double-blind"
Incomplete outcome data, *objective* outcomes (attrition bias)	*Judgement:* Low<br><br>*Support:* No missing data described.
Incomplete outcome data, *subjective* outcomes (attrition bias)	*Judgement:* Low<br><br>*Support:* No missing outcome data described.
Selective outcome reporting (reporting bias)	*Judgement:* Unclear<br><br>*Support:* Protocol not available; no trial registration reported

Phupong (2006)[14]

Random sequence generation (selection bias)	*Judgement:* Low<br><br>*Support:* Quote: "The subjects were assigned by a random number table".
Allocation concealment (selection bias)	*Judgement:* Low<br><br>*Support:* Quote: "The active capsules and the corresponding placebo capsules were packed in opaque sealed envelopes, and the envelopes were labeled sequentially with the subject number in the center (1–68). Each envelope was then distributed in a sequential numerical order to ensure randomization and double blindness to treatment. The research nurse prepared the envelopes. The treatment was not identified on the clinical form but rather by code numbers, which were revealed only at the end of the study."

Blinding, *subjective* outcomes (performance & detection bias)	Judgement: Low<br><br>*Support:* Described as “double-blind”
Incomplete outcome data, *subjective* outcomes (attrition bias)	*Judgement:* Low<br><br>*Support:* No missing outcome data described.
Selective outcome reporting (reporting bias)	*Judgement:* Unclear<br><br>*Support:* Protocol not available; no trial registration reported

Rivera (1984)[15]

Random sequence generation (selection bias)	*Judgement:* Unclear<br><br>*Support:* No information on sequence generation provided, mentions only "random allocation".
Allocation concealment (selection bias)	*Judgement:* Unclear<br><br>*Support:* No information on allocation concealment provided.
Blinding, *objective* outcomes (performance & detection bias)	Judgement: Unclear<br><br>*Support:* Insufficient information on blinding, the report mentions only that the ”control LLDs had the same physical alterations as the AMCA-loaded devices except that the reservoirs were empty”.
Blinding, *subjective* outcomes (performance & detection bias)	Judgement: Unclear<br><br>*Support:* Insufficient information on blinding, the report mentions only that the ”control LLDs had the same physical alterations as the AMCA-loaded devices except that the reservoirs were empty”.
Incomplete outcome data, *objective* outcomes (attrition bias)	*Judgement:* Unclear<br><br>*Support:* The study reports that a total of 21 participants were randomised, with outcome data available for 18 participants (86%) at 1 month follow-up (FU), 17 participants (81%) at 3 months FU and 13 participants (62%) at 6 months FU. It is unclear whether an ITT analysis was completed. The report mentions that one woman in each group became pregnant and that two women in the TXA group had IDU expulsions. No information is provided

	regarding the additional missing outcome data.
Incomplete outcome data, *subjective* outcomes (attrition bias)	*Judgement:* Unclear<br><br>*Support:* See comment above.
Selective outcome reporting (reporting bias)	*Judgement:* Unclear<br><br>*Support:* Protocol not available; no trial registration reported

Senthong (2009)[16]

Random sequence generation (selection bias)	*Judgement:* Unclear<br><br>*Support:* No information on sequence generation provided, mentions only "randomized".
Allocation concealment (selection bias)	*Judgement:* Unclear<br><br>*Support:* Not information on allocation concealment provided.
Blinding, *subjective* outcomes (performance & detection bias)	Judgement: Low<br><br>*Support:* Described as “double-blind”
Incomplete outcome data, *subjective* outcomes (attrition bias)	*Judgement:* Low<br><br>*Support:* Only one patient in the placebo group was lost to follow-up.
Selective outcome reporting (reporting bias)	*Judgement:* Unclear<br><br>*Support:* Protocol not available; no trial registration reported

Sordal (2013)[17]

Random sequence generation (selection bias)	*Judgement:* Low<br><br>*Support:* Use of "SAS-generated randomization list"
Allocation concealment (selection bias)	*Judgement:* Low<br><br>*Support:* Likely sequentially numbered drug containers of identical appearance, as the report states that participants were randomized to "treatment with encapsulated (all study drug capsules were identical in appearance to maintain blinding) oral tranexamic acid (500 mg), mefenamic acid (500 mg), or placebo (lactose and magnesium stearate, encapsulated to appear identical to study treatments)" and that "randomization was performed in a sequential manner in blocks of six using a SAS-generated randomization list prepared by the study sponsor."
Blinding, *subjective* outcomes (performance & detection bias)	Judgement: Low<br><br>*Support:* Described as "double-blind"
Incomplete outcome data, *subjective* outcomes (attrition bias)	*Judgement:* Low<br><br>*Support:* A total of 187 participants were randomised to three treatment arms with a total of 184 participants included in the ITT analysis; 56/ 63 (89%) participants randomised to the TXA arm completed the follow-up period, and 56/ 61 (92%) participants in the placebo arm.
Selective outcome reporting (reporting bias)	*Judgement:* Low<br><br>*Support:* Trial registration (NCT01295294) reported; all of the study's prespecified outcomes that are of interest in the review have been reported in the prespecified way.

Vermylen (1968)[18]

Random sequence generation (selection bias)	*Judgement:* Unclear<br><br>*Support:* No information on sequence generation provided, mentions only "the sequency of active and placebo bottles differred for each patient; the code was unknown both to the patient and to the person performing the blood-loss measurements."
Allocation concealment (selection bias)	*Judgement:* Unclear

	Support: Not information on allocation concealment provided.
Blinding, *objective* outcomes (performance & detection bias)	Judgement: Low<br><br>*Support:* The report states that the study was performed according to “double-blind technique“.
Blinding, *subjective* outcomes (performance & detection bias)	Judgement: Low<br><br>*Support:* The report states that the study was performed according to “double-blind technique“.
Incomplete outcome data, *objective* outcomes (attrition bias)	*Judgement:* Unclear<br><br>*Support:* Outcome data from 16/ 22 (73%) participants analysed.
Incomplete outcome data, *subjective* outcomes (attrition bias)	*Judgement:* Unclear<br><br>*Support:* Outcome data from 16/ 22 (73%) participants analysed.
Selective outcome reporting (reporting bias)	*Judgement:* Unclear<br><br>*Support:* Protocol not available; no trial registration reported

Weström 1970[19]

Random sequence generation (selection bias)	*Judgement:* Unclear<br><br>*Support:* No information on sequence generation provided, mentions only that tranexamic acid or a placebo were "dispensed randomly".
Allocation concealment (selection bias)	*Judgement:* Unclear<br><br>*Support:* The report states that investigators and participants had no access to the allocation code. However, it does not describe how allocation was concealed at the point of assignment. It is unclear whether the person enrolling participants could have predicted the next allocation.
Blinding, *objective* outcomes (performance & detection bias)	Judgement: Low<br><br>*Support:* Described as “double-blind” and states that “neither the participating women nor the investigators had access to the code”.

Blinding, *subjective* outcomes (performance & detection bias)	Judgement: Low<br><br>*Support:* Described as "double-blind" and states that "neither the participating women nor the investigators had access to the code".
Incomplete outcome data, *objective* outcomes (attrition bias)	*Judgement:* Unclear<br><br>*Support:* A total of 65 women were randomized, of whom 60 completed the study and contributed to the menstrual blood loss outcome data. Haemoglobin outcome data were available for 55 / 65 (85%) participants. The study reports reasons for some missing data, including one participant in the TXA arm, whose IDU-related menstrual bleeding ceased when she started treatment. It is unclear whether other losses to follow-up in the TXA group may have been related to a perceived lack of need for further treatment due to reduced bleeding. Additionally, the study reports that a temporary spectrophotometer malfunction affected four MBL determinations. Given the incomplete reporting of reasons for missing data and the potential for differential attrition, the risk of bias is judged as unclear.
Incomplete outcome data, *subjective* outcomes (attrition bias)	*Judgement:* Unclear<br><br>*Support:* See comment above.
Selective outcome reporting (reporting bias)	*Judgement:* Unclear<br><br>*Support:* Protocol not available; no trial registration reported

Ylikorkala (1983)[20]

Random sequence generation (selection bias)	*Judgement:* Unclear<br><br>*Support:* No information on sequence generation provided, mentions only that " treatments were given in a randomized order".
Allocation concealment (selection bias)	*Judgement:* Unclear<br><br>*Support:* Not information on allocation concealment provided, mentions only: "Because the TA and DS tablets were different in size and shape, the patients were always given the DS placebo together with the active TA tablets and vice versa to achieve the double-blind nature of the trial."

Blinding, *objective* outcomes (performance & detection bias)	Judgement: Low<br><br>*Support:* Described as "double-blind"
Blinding, *subjective* outcomes (performance & detection bias)	Judgement: Low<br><br>*Support:* Described as "double-blind"
Incomplete outcome data, *objective* outcomes (attrition bias)	*Judgement:* Low<br><br>*Support:* Outcome data from 19/ 21 (90%) participants were analysed.
Incomplete outcome data, *subjective* outcomes (attrition bias)	*Judgement:* Low<br><br>*Support:* Outcome data from 19/ 21 (90%) participants were analysed.
Selective outcome reporting (reporting bias)	*Judgement:* Unclear<br><br>*Support:* Protocol not available; no trial registration reported

References:

1. Higgins JP, Altman DG, Gotzsche PC, et al. The Cochrane Collaboration's tool for assessing risk of bias in randomised trials. *BMJ* 2011;343:d5928. doi: 10.1136/bmj.d5928 [published Online First: 2011/10/20]
2. McGuinness LA, Higgins JPT. Risk-of-bias VISualization (robvis): An R package and Shiny web app for visualizing risk-of-bias assessments. *Research Synthesis Methods* 2020;n/a(n/a) doi: 10.1002/jrsm.1411
3. Adawiyah R, Mulyantoro I, Dewantiningrum J, et al. Randomized Controlled Trial of Tranexamic Acid's Effect on Bleeding Length: A Study on DMPA Users with Abnormal Uterine Bleeding Who Receive Low-Dose Oral Contraceptive Pill. *2020* 2020:5. doi: 10.14710/jbtr.v6i1.4450 [published Online First: 2020-04-30]
4. Callender ST, Warner GT, Cope E. Treatment of menorrhagia with tranexamic acid. A double-blind trial. *British Medical Journal* 1970;4(5729):214-6.
5. Drosdal H. Treatment of menorrhagia with tranexamic acid. [Norwegian]. *Tidsskrift for den Norske Laegeforening* 1993;113(23):2907-10.
6. Edlund M, Andersson K, Rybo G, et al. Reduction of menstrual blood loss in women suffering from idiopathic menorrhagia with a novel antifibrinolytic drug (Kabi 2161). *British Journal of Obstetrics & Gynaecology* 1995;102(11):913-7.
7. Freeman EW, Lukes A, VanDrie D, et al. A dose-response study of a novel, oral tranexamic formulation for heavy menstrual bleeding. *American Journal of Obstetrics and Gynecology* 2011;205(4):7. doi: 10.1016/j.ajog.2011.05.015
8. Hamulyák EN, Wiegers HMG, Hutten BA, et al. Heavy menstrual bleeding on direct factor Xa inhibitors: the MEDEA randomized clinical trial. *Res Pract Thromb Haemost* 2024;8(4):102448. doi: 10.1016/j.rpth.2024.102448 [published Online First: 2024/07/02]

9. Hamulyák EN, Wiegers HMG, Scheres LJJ, et al. Heavy menstrual bleeding on direct factor Xa inhibitors: Rationale and design of the MEDEA study. *Res Pract Thromb Haemost* 2021;5(1):223-30. doi: 10.1002/rth2.12471 [published Online First: 2021/02/05]
10. Lin X, Gao ES, Li D, et al. Preventive treatment of intrauterine device-induced menstrual blood loss with tranexamic acid in Chinese women. *Acta Obstetricia Et Gynecologica Scandinavica* 2007;86(9):1126-29. doi: 10.1080/00016340701516777
11. Lukes AS, Moore KA, Muse KN, et al. Tranexamic acid treatment for heavy menstrual bleeding: a randomized controlled trial. *Obstetrics and gynecology* 2010;116(4):865-75. doi: 10.1097/AOG.0b013e3181f20177
12. Nilsson L, Rybo G. Treatment of menorrhagia with an antifibrinolytic agent, Tranexamic acid (AMCA) - A double blind investigation. *Acta Obstetricia et Gynecologica Scandinavica* 1967;46:572-80.
13. Peterson K, Jelert H, Diernaes E, et al. [Treatment of hypermenorrhea with tranexamic acid] *Ugeskr Laeger* 1983;145(36):2759-60.
14. Phupong V, Sophonsritsuk A, Taneepanichskul S. The effect of tranexamic acid for treatment of irregular uterine bleeding secondary to Norplant use. *Contraception* 2006;73(3):253-6.
15. Rivera R, Wheeler RG, Balogh SA, et al. Sustained intrauterine release of AMCA to reduce menstrual blood loss. *Contraceptive Delivery Systems* 1984;5(2):109-15.
16. Senthong AJ, Taneepanichskul S. The effect of tranexamic acid for treatment irregular uterine bleeding secondary to DMPA use. *Journal of the Medical Association of Thailand* 2009;92(4):461-5.
17. Sordal T, Inki P, Draeby J, et al. Management of initial bleeding or spotting after levonorgestrel-releasing intrauterine system placement: a randomized controlled trial. *Obstetrics & Gynecology* 2013;121(5):934-41. doi: https://dx.doi.org/10.1097/AOG.0b013e31828c65d8
18. Vermylen J, Verhaegen-Declercq ML, Verstraete M, et al. A double blind study of the effect of tranexamic acid in essential menorrhagia. *Thromb Diath Haemorrh* 1968;20(3):583-7.
19. Weström L, Lp B. Effect of tranexamic acid (AMCA) in menorrhagia with intrauterine contraceptive devices. *The Journal of reproductive medicine* 1970;5 4:154-61.
20. Ylikorkala O, Viinikka L. Comparison between antifibrinolytic and antiprostaglandin treatment in the reduction of increased menstrual blood loss in women with intrauterine contraceptive devices. *British Journal of Obstetrics & Gynaecology* 1983;90(1):78-83.
