# Supplementary material for: Tranexamic acid for anemia in women of reproductive age: A systematic review of randomized trials
Source: PLoS One. 2026 Nov 6;21(11):e0362488. doi: 10.1371/journal.pone.0362488 (PMC13743017; doi:10.1371/journal.pone.0362488)

Supplementary File 7 – Differences in baseline variables (for trials included in meta-analyses of objectively measured MBL outcomes)

(Berendes et al. (2026), PLOS ONE. DOI: 10.1371/journal.pone.0362488)

Forest plot S7A - Mean difference in baseline age (years)

	TXA			Control				Mean Difference
**Study or Subgroup**	**Mean**	**SD**	**Total**	**Mean**	**SD**	**Total**	**Weight**	**IV, Fixed, 95% CI**
**1.1.1 Women with HMB**								
Callender 1970	39.8	5.05	10	40.33	5.68	6	4.1%	-0.53 [-6.05, 4.99]
Freeman 2011	39.7	6.01	230	38.9	6	67	46.6%	0.80 [-0.83, 2.43]
Lukes 2010	38.7	6.4	115	38.7	6.8	72	32.4%	0.00 [-1.96, 1.96]
**Subtotal (95% CI)**			**355**			**145**	**83.1%**	**0.42 [-0.80, 1.65]**
Heterogeneity: Chi² = 0.50, df = 2 (P = 0.78); I² = 0%								
Test for overall effect: Z = 0.68 (P = 0.50)								
**1.1.2 Women with IUD**								
Rivera 1984	28.2	7.7	10	27.5	7.7	11	2.9%	0.70 [-5.89, 7.29]
Westrom 1970	30.2	6.4	34	28.2	5.84	31	14.0%	2.00 [-0.98, 4.98]
**Subtotal (95% CI)**			**44**			**42**	**16.9%**	**1.78 [-0.93, 4.49]**
Heterogeneity: Chi² = 0.12, df = 1 (P = 0.72); I² = 0%								
Test for overall effect: Z = 1.29 (P = 0.20)								
**Total (95% CI)**			**399**			**187**	**100.0%**	**0.65 [-0.46, 1.77]**
Heterogeneity: Chi² = 1.42, df = 4 (P = 0.84); I² = 0%								
Test for overall effect: Z = 1.15 (P = 0.25)								
Test for subgroup differences: Chi² = 0.80, df = 1 (P = 0.37), I² = 0%								

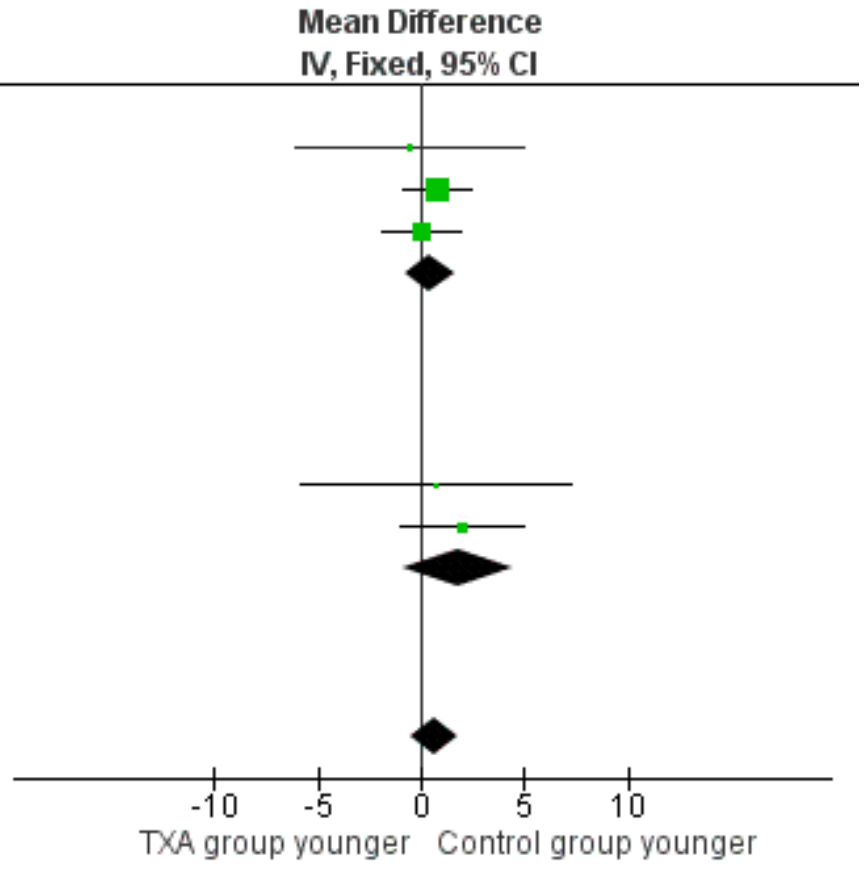

Forest plot S7B - Mean difference in objective baseline MBL (ml)

	Experimental			Control				Mean Difference
**Study or Subgroup**	**Mean**	**SD**	**Total**	**Mean**	**SD**	**Total**	**Weight**	**IV, Fixed, 95% CI**
Callender 1970	192.4	95.73	10	205.8	98.29	6	2.5%	-13.40 [-111.92, 85.12]
Edlund 1995	251.3	139.4	53	242.5	42.13	14	13.0%	8.80 [-34.74, 52.34]
Freeman 2011	173.5	98.29	230	153.6	98.29	67	34.5%	19.90 [-6.84, 46.64]
Lukes 2010	172.3	95.6	117	153	66.6	72	46.0%	19.30 [-3.87, 42.47]
Petersen 1983	211.4	91.84	8	166.9	74.88	9	3.8%	44.50 [-35.77, 124.77]
**Total (95% CI)**			**418**			**168**	**100.0%**	**18.27 [2.55, 33.99]**
Heterogeneity: Chi² = 1.01, df = 4 (P = 0.91); I² = 0%								
Test for overall effect: Z = 2.28 (P = 0.02)								

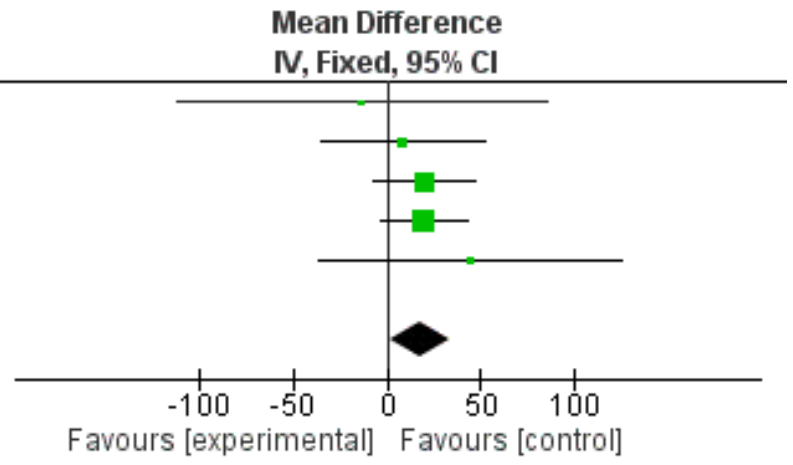

For comparison:

Copy of Figure 2. Results of trials assessing the effect of TXA on objective MBL (ml)

**Study or Subgroup**	**Mean Difference**	**SE**	**Weight**	**Mean Difference IV, Fixed, 95% CI**
**1.1.1 Women with HMB**				
Callender 1970	-62.38	26.33	1.9%	-62.38 [-113.99, -10.77]
Edlund 1995	-88.36	35.9465	1.0%	-88.36 [-158.81, -17.91]
Freeman 2011	-52.76	13.37	7.4%	-52.76 [-78.96, -26.56]
Lukes 2010	-30	12.5686	8.4%	-30.00 [-54.63, -5.37]
Nilsson 1967	-44.26	6.53	31.2%	-44.26 [-57.06, -31.46]
Petersen 1983	-68.88	15.98	5.2%	-68.88 [-100.20, -37.56]
**Subtotal (95% CI)**			**55.2%**	**-47.01 [-56.63, -37.39]**
Heterogeneity: Chi² = 5.73, df = 5 (P = 0.33); I² = 13%				
Test for overall effect: Z = 9.58 (P < 0.00001)				
**1.1.2 Women with IUD**				
Rivera 1984	-29.3	14.4763	6.3%	-29.30 [-57.67, -0.93]
Westrom 1970	-37.4	6.5385	31.1%	-37.40 [-50.22, -24.58]
Ylikorkala 1983	-69.71	13.39	7.4%	-69.71 [-95.95, -43.47]
**Subtotal (95% CI)**			**44.8%**	**-41.60 [-52.27, -30.93]**
Heterogeneity: Chi² = 5.54, df = 2 (P = 0.06); I² = 64%				
Test for overall effect: Z = 7.64 (P < 0.00001)				
**Total (95% CI)**			**100.0%**	**-44.58 [-51.72, -37.43]**
Heterogeneity: Chi² = 11.82, df = 8 (P = 0.16); I² = 32%				
Test for overall effect: Z = 12.23 (P < 0.00001)				
Test for subgroup differences: Chi² = 0.54, df = 1 (P = 0.46), I² = 0%				

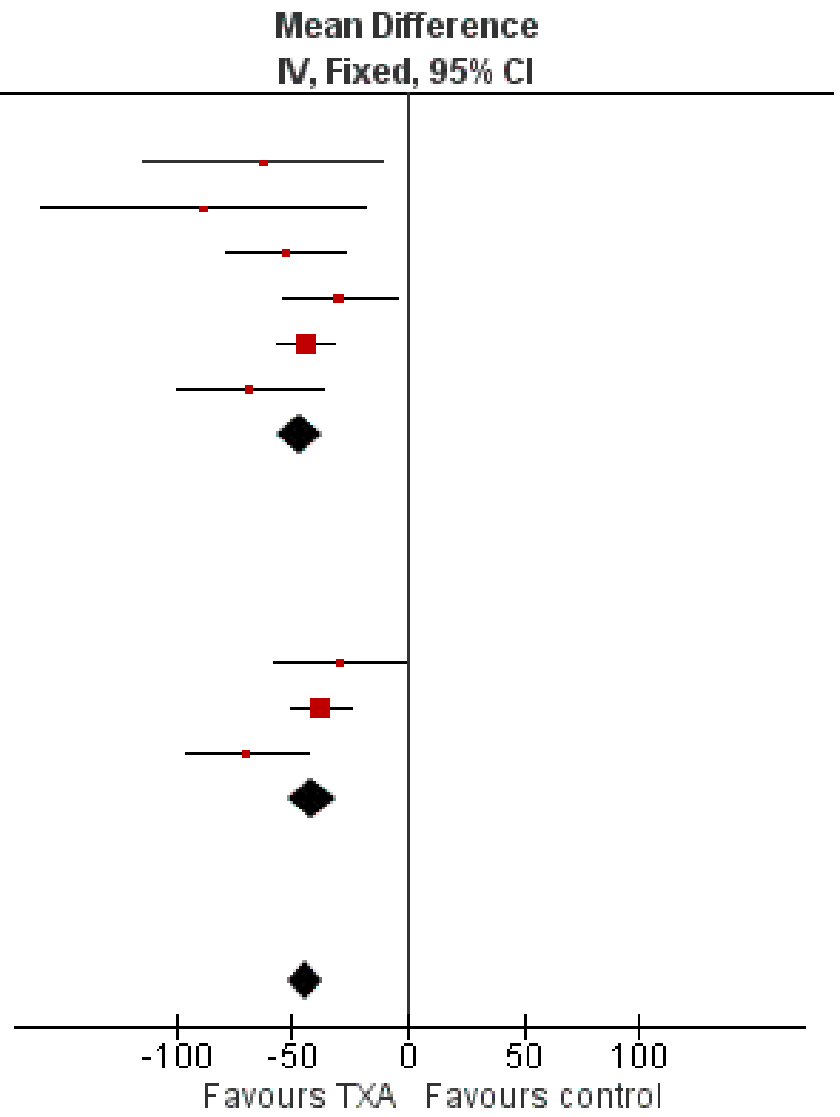

Supplement: Supplementary file 7 [file pone.0362488.s007.pdf]
