# Supplementary material for: Tranexamic acid for anemia in women of reproductive age: A systematic review of randomized trials
Source: PLoS One. 2026 Nov 6;21(11):e0362488. doi: 10.1371/journal.pone.0362488 (PMC13743017; doi:10.1371/journal.pone.0362488)
Supplement: Supplementary file 8 [file pone.0362488.s008.pdf]

Supplementary File 8 – GRADE certainty of evidence assessments *(Berendes et al. (2026), PLOS ONE. DOI: 10.1371/journal.pone.0362488)*

Table S8A – The effect of TXA vs. placebo on primary review outcomes, including Haemoglobin (Hb) levels and Anaemia

Certainty assessment							№ of patients		Anticipated absolute effects* (95% CI)		Relative effect (95% CI)	Certainty
№ of studies	Study design	Risk of bias	Inconsistency	Indirectness	Imprecision	Other considerations	TXA	placebo				
**Haemoglobin (Hb) concentration - Women with HMB (assessed with: venous blood test)**												
4	randomised trials	serious[a]	Not appliable	Not appliable	Not appliable	none[d]	There is no evidence on the effect of TXA vs. placebo on Hb concentrations in women with HMB. Although four trials (one crossover and three parallel-group RCTs) assessed Hb, the available data are insufficient to interpret or pool. Please see Supplementary file 10 for further information.				-	Not appliable (no evidence)
**Haemoglobin (Hb) concentration - Women with IUD (assessed with: venous blood test)**												
2	randomised trials	Serious[a]	Not appliable	Not appliable	Not appliable	none[d]	There is no evidence on the effect of TXA vs. placebo on Hb concentration in menstruating women with IUD. Although two trials (one crossover and one parallel-group RCT) assessed Hb, the available data are insufficient to compute effect estimates and pool. Please see Supplementary file 10 for further information.				-	Not appliable (no evidence)
**Anaemia - Women with HMB (assessed with: venous blood test showing Hb concentration below author-defined threshold)**												
2	randomised trials	Serious[a]	Not appliable	Not appliable	Not appliable	none[d]	There is no evidence on the effect of TXA vs. placebo on anaemia in women with HMB. Although two parallel-group trials recorded some anaemia-related information, the available data/information is insufficient to interpret or pool. Please see Supplementary file 10 for further information.				-	Not appliable (no evidence)
**Anaemia - Women with IUD (assessed with: venous blood test showing Hb concentration below author-defined threshold)**												
1	randomised trials	Serious[a]	Not applicable[b]	Not serious	extremely serious[c]	none[d]	0/27 (0.0%)	3/28 (10.7%)	With placebo: 107 per 1,000	With TXA: 0 per 1,000 (0 to 0)	not estimable	⨁◯◯◯ Very low[d,e,f,g]

CI: confidence interval

Explanations

a. Risk of bias was unclear for several or most domains. (Please see Supplement 7 for details on the risk of bias assessment of included trials.)

b. Cannot be assessed, as single study only.

c. Single trial with very small sample size, few events and extremely wide confidence interval.

d. Although we conducted a comprehensive literature search, we cannot rule out publication bias because funnel plot assessment was not possible.

Table S8B – The effect of TXA vs. placebo on secondary review outcomes – Menstrual Blood Loss (MBL)

Certainty assessment							№ of patients		(Anticipated) Absolute effects* (95% CI)	Relative effect (95% CI)	Certainty
№ of studies	Study design	Risk of bias	Inconsistency	Indirectness	Imprecision	Other considerations	TXA	placebo			
**MBL, objective (ml) - Women with HMB**											
6	randomised trials	serious[a]	not serious	not serious	not serious	none[h]	464	222	MD **47.0 lower** (56.6 lower to 37.4 lower)	-	⊕⊕⊕◯ Moderate[a]
**MBL, objective (ml) - Women with IUD**											
3	randomised trials	serious[a]	not serious[b]	not serious	serious[c]	none[h]	58	61	MD **41.6 lower** (52.2 lower to 30.9 lower)	-	⊕⊕◯◯ Low[a,b,c]
**MBL, subjective - Women with HMB**											
2	randomised trials	serious[d]	not serious	not serious	serious[c]	none[h]	0	0	SMD **0.95 lower** (1.25 lower to 0.65 lower)	-	⊕⊕◯◯ Low[c,d]
**Bleeding duration (days) - Women with HMB**											
3	randomised trials	serious[a]	not serious	not serious	serious[c]	none[h]	54	14	MD **0.3 lower** (1.4 lower to 0.8 higher)	-	⊕⊕◯◯ Low[a,c]

Certainty assessment							№ of patients		(Anticipated) Absolute effects* (95% CI)		Relative effect (95% CI)	Certainty
№ of studies	Study design	Risk of bias	Inconsistency	Indirectness	Imprecision	Other considerations	TXA	placebo				
**Bleeding duration (days) - Women with IUD/ contraceptive**												
4	randomised trials	serious[a]	serious[e]	not serious	serious[c]	none[h]	64	66	MD **1.8 lower** (3.2 lower to 0.4 lower)		-	⊕◯◯◯ Very low[a,c,e]
**Bleeding cessation within 7 days - Women with irregular bleeding due to contraceptive**												
2	randomised trials	serious[f]	serious[g]	not serious	serious[c]	none[h]	70/89 (78.7%)	16/83 (19.3%)	With placebo: 193 per 1,000	With TXA: 798 per 1,000 (499 to 1,000)	**R 4.1** (2.6 to 6.6)	⊕◯◯◯ Very low[c,f,g]
**Number of pads/tampons used - Women with HMB**												
2	randomised trials	serious[a]	not serious	not serious	serious[c]	none[h]	67	28	MD **5.4 lower** (7.9 lower to 2.9 lower)		-	⊕⊕◯◯ Low[a,c]

CI: confidence interval; MD: mean difference; RR: risk ratio; SMD: standardised mean difference,

*The risk in the intervention group (and its 95% confidence interval) is based on the assumed risk in the comparison group and the relative effect of the intervention (and its 95% CI).

Explanations

a. Risk of bias is uncertain for most domains in all or most included trials. Please see Supplementary file 7 for further information.

b. Direction of effect is consistent across trials and confidence intervals overlap. Initial heterogeneity ($I^2 = 64\%$) might be explained by inclusion of a crossover trial; after excluding this trial, $I^2 = 0\%$. Therefore, inconsistency is not considered serious.

c. Downgraded by one level due to low sample size below 300 as per GRADE guidelines 6.

d. Risk of bias is uncertain for random sequence generation and incomplete outcome data in both trials, uncertain for selective reporting in one trial and high in the other, and high in one trial due to lack of blinding.

e. Direction of effect differs across trials and heterogeneity is substantial ($I^2 = 75\%$, remains high after sensitivity analysis). Downgraded by one level for inconsistency.

f. Both included trials have an uncertain risk of bias for selective reporting, and one trial also has an uncertain risk for random sequence generation and allocation concealment.

g. Direction of effect is consistent, but heterogeneity is very high with non-overlapping CIs. Downgraded by one level for inconsistency.

h. We conducted a comprehensive literature search, but we cannot rule out publication bias because the number of studies is small and we could not assess funnel plot asymmetry.

Table S8C – The effect of TXA vs. placebo on secondary review outcomes – Quality of Life (QoL) in women with HMB

Certainty assessment							№ of patients		Absolute effects (95% CI)	Relative effect (95% CI)	Certainty
№ of studies	Study design	Risk of bias	Inconsistency	Indirectness	Imprecision	Other considerations	TXA	placebo			
**Quality of life - Limitations in social or leisure activities**											
2	randomised trials	serious[a]	not serious	not serious	not serious	none[a]	227	138	MD **0.5 higher** (0.4 higher to 0.7 higher)	-	⨁⨁⨁◯ Moderate[a]
**Quality of life - Limitations in physical activities**											
2	randomised trials	serious[a]	not serious	not serious	not serious	none[a]	227	138	MD **0.5 higher** (0.4 higher to 0.7 higher)	-	⨁⨁⨁◯ Moderate[a]

a. Although we conducted a comprehensive literature search, we cannot rule out publication bias because funnel plot assessment was not possible.

Table S8D – The effect of TXA vs. placebo on secondary review outcomes – Side Effects

Certainty assessment							№ of patients		Anticipated absolute effects* (95% CI)		Relative effect (95% CI)	Certainty
№ of studies	Study design	Risk of bias	Inconsistency	Indirectness	Imprecision	Other considerations	TXA	placebo	Risk with placebo	Risk with TXA		
**Pre-specified side effects - Thromboembolic events**												
3	randomised trials	serious[a]	not serious[b]	not serious	extremely serious[c]	none	0/352 (0.0%)	1/146 (0.7%)	7 per 1,000	1 per 1,000 (0 to 34)	**RR 0.2** (0.0 to 5.0)	⊕◯◯◯ Very low[a,b,c]
**Pre-specified side effects - Nausea or vomiting**												
4	randomised trials	serious[d]	not serious	not serious	not serious	none	25/249 (10.0%)	18/199 (9.0%)	90 per 1,000	91 per 1,000 (52 to 161)	**RR 1.0** (0.6 to 1.8)	⊕⊕⊕◯ Moderate[d]

Explanations

*The risk in the intervention group (and its 95% confidence interval) is based on the assumed risk in the comparison group and the relative effect of the intervention (and its 95% CI).

a. All included trials have an uncertain risk of bias for random sequence generation. Two trials have an uncertain risk for selective reporting, while one trial has a high risk for this domain. Allocation concealment is unclear in one trial, and risk of bias relating to incomplete outcome data is unclear in two trials. One trial has a high risk of bias due to lack of blinding.

b. Inconsistency could not be assessed because only one event occurred in the control arm of a single trial, and no events occurred in the intervention or placebo arms of the other trials.

c. Only one event in a single trial with extremely wide confidence interval, and no events in the other trials (effect not estimable).

d. Risk of bias is low in one trial, but all other trials have an uncertain risk of bias in domains.
