# Supplementary material for: Tranexamic acid for anemia in women of reproductive age: A systematic review of randomized trials
Source: PLoS One. 2026 Nov 6;21(11):e0362488. doi: 10.1371/journal.pone.0362488 (PMC13743017; doi:10.1371/journal.pone.0362488)
Supplement: Supplementary file 9 [file pone.0362488.s009.pdf]

## Supplementary file 9 – Results of individual trials

*(Berendes et al. (2026), PLOS ONE. DOI: 10.1371/journal.pone.0362488)*

### Table S9A - Results of Individual Studies Relevant to Primary Review Outcomes or Reasons for Data Not Included

| **Review Outcome & Study** | **Outcome** (detailed) | **TXA arm** n, Mean (SD) or n/N | **Placebo arm** n, Mean (SD) or n/N | **Effect Estimate (95% CI)** | **Note** |
|---|---|---|---|---|---|
| ***Hb concentration - Women with HMB*** | | | | | |
| Drosdal 1993 (crossover) | Hb outcome not usable due to crossover design without washout phase (no first-phase data available) | n/a | n/a | n/a | No washout period; crossover design unsuitable for Hb outcome. No data available for first (pre-crossover) treatment phase.* TXA started on day 2 of menstruation, contrary to best practice. |
| Edlund 1995 (parallel) | Hb assessed, but not reported | n/a | n/a | n/a | No data available* |
| Hamulyák 2024 (parallel) | Hb assessed but not reported; Data on Hb levels at study endpoint obtained from study author. | N=3, Mean (SD) = 8.3 (0.3) g/dL | N=1, Mean (SD)= n/a | n/a | Unpublished data provided by study author. Placebo arm had sample size of n=1, which does not allow calculation of summary statistics or effect estimate. |
| Lukes 2010 (parallel) | Hb outcome interpretability unclear; iron therapy not clearly protocol-defined | n/a | n/a | n/a | Insufficient information on intervention: in addition to TXA and placebo, iron therapy was given "at the investigator's discretion" if Hb<12 g/dL and was required if Hb<11 g/dL at baseline or during the trial. The number of participants receiving iron in each group is unclear.‡ Additionally, more women had anaemia in the TXA arm (34%) than in the placebo arm (18%) at baseline. |
| ***Hb concentration - Women with IUD*** | | | | | |
| Weström 1970 (parallel) | Mean Hb (g/dL) at endpoint (after 3 cycles) | N=27, Mean=12.8 (no SD/SE/p) | N=28, Mean=12.1 (no SD/SE/p) | n/a | Insufficient data to compute effect estimate* |
| Ylikorkala 1983 (crossover) | Hb outcomes not reported by treatment group | n/a | n/a | n/a | Insufficient data: Hb outcomes not reported by group, and not available for first period only.* |

| | | | | | |
|---|---|---|---|---|---|
| **Anaemia – Women with HMB** | | | | | |
| Freeman 2011 (parallel) | Unclear if anaemia outcome was systematically assessed during follow-up | n/a | n/a | n/a | Anaemia was not clearly defined in the study (only severe anaemia, defined as Hb<8 g/dl, was specified and used as an exclusion criterion). Anaemia was not a pre-specified outcome and was reported only as a 'treatment-emergent adverse event'; Hb/anaemia was systematically assessed at baseline, but it was most likely NOT systematically assessed during follow-up or at study endpoint.‡ |
| Lukes 2010 (parallel) | Hb outcome interpretability unclear; iron therapy not clearly protocol-defined | n/a | n/a | n/a | Insufficient information on intervention, as explained for Hb outcome above‡ |
| **Anaemia – Women with IUD** | | | | | |
| Weström 1970 (parallel) | Cumulative number of anaemia cases with Hb<10.5 g/dL at study end (after 3 cycles) | 0/27 | 3/28 | RR=0.15 ~ (0.01,2.74) | |

Footnote: Hb, haemoglobin; HMB, heavy menstrual bleeding; IUD, intrauterine device

* Reasonable attempts were made to locate author contact information and request missing data (maximum of two email attempts). However, no response was received or contact information could not be found, e.g. because the study was published > 30 years ago.

‡ Author responded to requested for further data/information but was unable to provide the requested data (e.g., no longer had access to the dataset or records).

~ Computed in RevMan software (Review Manager version 5.3. Copenhagen: The Cochrane Collaboration, 2014.)

## Table S9B - Results of Individual Studies Relevant to Secondary Review Outcomes or Reasons for Data Not Included

| Review Outcome & Study | Outcome (detailed) | TXA arm<br>n, Mean (SD) or n/N | Placebo arm<br>n, Mean (SD) or n/N | Effect Estimate (95% CI) ~ | Note |
|---|---|---|---|---|---|
| ***Menstrual blood loss (MBL), objective - Women with HMB*** | | | | | |
| Callender 1970 (crossover) | MBL in ml (Oxford total body counter method), mean loss/period, 3 cycles<br>[We computed mean difference (paired) and SE from reported individual-level data] | 16, 122.44 (47.47) | 16, 184.81 (47.47) | MD = -62.37<br>(-113.97, -10.77) | |
| Edlund 1995 (parallel) | MBL in ml (modified AH method), mean 'during treatment' , 3 cycles<br>[We combined means for high- and low-dose TXA and computed SD from range] | 53, 163.14 (119.15) | 14, 251.5 (119.75) | MD= -88.36<br>(-158.81, -17.91) | |
| Freeman 2011 (parallel) | MBL in ml (AH method), mean reduction from baseline, average across 3 cycles<br>[We combined means for high- and low-dose TXA and computed SD from p-value] | 227, -55.76 (96.19) | 67, -3 (96.19) | MD= -52.76<br>(-78.97, -26.55) | Reported p<0.0001 |
| Lukes 2010 (parallel) | MBL in ml (AH method), mean at endpoint, i.e. 6th cycle (also reported: 1st, 2nd, 3rd cycle)<br>[SD computed from p-value] | 115, 104 (83.63) | 72, 134 (83.63) | MD= -30.00<br>(-54.63, -5.37) | Unadjusted for baseline imbalances (MBL was higher in TXA group at baseline); Reported p=0.018 |
| Nilson 1967 (crossover) | "MBL in ml (AH method), mean at endpoint, 1 cycle<br>[We combined means for high- and low-dose TXA and computed mean difference (paired) and SE from reported individual-level data]" | 36, 79.15 (27.71) | 36, 123.41 (27.71) | MD= -44.26<br>(-57.06, -31.46) | |
| Petersen 1983 (crossover) | "MBL in ml (AH method), mean at endpoint, 1 cycle<br>[We computed mean difference (paired) and SE from reported individual-level data]" | 17, 94.0 (46.59) | 17, 162.88 (46.59) | MD -68.88<br>(-100.20, -37.56) | |
| ***MBL, objective - Women with IUD*** | | | | | |
| Lin 2007 (parallel) | "MBL in ml (AH method), mean after 1st cycle (also reported: 2nd and 3rd cycle)<br>[insufficient data to compute SD]" | 39, 61 (SD missing) | 25, 79 (SD missing) | n/a | Not pooled, as insufficient data. |
| Rivera 1984 (parallel) | MBL in ml (AH method), mean increase after IUD insertion at 1 month (also reported: at 2, 3 and 6 months) [SD computed from p-value] | 8, 16 (30.52) | 10, 45.3 (30.52) | MD -29.30<br>(-57.67, -0.93) | Reported p=0.06 |
| Vermylen 1968 (crossover) | "Mean menstrual Hb loss in grams (Perdometer method), mean, 3 cycles | 16, 13.6 (2.69) | 16, 21.2 (2.69) | MD= -7.60<br>(-9.46, -5.74) | Data not included in meta-analyses, as the only article that reported mean menstrual Hb loss (using Perdometer method) |

| | | | | | |
|---|---|---|---|---|---|
| | [We computed mean difference (paired) and SE from reported individual-level data]" | | | | |
| Weström 1970 (parallel) | MBL in ml (AH method), mean after 1st period (also reported: 2nd and 3rd period) [SD computed from t-test result] | 31, 40.8 (25.95) | 32, 78.2 (25.95) | MD -37.40 (-50.19, -24.61) | Reported t=5.72; Unexpectedly heavy flow led to difficulties for some women in 'catching' all the blood in the sanitary napkins and tampons, especially in the group of women receiving the placebos. |
| Ylikorkala 1983 (crossover) | "MBL in ml (AH method), [We computed mean difference (paired) and SE from reported individual-level data]" | 19, 58.61 (41.3) | 19, 128.32 (41.3) | MD= -69.71 (-95.97, -43.45) | |
| ***MBL, subjective - Women with HMB*** | | | | | |
| Drosdal 1993 (crossover) | MBL assessed via visual analogue scale (VAS, but not pooled, as presented data insufficient) | 44 or 37 (n unclear), 31 (SD missing) | 44 or 37 (n unclear), 42 (SD missing) | n/a | Not pooled, as insufficient data. |
| Hamulyak 2024 (parallel) | MBL (PBAC score), mean after randomisation (min 1, max 3 cycles) | 5, 173 (84) | 7, 316 (418) | MD -143.0 (-461.29, 175.29)<br><br>SMD -0.40 (-1.57, 0.76) | MD not pooled, as the only study among women with HMB reporting PBAC score.<br><br>Standardised Mean Difference (SDM) computed in RevMan for pooling of studies with different scales. |
| Lukes 2010 (parallel) | MBL (perceived MBL score), change-from-baseline, | 115, -0.47 (0.59) | 72, 0.15 (0.67) | MD -0.62 (-0.81, -0.43)<br><br>SMD -0.99 (-1.30, -0.68) | MD not pooled, as no comparable study among women with HMB reporting perceived MBL score;<br><br>SDM computed in RevMan for pooling of studies with different scales.<br><br>Unadjusted for baseline imbalances (MBL was higher in TXA group at baseline) |
| ***MBL, subjective - Women with IUD*** | | | | | |
| Lin 2007 (parallel) | MBL in score (PBAC method), mean after 1st cycle (also reported: 2nd and 3rd cycle) [insufficient data to compute SD] | 110, 158 (SD missing) | 65, 188 (SD missing) | n/a | Not pooled, as insufficient data and only study among women with IUD reporting subjective MBL outcome. |
| ***Bleeding duration - Women with HMB*** | | | | | |
| Callender 1970 (crossover) | Duration of menstrual loss (days) [We computed average per menstrual cycle from totals across 3 menstrual cycles, and then mean difference (paired) and SE from reported individual-level data] | 16, 6.08 (0.79) | 16, 5.77 (0.79) | MD 0.31 (-0.24, 0.86) | |
| Edlund 1995 (parallel) | Duration of bleeding (days) [We combined means for high- and low-dose TXA and computed SD from range] | 54, 5.8 (1.36) | 14, 6.1 (2.00) | MD -0.30 (-1.41, 0.81) | |

| | | | | | |
|---|---|---|---|---|---|
| Vermylen 1968 (crossover) | Duration of menstrual bleeding (days) [We computed average per menstrual cycle from totals across 3 menstrual cycles, and then mean difference (paired) and SE from reported individual-level data] | 16, 5.0 (0.83) | 16, 5.1 (0.83) | MD -0.10 (-0.68, 0.48) | |
| Petersen 1983 (crossover) | Bleeding duration (days, assessed but not reported in detail) | NR | NR | NR | Insufficient data - stated only that "there was no difference in the number of bleeding days". |
| ***Bleeding duration - Women with IUD or contraception*** | | | | | |
| Adawiyah 2020 (parallel) | Mean bleeding length at endpoint (28 days post-randomisation) in women with new contraceptive injection (DMPA)- single TXA treatment course of 5 days | 22, 5.2 (3.62) | 22, 9.2 (6.16) | MD -4.00 (-6.99, -1.01) | |
| Phupong 2006 (parallel) | Mean duration of bleeding/spotting days during 28 follow-up period in women with irregular uterine bleeding secondary to contraceptive implant (Norplant) - single TXA treatment course of 5 days. [SE computed from p-value, as no SD/SE presented] | 34, 15.4 (8.25) | 34, 12.7 (8.25) | MD 2.70 (-1.22, 6.62) | Reported p=0.182 |
| Rivera 1984 (parallel) | Women's perception of changes in flow duration (days) at 1 month post-insertion of normal vs TXA-releasing IUD (also reported at 3 and 6 months, when no longer in therapeutic range) [SE computed from p-value, as no SD/SE presented] | 8, 0.4 (1.89) | 10, 2.3 (1.89) | MD -1.90 (-3.66, -0.14) | Reported p<0.05 |
| Ylikorkala 1983 (crossover) | Bleeding length (days), 1st cycle (also reported for 2nd cycle) [did not report individual-level data; assessed as parallel; SD computed from reported SE] | 19, 5.1 (0.44 ) | 19, 5.2 (0.87) | MD -0.10 (-0.54, 0.34) | Reported SE (TXA)=0.1, SE (placebo)=0.2 |
| ***Total number of bleeding/spotting days - Women with IUD or contraception*** | | | | | |
| Sordal 2013 (parallel) | Multivariable analysis of number of bleeding / spotting days during the 90-day period post-IUD insertion using Poisson regression model | | | RR 0.82 (0.65–1.03) | Not pooled, as the only study among women with IUD or contraception reporting total number of bleeding/spotting days. RR as reported in article, favours TXA. |
| ***Cessation of bleeding - Women with IUD or contraception*** | | | | | |
| Phupong 2006 (parallel) | Cessation of bleeding within 7 days after treatment initiation in women with irregular bleeding secondary to contraceptive implant (Norplant) - single TXA treatment course of 5 days. (Also reported maintenance of bleeding-free interval | 22/34 | 12/34 | RR 1.83 (1.09, 3.09) | Reported p=0.015; We computed events from reported percentage. |

| | | | | | |
|---|---|---|---|---|---|
| | >20 days within 4 Weeks, when TXA was no longer used) | | | | |
| Senthong 2009 (parallel) | Cessation of bleeding within first week after treatment initiation in women with irregular uterine bleeding secondary to contraceptive injection (DMPA) - single TXA treatment course of 5 days. (Also reported maintenance of bleeding-free interval >20 days within 4 Weeks, when TXA was no longer used) | 44/50 | 4/49 | RR 10.78 (4.19, 27.73) | Reported p<0.05; We computed events from reported percentage. |
| ***Number of pads/tampons used - Women with HMB*** | | | | | |
| Callender 1970 (crossover) | Number of pads used per cycle [We computed average per menstrual cycle from totals across 3 menstrual cycles, and then mean difference (paired) and SE from reported individual-level data] | 14, 19.8 (3.59) | 14, 24.6 (3.59) | MD -4.8 (-7.46, -2.14) | |
| Drosdal 1983 (crossover) | Average use of pads/tampons during various periods, 3 cycles | 44 or 37 (n unclear), 25 (SD missing) | 44 or 37 (n unclear), 29 (SD missing) | n/a | Data not pooled, as unclear/insufficient data; TXA started only on 2nd day of menstruation |
| Edlund 1995 (parallel) | Number of tampons/pads used during 3 cycles (average per cycle) [We combined means for high- and low-dose TXA and computed SD from range] | 53, 25.8 (8.5) | 14, 36.0 (14.0) | MD -10.20 (-17.88, -2.52) | |
| ***QoL - Limitations in social or leisure activities - Women with HMB*** | | | | | |
| Freeman 2011 (parallel) | Limitations in Social or Leisure Activities associated with HMB (MIQ score, reduction in limitations, positive unit change = improvement), Least Squares mean, change from baseline over 3 cycles | 112, 0.9 (1.0) | 66, 0.3 (0.8) | MD 0.60 (0.33, 0.87) | Least Squares mean and SD retrieved from the trial registration (Outcome 3, NCT00401193) |
| Lukes 2010 (parallel) | Limitations in Social or Leisure Activities associated with HMB (MIQ score, reduction in limitations, positive unit change = improvement), Least Squares mean, change from baseline over 6 cycles | 115, 0.9 (0.9) | 72, 0.4 (0.9) | MD 0.50 (0.23, 0.77) | Data retrieved from trial registration (Outcome 3, NCT00386308) |
| ***Quality of Life (QoL) - Limitations in physical activities - Women with HMB*** | | | | | |
| Freeman 2011 (parallel) | Limitations in Physical Activities associated with HMB (MIQ score, reduction in limitations, positive unit change = improvement), Least Squares mean, change from baseline over 3 cycles | 112, 1.0 (1.1) | 66, 0.4 (0.8) | MD 0.60 (0.32, 0.88) | Least Squares mean and SD retrieved from trial registration (Outcome 2, NCT00401193) |
| Lukes 2010 (parallel) | Limitations in Physical Activities associated with HMB (MIQ score, reduction in limitations, positive unit change = improvement), Least Squares mean, change from baseline over 6 cycles | 115, 0.9 (0.8) | 72, 0.4 (0.4) | MD 0.50 (0.33, 0.67) | Data retrieved from trial registration (Outcome 2, NCT00386308) |

| | | | | | |
|---|---|---|---|---|---|
| ***QoL - Other*** | | | | | |
| Drosdal 1983 (crossover) | QoL assessed, but not reported | NR | NR | NR | |
| Hamulyák 2024 (parallel) | QoL assessed, but not reported | NR | NR | NR | |
| Ylikorkala 1983 (crossover) | QoL, activity restrictions | 1/19 | 0/19 | NR | Reported that "one patient had to restrict her activity during two menstrual periods, out of which one was treated with TA and the other one with DS." (TA= TXA group, DS= other active treatment group) |
| ***Other – absence from work*** | | | | | |
| Petersen 1983 | Number of absences from work assessed but not reported in detail. | NR | NR | NR | Reported only that "There was no difference in the number of [...] absences from work." |
| ***Adverse events – Thromboembolic events*** | | | | | |
| Freeman 2011 (parallel) | Cumulative number of thromboembolic events at study endpoint (3 cycles) | 0/230 | 0/67 | Not estimable | |
| Hamulyák 2024 (parallel) | Cumulative number of thromboembolic events at study endpoint (3 cycles) | 0/5 | 0/7 | Not estimable | Data obtained from author. |
| Lukes 2010 (parallel) | Cumulative number of thromboembolic events at study endpoint (6 cycles) | 0/117 | 1/72 | RR 0.21 (0.01, 4.99) | 1 DVP (deep vein phlebothrombosis) case in control group |
| | | | | | |
| ***Adverse events – Nausea or vomiting*** | | | | | |
| Lukes 2011 (parallel) | Cumulative number of cases of nausea at study endpoint | 17/117 | 11/72 | RR 0.95 (0.47, 1.91) | |
| Nilsson 1967 (crossover) | Cumulative number of cases of nausea (and one case of vomiting) at study endpoint | 4/36 | 3/36 | RR 1.33 (0.32, 5.54) | |
| Senthong 2009 (parallel) | 'Mild degree of nausea/vomiting' during study period (data not presented) | NS/50 | NS/49 | NR | Not pooled, as insufficient data; Stated only that "mild degree of nausea/vomiting was the only adverse effect"; (NS, not specified) |
| Sordal 2013 (parallel) | Cumulative number of cases of nausea at study endpoint | 2/62 | 3/60 | RR 0.65 (0.11, 3.73) | |
| Westrom 1970 (parallel) | Cumulative number of cases of nausea or vomiting at study endpoint | 2/34 | 1/31 | RR 1.82 (0.17, 19.13) | |
| ***Adverse events – Other*** | | | | | |
| Other adverse events not pre-specified in our review protocol are listed in supplementary file 12 | | | | | |

*Footnote:* AH method, alkaline hematin method to determine MBL; CI, confidence interval; Hb, haemoglobin; HMB, heavy menstrual bleeding; IUD, intrauterine device; MBL, menstrual blood loss; MD, mean difference; NR, not reported; NS, not specified; QoL, quality of life; ~ Computed in Review Manager version 5.3. Copenhagen: The Cochrane Collaboration, 2014.
