# Supplementary material for: Tranexamic acid for anemia in women of reproductive age: A systematic review of randomized trials
Source: PLoS One. 2026 Nov 6;21(11):e0362488. doi: 10.1371/journal.pone.0362488 (PMC13743017; doi:10.1371/journal.pone.0362488)
Supplement: Supplementary file 10 [file pone.0362488.s010.pdf]

Supplementary file 10 – Additional Forest plots

Berendes et al. (2026), PLOS ONE. DOI: 10.1371/journal.pone.0362488

Figure S10A1 - Effect of TXA on objective menstrual blood loss (mL) – Random Effects model

	TXA			Control				Mean Difference
**Study or Subgroup**	**Mean**	**SD**	**Total**	**Mean**	**SD**	**Total**	**Weight**	**IV, Random, 95% CI**
**1.1.1 Women with HMB**								
Callender 1970	122.44	74.47	16	184.81	74.47	16	3.3%	-62.37 [-113.97, -10.77]
Edlund 1995	163.14	119.15	53	251.5	119.75	14	1.8%	-88.36 [-158.81, -17.91]
Freeman 2011	-55.76	96.19	227	-3	96.19	67	10.2%	-52.76 [-78.97, -26.55]
Lukes 2010	104	83.63	115	134	83.63	72	11.2%	-30.00 [-54.63, -5.37]
Nilsson 1967	79.15	27.71	36	123.41	27.71	36	23.2%	-44.26 [-57.06, -31.46]
Petersen 1983	94	46.59	17	162.88	46.59	17	7.8%	-68.88 [-100.20, -37.56]
**Subtotal (95% CI)**			**464**			**222**	**57.5%**	**-47.92 [-59.22, -36.61]**
Heterogeneity: Tau² = 27.99; Chi² = 5.73, df = 5 (P = 0.33); I² = 13%								
Test for overall effect: Z = 8.30 (P < 0.00001)								
**1.1.2 Women with IUD**								
Rivera 1984	16	30.52	8	45.3	30.52	10	9.1%	-29.30 [-57.67, -0.93]
Westrom 1970	40.8	25.9	31	78.2	25.9	32	23.2%	-37.40 [-50.19, -24.61]
Ylikorkala 1983	58.61	41.3	19	128.32	41.3	19	10.2%	-69.71 [-95.97, -43.45]
**Subtotal (95% CI)**			**58**			**61**	**42.5%**	**-44.59 [-65.74, -23.43]**
Heterogeneity: Tau² = 222.04; Chi² = 5.54, df = 2 (P = 0.06); I² = 64%								
Test for overall effect: Z = 4.13 (P < 0.0001)								
**Total (95% CI)**			**522**			**283**	**100.0%**	**-46.50 [-56.29, -36.72]**
Heterogeneity: Tau² = 64.90; Chi² = 11.82, df = 8 (P = 0.16); I² = 32%								
Test for overall effect: Z = 9.31 (P < 0.00001)								
Test for subgroup differences: Chi² = 0.07, df = 1 (P = 0.79), I² = 0%								

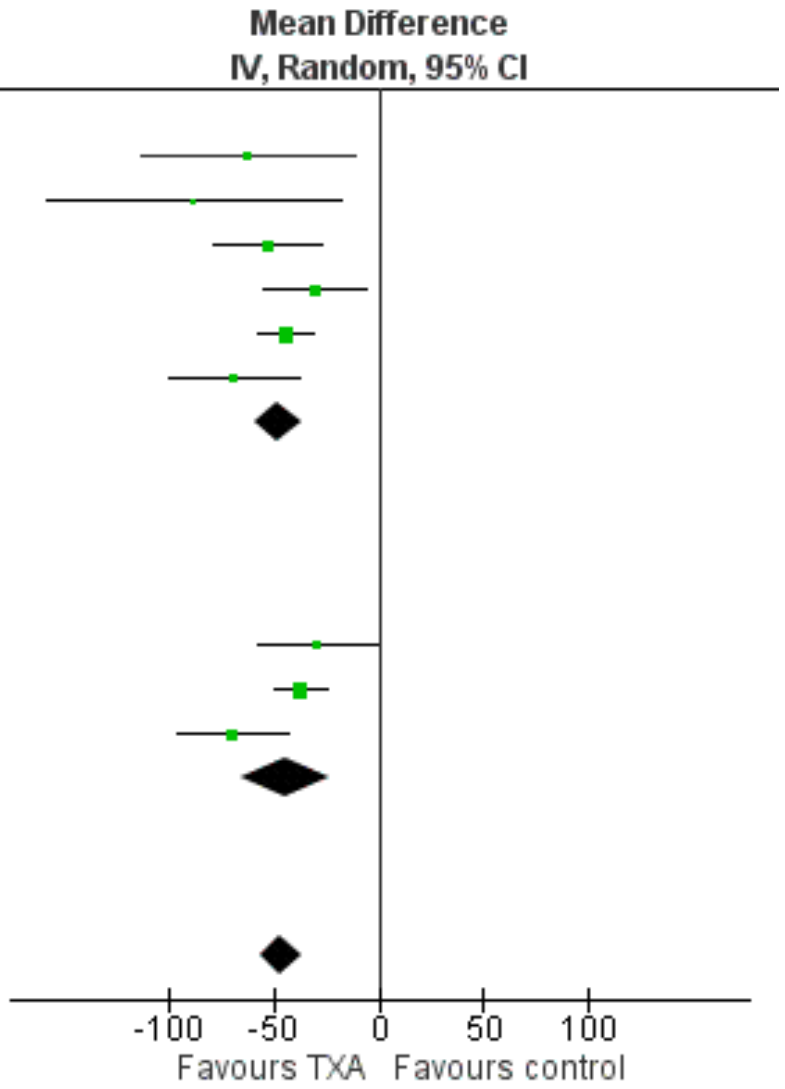

Figure S10A2 - Effect of TXA on obj. menstrual blood loss (mL), <u>excluding crossover studies</u>

	TXA			Control				Mean Difference
**Study or Subgroup**	**Mean**	**SD**	**Total**	**Mean**	**SD**	**Total**	**Weight**	**IV, Fixed, 95% CI**
**1.1.1 Women with HMB**								
Callender 1970	122.44	74.47	16	184.81	74.47	16	0.0%	-62.37 [-113.97, -10.77]
Edlund 1995	163.14	119.15	53	251.5	119.75	14	1.9%	-88.36 [-158.81, -17.91]
Freeman 2011	-55.76	96.19	227	-3	96.19	67	13.7%	-52.76 [-78.97, -26.55]
Lukes 2010	104	83.63	115	134	83.63	72	15.5%	-30.00 [-54.63, -5.37]
Nilsson 1967	79.15	27.71	36	123.41	27.71	36	0.0%	-44.26 [-57.06, -31.46]
Petersen 1983	94	46.59	17	162.88	46.59	17	0.0%	-68.88 [-100.20, -37.56]
**Subtotal (95% CI)**			**395**			**153**	**31.0%**	**-43.58 [-60.98, -26.19]**
Heterogeneity: Chi² = 3.19, df = 2 (P = 0.20); I² = 37%								
Test for overall effect: Z = 4.91 (P < 0.00001)								
**1.1.2 Women with IUD**								
Rivera 1984	16	30.52	8	45.3	30.52	10	11.7%	-29.30 [-57.67, -0.93]
Westrom 1970	40.8	25.9	31	78.2	25.9	32	57.3%	-37.40 [-50.19, -24.61]
Ylikorkala 1983	58.61	41.3	19	128.32	41.3	19	0.0%	-69.71 [-95.97, -43.45]
**Subtotal (95% CI)**			**39**			**42**	**69.0%**	**-36.03 [-47.69, -24.37]**
Heterogeneity: Chi² = 0.26, df = 1 (P = 0.61); I² = 0%								
Test for overall effect: Z = 6.06 (P < 0.00001)								
**Total (95% CI)**			**434**			**195**	**100.0%**	**-38.37 [-48.06, -28.69]**
Heterogeneity: Chi² = 3.95, df = 4 (P = 0.41); I² = 0%								
Test for overall effect: Z = 7.76 (P < 0.00001)								
Test for subgroup differences: Chi² = 0.50, df = 1 (P = 0.48), I² = 0%								

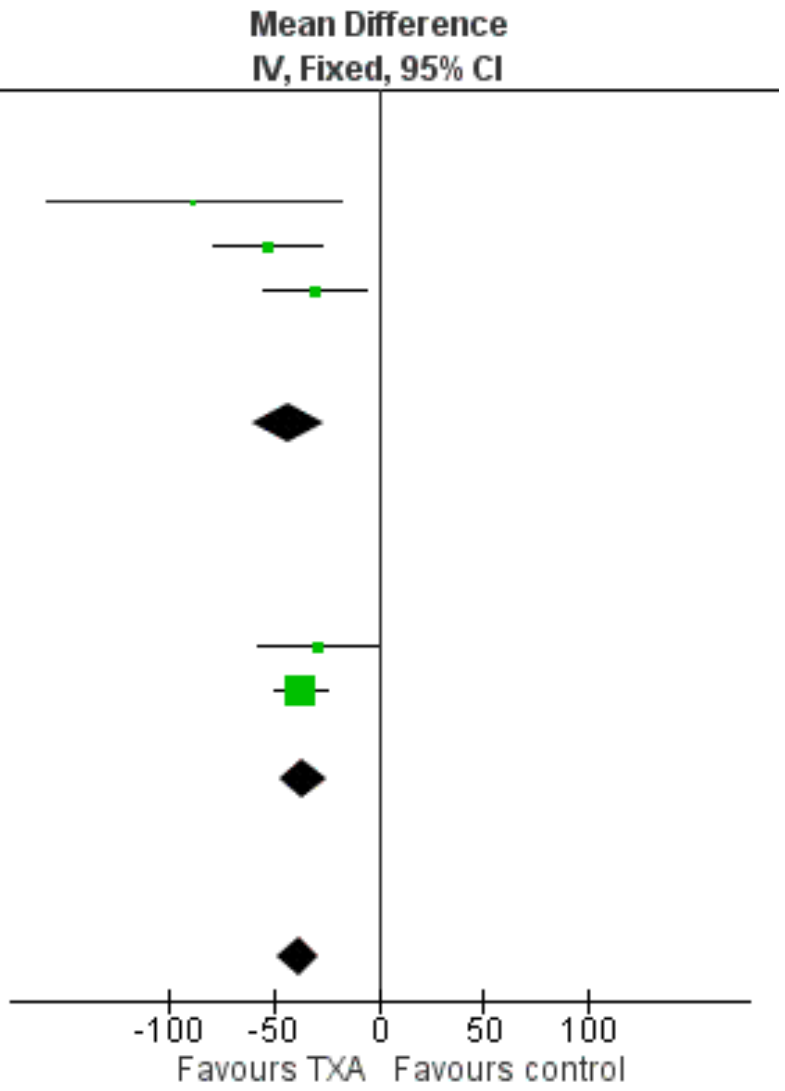

Footnote - Details regarding populations included/excluded in subgroup of women IUD:
Rivera 1984 - Lippes Loop D Intrauterine Device; Westrom 1970 -Lippes' loop B (1 woman), Lippes' loop C (58 women), and Saf-T-Coil 33 S (six women); Ylikorkala 1983 - Copper IUD: n=18, inert IUD: n=1

Figure S10B - Effect of TXA on subjectively measured MBL (standardised mean difference)

Study or Subgroup	Std. Mean Difference	SE	Weight	Std. Mean Difference IV, Fixed, 95% CI
Hamulyak 2024	-0.4023	0.5941	6.7%	-0.40 [-1.57, 0.76]
Lukes 2010	-0.9929	0.159	93.3%	-0.99 [-1.30, -0.68]
**Total (95% CI)**			**100.0%**	**-0.95 [-1.25, -0.65]**

Heterogeneity: Chi² = 0.92, df = 1 (P = 0.34); I² = 0%
Test for overall effect: Z = 6.21 (P < 0.00001)

Std. Mean Difference IV, Fixed, 95% CI

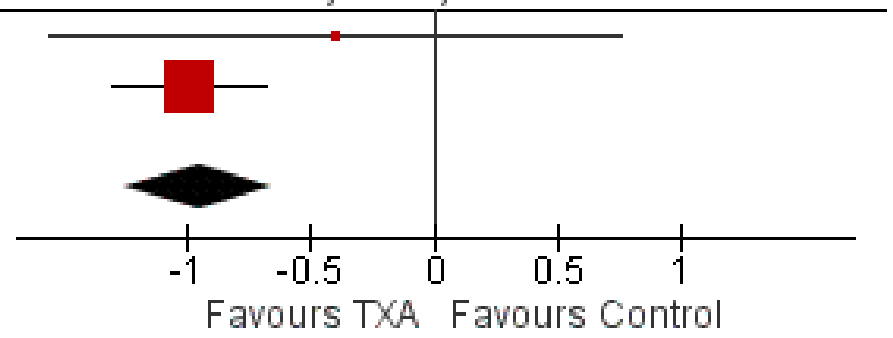

Footnote: Both trials included women with Heavy Menstrual Bleeding (HMB)

Figure S10C1 - Effect of tranexamic acid on bleeding duration (days)

	TXA			Control				Mean Difference
Study or Subgroup	Mean	SD	Total	Mean	SD	Total	Weight	IV, Fixed, 95% CI
**1.3.1 Women with HMB**								
Callender 1970	6.08	0.79	16	5.77	0.79	16	25.9%	0.31 [-0.24, 0.86]
Edlund 1995	5.8	1.36	54	6.1	2	14	6.3%	-0.30 [-1.41, 0.81]
Vermylen 1968	5	0.83	16	5.1	0.83	16	23.5%	-0.10 [-0.68, 0.48]
**Subtotal (95% CI)**			**86**			**46**	**55.7%**	**0.07 [-0.31, 0.44]**
Heterogeneity: Chi² = 1.50, df = 2 (P = 0.47); I² = 0%								
Test for overall effect: Z = 0.36 (P = 0.72)								
**1.3.2 Women with IUD/ contraceptive**								
Adawiyah 2020	5.2	3.62	22	9.2	6.16	22	0.9%	-4.00 [-6.99, -1.01]
Phupong 2006	15.4	8.25	34	12.7	8.25	34	0.5%	2.70 [-1.22, 6.62]
Rivera 1984	0.4	1.8895	8	2.3	1.8895	10	2.5%	-1.90 [-3.66, -0.14]
Ylikorkala 1983	5.1	0.44	19	5.2	0.87	19	40.4%	-0.10 [-0.54, 0.34]
**Subtotal (95% CI)**			**83**			**85**	**44.3%**	**-0.25 [-0.67, 0.17]**
Heterogeneity: Chi² = 12.07, df = 3 (P = 0.007); I² = 75%								
Test for overall effect: Z = 1.16 (P = 0.25)								
**Total (95% CI)**			**169**			**131**	**100.0%**	**-0.07 [-0.35, 0.21]**

Heterogeneity: Chi² = 14.79, df = 6 (P = 0.02); I² = 59%
Test for overall effect: Z = 0.50 (P = 0.61)
Test for subgroup differences: Chi² = 1.21, df = 1 (P = 0.27), I² = 17.5%

Mean Difference IV, Fixed, 95% CI

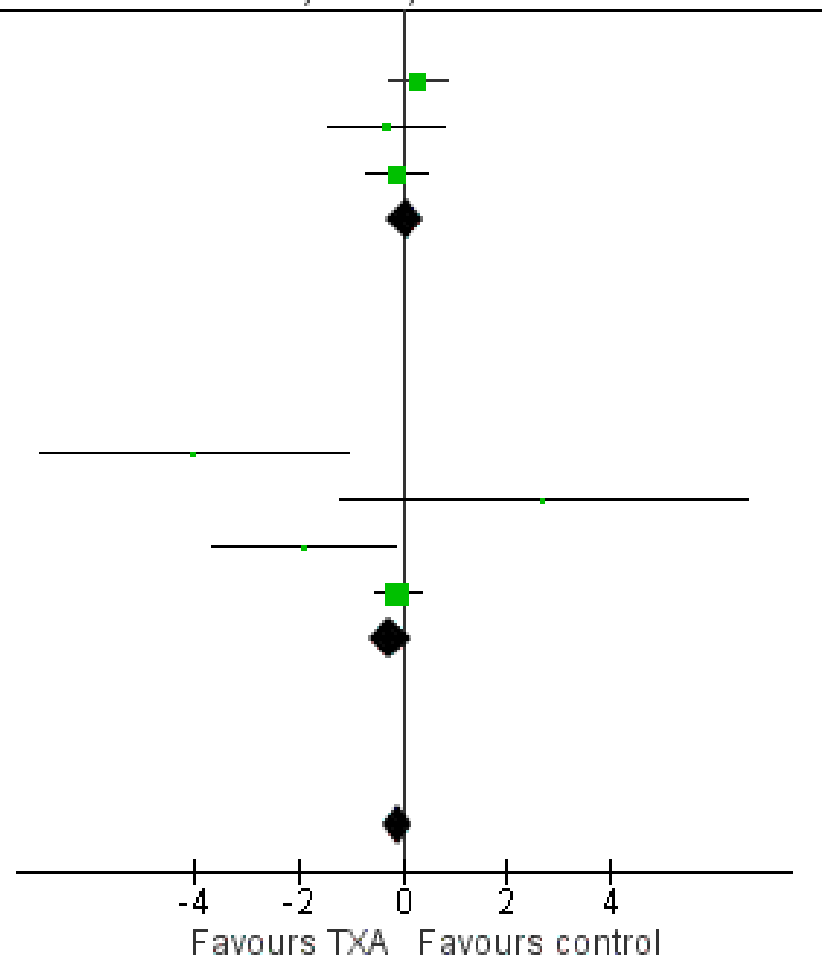

Footnote - Details regarding populations included in subgroup with AUB linked to IUD/contraceptives: Adawiyah 2020 - Women using contraceptive DMPA injection with AUB, defined as bleeding or spotting for ≥ 8 days/month; Phupong (2006) - irregular uterine bleeding secondary to Norplant use (six-capsule levonorgestrel subdermal implants); Rivera 1984 - Lippes Loop D (LLD) Intrauterine Device; Ylikorkala 1983 - Copper IUD: n=18, inert IUD: n=1 (Please see supplementary file 6 and 10 for further details.)

Figure S10C2 - Effect of tranexamic acid on bleeding duration (days) - Random Effects model

	TXA			Control				Mean Difference
Study or Subgroup	Mean	SD	Total	Mean	SD	Total	Weight	IV, Random, 95% CI
**1.3.1 Women with HMB**								
Callender 1970	6.08	0.79	16	5.77	0.79	16	24.0%	0.31 [-0.24, 0.86]
Edlund 1995	5.8	1.36	54	6.1	2	14	13.8%	-0.30 [-1.41, 0.81]
Vermylen 1968	5	0.83	16	5.1	0.83	16	23.5%	-0.10 [-0.68, 0.48]
**Subtotal (95% CI)**			**86**			**46**	**61.3%**	**0.07 [-0.31, 0.44]**
Heterogeneity: Tau² = 0.00; Chi² = 1.50, df = 2 (P = 0.47); I² = 0%								
Test for overall effect: Z = 0.36 (P = 0.72)								
**1.3.2 Women with IUD/ contraceptive**								
Adawiyah 2020	5.2	3.62	22	9.2	6.16	22	3.1%	-4.00 [-6.99, -1.01]
Phupong 2006	15.4	8.25	34	12.7	8.25	34	1.9%	2.70 [-1.22, 6.62]
Rivera 1984	0.4	1.8895	8	2.3	1.8895	10	7.5%	-1.90 [-3.66, -0.14]
Ylikorkala 1983	5.1	0.44	19	5.2	0.87	19	26.3%	-0.10 [-0.54, 0.34]
**Subtotal (95% CI)**			**83**			**85**	**38.7%**	**-0.97 [-2.89, 0.94]**
Heterogeneity: Tau² = 2.52; Chi² = 12.07, df = 3 (P = 0.007); I² = 75%								
Test for overall effect: Z = 1.00 (P = 0.32)								
**Total (95% CI)**			**169**			**131**	**100.0%**	**-0.23 [-0.78, 0.32]**

Heterogeneity: Tau² = 0.25; Chi² = 14.79, df = 6 (P = 0.02); I² = 59%
Test for overall effect: Z = 0.82 (P = 0.41)
Test for subgroup differences: Chi² = 1.10, df = 1 (P = 0.29), I² = 9.0%

Mean Difference IV, Random, 95% CI

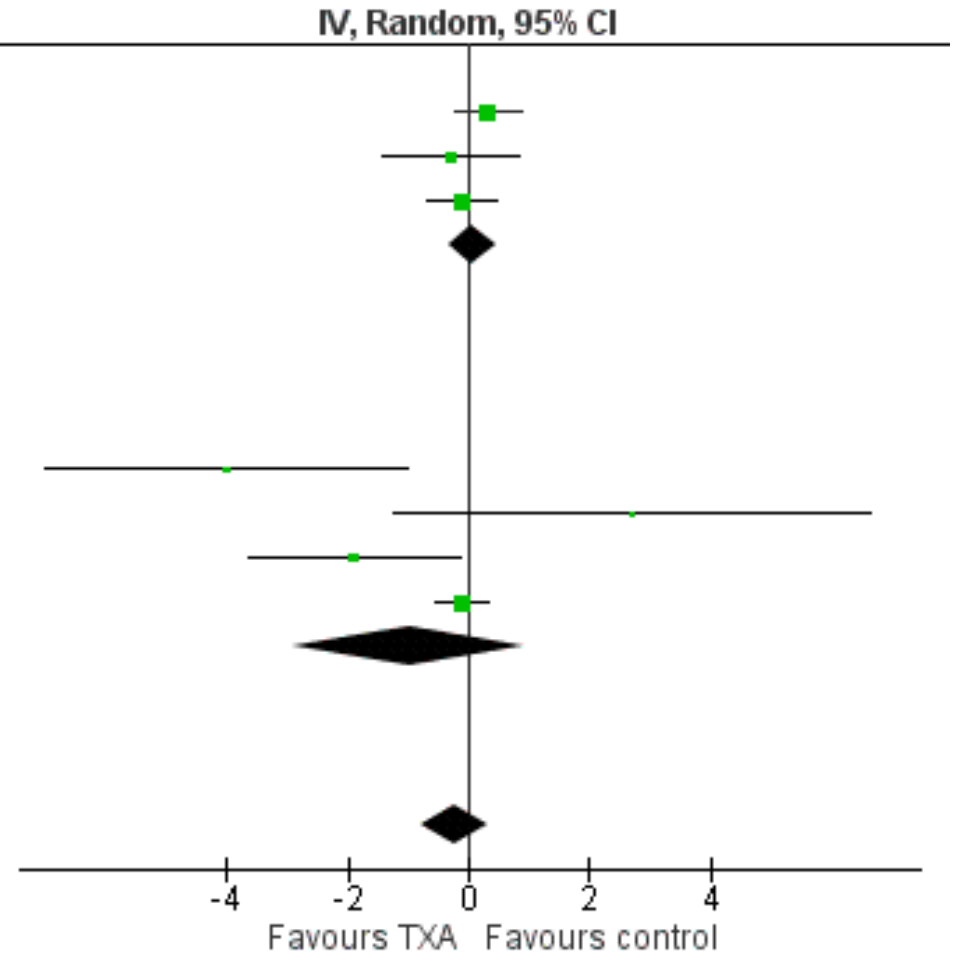

Figure S10D: Effect of TXA on Bleeding duration, excluding crossover studies

	TXA			Control				Mean Difference
**Study or Subgroup**	**Mean**	**SD**	**Total**	**Mean**	**SD**	**Total**	**Weight**	**IV, Fixed, 95% CI**
**1.3.1 Women with HMB**								
Callender 1970	6.08	0.79	16	5.77	0.79	16	0.0%	0.31 [-0.24, 0.86]
Edlund 1995	5.8	1.36	54	6.1	2	14	61.9%	-0.30 [-1.41, 0.81]
Vermylen 1968	5	0.83	16	5.1	0.83	16	0.0%	-0.10 [-0.68, 0.48]
**Subtotal (95% CI)**			**54**			**14**	**61.9%**	**-0.30 [-1.41, 0.81]**
Heterogeneity: Not applicable								
Test for overall effect: Z = 0.53 (P = 0.60)								
**1.3.2 Women with IUD/ contraceptive**								
Adawiyah 2020	5.2	3.62	22	9.2	6.16	22	8.5%	-4.00 [-6.99, -1.01]
Phupong 2006	15.4	8.25	34	12.7	8.25	34	4.9%	2.70 [-1.22, 6.62]
Rivera 1984	0.4	1.8895	8	2.3	1.8895	10	24.6%	-1.90 [-3.66, -0.14]
Ylikorkala 1983	5.1	0.44	19	5.2	0.87	19	0.0%	-0.10 [-0.54, 0.34]
**Subtotal (95% CI)**			**64**			**66**	**38.1%**	**-1.77 [-3.19, -0.36]**
Heterogeneity: Chi² = 7.15, df = 2 (P = 0.03); I² = 72%								
Test for overall effect: Z = 2.46 (P = 0.01)								
**Total (95% CI)**			**118**			**80**	**100.0%**	**-0.86 [-1.73, 0.01]**
Heterogeneity: Chi² = 9.74, df = 3 (P = 0.02); I² = 69%								
Test for overall effect: Z = 1.94 (P = 0.05)								
Test for subgroup differences: Chi² = 2.59, df = 1 (P = 0.11), I² = 61.3%								

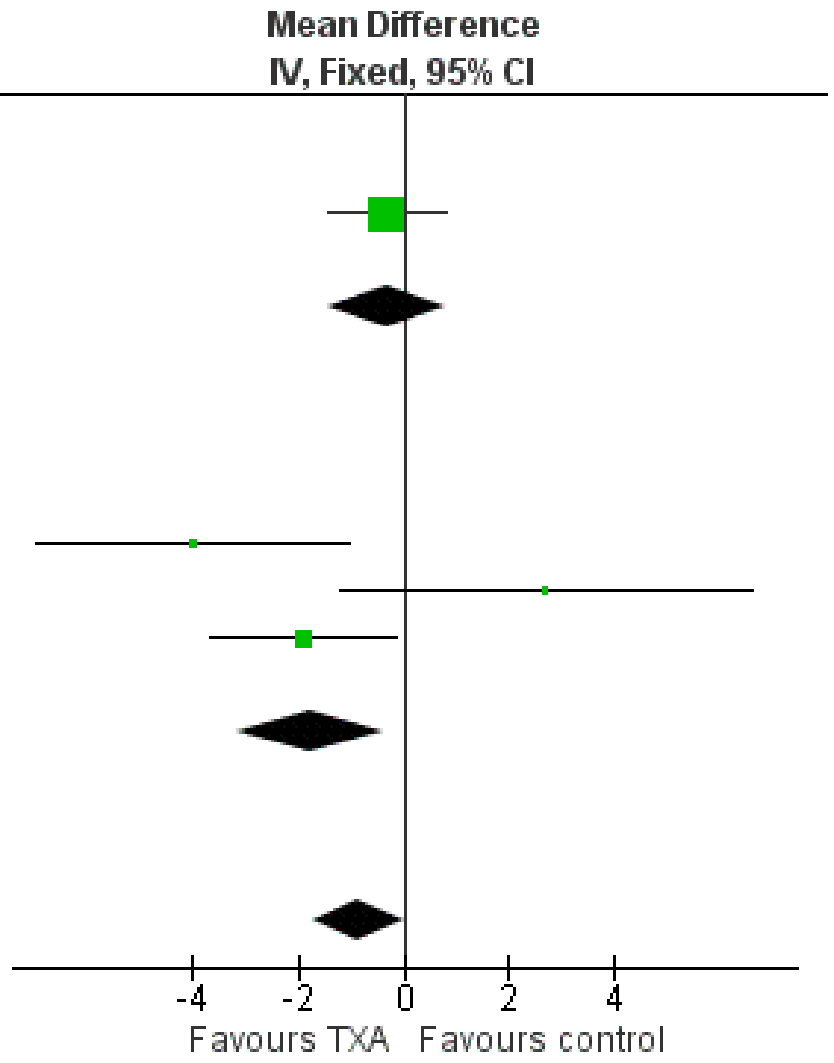

Footnote - Details regarding populations included in subgroup with AUB linked to IUD/contraceptives: Adawiyah 2020 - Women using contraceptive DMPA injection with AUB, defined as bleeding or spotting for ≥ 8 days/month; Phupong (2006) - irregular uterine bleeding secondary to Norplant use (six-capsule levonorgestrel subdermal implants); Rivera 1984 - Lippes Loop D (LLD) Intrauterine Device; Ylikorkala 1983 - Copper IUD: n=18, inert IUD: n=1 (Please see supplementary file 6 and 10 for further details.)

Figure S10E: Effect of TXA on bleeding cessation

	TXA		Control			Risk Ratio
**Study or Subgroup**	**Events**	**Total**	**Events**	**Total**	**Weight**	**M-H, Fixed, 95% CI**
Phupong 2006	22	34	12	34	74.8%	1.83 [1.09, 3.08]
Senthong 2009	44	50	4	49	25.2%	10.78 [4.19, 27.73]
**Total (95% CI)**		**84**		**83**	**100.0%**	**4.09 [2.57, 6.51]**
Total events	66		16			
Heterogeneity: Chi² = 13.23, df = 1 (P = 0.0003); I² = 92%						
Test for overall effect: Z = 5.93 (P < 0.00001)						

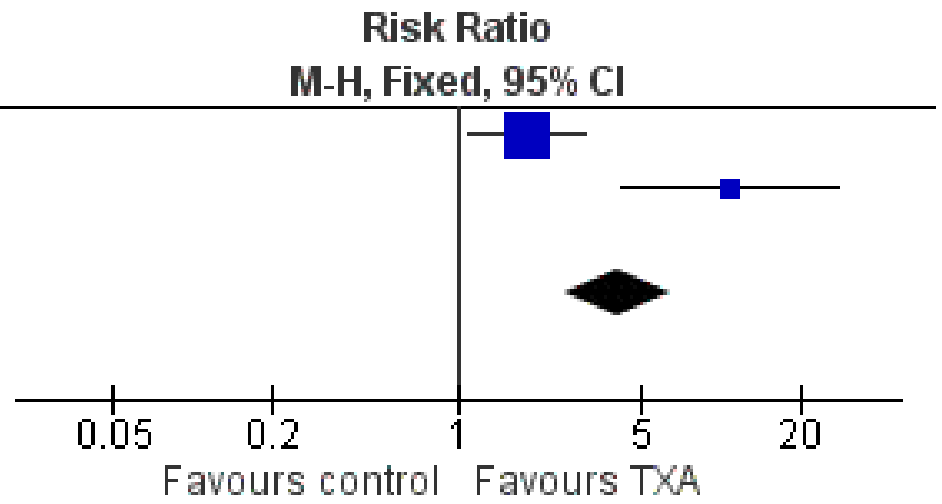

Footnote – Details on populations included: Phupong 2006 - Women with irregular uterine bleeding secondary to Norplant use (six-capsule levonorgestrel subdermal implants, a long-acting progestine); Senthong 2009 - Women with irregular uterine bleeding secondary to contraceptive injection (Depot-medroxyprogesterone acetate, DMPA, a long acting progestine)

Figure S10F: Effect of TXA on number of pads/tampons used

	TXA			Control				Mean Difference
**Study or Subgroup**	**Mean**	**SD**	**Total**	**Mean**	**SD**	**Total**	**Weight**	**IV, Fixed, 95% CI**
Callender 1970	19.8	3.59	14	24.6	3.59	14	89.3%	-4.80 [-7.46, -2.14]
Edlund 1995	25.8	8.48	53	36	14	14	10.7%	-10.20 [-17.88, -2.52]
**Total (95% CI)**			**67**			**28**	**100.0%**	**-5.38 [-7.89, -2.87]**
Heterogeneity: Chi² = 1.70, df = 1 (P = 0.19); I² = 41%								
Test for overall effect: Z = 4.19 (P < 0.0001)								

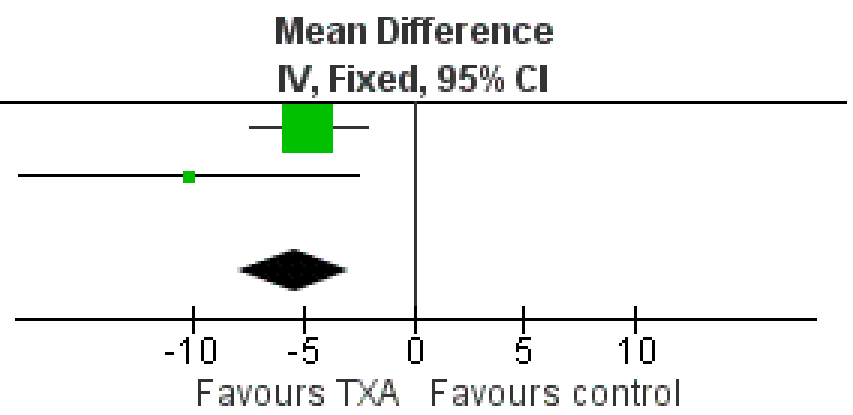

Footnote: Both trials included women with Heavy Menstrual Bleeding (HMB)
