# Supplementary material for: Tranexamic acid for anemia in women of reproductive age: A systematic review of randomized trials
Source: PLoS One. 2026 Nov 6;21(11):e0362488. doi: 10.1371/journal.pone.0362488 (PMC13743017; doi:10.1371/journal.pone.0362488)
Supplement: Supplementary file 11 [file pone.0362488.s011.pdf]

Supplementary File 11 – List of other side effects

(Berendes et al. (2026), PLOS ONE. DOI: 10.1371/journal.pone.0362488)

A - List of other side effects not pre-specified in systematic review registration

	TXA		Control	
	**n**	**N**	**n**	**N**
Upper respiratory tract infection				
**Freeman 2011**	20	230	3	67
**Lukes 2011**	9	117	7	72
Infections and infestations				
**Sordal 2013**	7	62	6	60
Throat infection				
**Freeman 2011**	7	230	2	67
Fatigue				
**Freeman 2011**	17	230	3	67
**Lukes 2011**	8	117	3	72
Musculoskeletal pain				
**Freeman 2011**	16	230	2	67
Arthalgia/joint pain				
**Freeman 2011**	12	230	1	67
**Lukes 2011**	11	117	5	72
Myalgia/ muscle pain				
**Freeman 2011**	11	230	0	67
Nasal congestion				
**Freeman 2011**	11	230	0	67
Sinusitis				
**Freeman 2011**	10	230	1	67
Multiple allergies				
**Freeman 2011**	10	230	0	67
**Lukes 2011**	10	117	5	72
Back pain				
**Lukes 2011**	28	117	14	72
Dizziness or nausea				
**Adawiyah 2020 ^**	5	22	1	22

Dizziness/light-headed feelings'/'haziness'				
**Nilsson 1967 ***	1	36	0	36
**Callender 1970**	0	16	2	16
Abdominal discomfort or pain				
**Lukes 2011**	8	117	6	72
**Nilsson 1967 ***	7	36	1	36
**Sordal 2013**	7	62	5	60
Dyspepsia/ heartburn				
**Lukes 2011**	3	117	8	72
**Nilsson 1967 ***	0	36	1	36
Cough				
**Lukes 2011**	7	117	5	72
Insomnia				
**Lukes 2011**	6	117	6	72
Muscle cramps				
**Lukes 2011**	8	117	3	72
Headaches				
**Callender 1970**	1	16	0	16
**Lukes 2011**	65	117	36	72
**Nilsson 1967 ***	1	36	2	36
**Sordal 2013**	5	62	7	60
Migraine				
**Lukes 2011**	7	117	4	72
Sinus headache				
**Lukes 2011**	9	117	2	72
Diarrhoea				
**Nilsson 1967 ***	6	36	0	36
**Westrom 1970**	1	34	0	31
Exanthema (rash plus fever)				
**Nilsson 1967 ***	1	36	0	36
***"Urgency"** (term not further specified in report)*				
**Nilsson 1967 ***	1	36	0	36
Musculoskeletal and connective tissue disorders				

**Sordal 2013**	4	62	2	60
Psychiatric disorders				
**Sordal 2013**	3	62	1	60
Reproductive system and breast disorders *(dysmenorrhea, pelvic pain, breast tenderness)*				
**Sordal 2013**	13	62	15	60
Menstrual cramps/discomfort				
**Lukes 2011**	72	117	36	72
Dysmenorrhea				
**Sordal 2013**	8	62	10	60
**Rivara 1984**	6	10	9	11
Skin and subcutaneous tissue disorders				
**Sordal 2013**	0	62	4	60
Gastrointestinal discomfort (translation from Norwegian)				
**Drosdal 1983**	2	44	2	44
Ocular-related adverse effect				
**Lukes**	2	117	5	72
Facial flushing				
**Westrom 1970**	1	34	1	31
Feeling 'sickly' or 'unwell'				
**Callender 1970**	2	16	1	16

^ Reported p=0.185; * Crossover trial; adverse events summarized by treatment arm as if parallel

B - Further information on side effects extracted from trial reports:

Freeman 2011: "A total of 257 subjects (86.5%) reported treatment-emergent AEs with no significant difference among the 3 treatment groups."

Edlund 1995: "The number of reported adverse events did not differ between the treatment and the placebo groups. Reported adverse events included gastrointestinal symptoms, headache, and dry mouth -symptoms commonly associated with tranexamic acid."

Hamulyak 2024: "No serious adverse events were recorded"

Lukes 2010: No statistically significant difference in adverse events between trial arms; All events rated as 'serious adverse events' (n=6) during the study, were 'judged to be unrelated to study treatment'.

Peterson 1983: "No women had side effects from treatment with tranexamic acid."

Phupong 2006: "There was no detectable adverse effect in both groups."

Rivera 1984: “There were no removals [of IUDs] for bleeding, pain or other medical reasons in either group. One woman in each group became pregnant with the device in situ; both were intrauterine pregnancies and were successfully carried to term. Two expulsions occurred during the study, one within the first month and the other in the sixth month post-insertion, both of which were AMCA LLD devices.”

Senthong 2009: "The present study also demonstrated that mild degree of nausea/vomiting was the only adverse effect in the present study."

Vermylen 1968: Side effects reported as total number of ‘days’ (not patients) a side effect was noted for various types of side effects; "It appears that there is no marked difference in the frequency of side effects between the 2 treatments."

Ylikorkala 1983: "TA treatment caused mostly gastrointestinal disturbances (diarrhoea, lower abdominal pains), but also headache (two periods) and sweating (one period). No patients discontinued the TA therapy because of side effects."
